# Update of Guidelines for laparoscopic treatment of ventral and incisional abdominal wall hernias (International Endohernia Society (IEHS))—Part A

**DOI:** 10.1007/s00464-019-06907-7

**Published:** 2019-06-27

**Authors:** R. Bittner, K. Bain, V. K. Bansal, F. Berrevoet, J. Bingener-Casey, D. Chen, J. Chen, P. Chowbey, U. A. Dietz, A. de Beaux, G. Ferzli, R. Fortelny, H. Hoffmann, M. Iskander, Z. Ji, L. N. Jorgensen, R. Khullar, P. Kirchhoff, F. Köckerling, J. Kukleta, K. LeBlanc, J. Li, D. Lomanto, F. Mayer, V. Meytes, M. Misra, S. Morales-Conde, H. Niebuhr, D. Radvinsky, B. Ramshaw, D. Ranev, W. Reinpold, A. Sharma, R. Schrittwieser, B. Stechemesser, B. Sutedja, J. Tang, J. Warren, D. Weyhe, A. Wiegering, G. Woeste, Q. Yao

**Affiliations:** 1 I.M. Sechenov First Moscow State Medical University of the Ministry of Health of the Russian Federation (Sechenov University), Trubetskaya str., 8, b. 2, 119992 Moscow, Russia; 2 Emeritus Director Marienhospital Stuttgart, Supperstr. 19, 70565 Stuttgart, Germany; 3 Department of Surgery, New York University, New York, USA; 4 Department of Surgical Disciplines, All India Institute of Medical Sciences, Room No. 5026A, 5th Floor, Teaching Block, Ansari Nagar, New Delhi, 110029 India; 5 Universitair Ziekenhuis Gent, C. Heymanslaan 10, 9000 Ghent, Belgium; 6 Division of Breast, Endocrine, Metabolic & Gastrointestinal Surgery, Mayo Clinic, 200 First Street SW, Rochester, MN 55905 USA; 7 Lichtenstein Amid Hernia Clinic at UCLA, Section of Minimally Invasive Surgery, UCLA Division of General Surgery, Los Angeles, USA; 8 Department of Hernia and Abdominal Surgery, Beijing Chao-Yang Hospital, Capital Medical University, Fengtai, China; 9 Max Super Speciality Hospital, 2 Press Enclave Road, Saket, New Delhi, 110017 India; 10 Klinik für Viszeral-, Gefäss- und Thoraxchirurgie, Kantonsspital Olten, Baslerstrasse 150, 4600 Olten, Switzerland; 11 Royal Infirmary of Edinburgh, Edinburgh, EH16 4SA UK; 12 Allgemein-, Viszeral- und Tumorchirurgie, Wilhelminenspital, 1160 Vienna, Austria; 13 ZweiChirurgen GmbH, Zentrum für Hernienchirurgie und Proktologie, St. Johanns-Vorstadt 44, 4056 Basel, Switzerland; 14 Department of Surgery, Mount Sinai Hospital, 1010 5th Avenue, New York, NY 10028 USA; 15 Department of Surgery, Southeast University School of Medicine, Main Add. 87 Ding Jia Qiao, Nanjing, 210009 Jiangsu China; 16 Digestive Disease Center, Bispebjerg Hospital, University of Copenhagen, 2400 Copenhagen NV, Denmark; 17 Visceral- und Gefäßchirurgie, Zentrum für Minimal Invasive Chirurgie, Vivantes Klinikum Spandau, Neue Bergstraße 6, 13585 Berlin, Germany; 18 Klinik im Park, Grossmuensterplatz 9, 8001 Zurich, Switzerland; 19 Our Lady of the Lake Physician Group, 7777 Hennessy Blvd., Suite 612, Baton Rouge, LA 70808 USA; 20 Department of General Surgery, Ruijin Hospital, Shanghai Jiao Tong University School of Medicine, Shanghai, 200025 China; 21 Department of Surgery, YLL School of Medicine, National University Hospital, Level 2, Kent Ridge Wing 2, 5 Lower Kent Ridge Road, Singapore, 119074 Singapore; 22 Paracelsus Medizinische Universität Salzburg (PMU), Universitätsklinik für Chirurgie, Salzburg, Austria; 23 Mahatma Gandhi University of Medical Sciences & Technology, RIICO Institutional Area, Tonk Road, Sitapura, Jaipur, Rajasthan 302 022 India; 24 Centro de Cirugía Mayor Ambulatoria Ave María, Avda. de la Palmera, 53, 41013 Seville, Spain; 25 HANSECHIRURGIE, Niebuhr Marleschki & Partner, Alte Holstenstr. 16, 21031 Hamburg, Germany; 26 SUNY Downstate Medical Center, 450 Clarkson Avenue, Brooklyn, NY 11203 USA; 27 Department of Surgery, University Surgeons Associates, 1930 Alcoa Highway, Bldg A, Ste 285, Knoxville, TN 37920 USA; 28 Lenox Hill Hospital-Northwell Health, New York, USA; 29 Abteilung für Chirurgie, Wilhelmsburger Krankenhaus, Groß-Sand 3, 21107 Hamburg, Germany; 30 Abteilung für Chirurgie, LKH Hochsteiermark, Standort Bruck an der Mur Tragösser Str. 1, 8600 Bruck an der Mur, Austria; 31 Hernienzentrum Köln, Zeppelinstraße 1, 50667 Cologne, Germany; 32 Gading Pluit Hospital, Jl. Boulevard Timur Raya Kelapa Gading, Jakarta, 14250 Indonesia; 33 Department of General Surgery, Huadong Hospital, Fudan University, Shanghai, China; 34 Minimally Invasive Surgery, Greenville Health System, Department of Surgery, University of South Carolina School of Medicine, Greenville, USA; 35 Pius-Hospital Oldenburg, Klinik für Allgemein- und Viszeralchirurgie, Universitätsklinik für Viszeralchirurgie, Georgstraße 12, 26121 Oldenburg, Germany; 36 Department of General, Visceral, Vascular and Paediatric Surgery, University Hospital of Wuerzburg, Oberduerrbacher Strasse 6, 97080 Würzburg, Germany; 37 AGAPLESION ELISABETHENSTIFT gemeinnützige GmbH, Akademisches Lehrkrankenhaus, Landgraf-Georg-Strasse 100, 64287 Darmstadt, Germany; 38 Department of Hernia and Abdominal Surgery, Huashan Hospital, Fudan University, Shanghai, China

**Keywords:** Update guidelines, abdominal wall hernia, Ventral hernia repair, Primary ventral hernias, Secondary ventral hernias, Open sublay repair, Endoscopic sublay, Laparoscopic repair, IPOM, Rectus diastasis, Milos, Emilos, eTEP

## Abstract

**Abstract:**

In 2014, the International Endohernia Society (IEHS) published the first international “Guidelines for laparoscopic treatment of ventral and incisional abdominal wall hernias.” Guidelines reflect the currently best available evidence in diagnostics and therapy and give recommendations to help surgeons to standardize their techniques and to improve their results. However, science is a dynamic field which is continuously developing. Therefore, guidelines require regular updates to keep pace with the evolving literature.

**Methods:**

For the development of the original guidelines, all relevant literature published up to year 2012 was analyzed using the ranking of the Oxford Centre for Evidence-Based Medicine. For the present update, all of the previous authors were asked to evaluate the literature published during the recent years from 2012 to 2017 and revise their statements and recommendations given in the initial guidelines accordingly. In two Consensus Conferences (October 2017 Beijing, March 2018 Cologne), the updates were presented, discussed, and confirmed. To avoid redundancy, only new statements or recommendations are included in this paper. Therefore, for full understanding both of the guidelines, the original and the current, must be read. In addition, the new developments in repair of abdominal wall hernias like surgical techniques within the abdominal wall, release operations (transversus muscle release, component separation), Botox application, and robot-assisted repair methods were included.

**Results:**

Due to an increase of the number of patients and further development of surgical techniques, repair of primary and secondary abdominal wall hernias attracts increasing interests of many surgeons. Whereas up to three decades ago hernia-related publications did not exceed 20 per year, currently this number is about 10-fold higher. Recent years are characterized by the advent of new techniques—minimal invasive techniques using robotics and laparoscopy, totally extraperitoneal repairs, novel myofascial release techniques for optimal closure of large defects, and Botox for relaxing the abdominal wall. Furthermore, a concomitant rectus diastasis was recognized as a significant risk factor for recurrence. Despite insufficient evidence with respect to these new techniques, it seemed to us necessary to include them in the update to stimulate surgeons to do research in these fields.

**Conclusion:**

Guidelines are recommendations based on best available evidence intended to help the surgeon to improve the quality of his daily work. However, science is a continuously evolving process, and as such guidelines should be updated about every 3 years. For a comprehensive reference, however, it is suggested to read both the initial guidelines published in 2014 together with the update. Moreover, the presented update includes also techniques which were not known 3 years before.

## Content—Part A

Chapter 1: How comparable are incisional and ventral hernias in terms of operative technique and outcomes?

Chapter 2a: Is the routine application of computed tomography (CT) and magnetic resonance imaging (MRI) recommended for the diagnosis of ventral hernias before laparoscopic ventral hernia repair?

Chapter 2b: Can the routine application of ultrasound imaging be helpful in detecting ventral hernias and rectus diastasis preoperatively?

Chapter 3: Classification

Chapter 4: Indications for treatment dependence on size of defect or hernia sac, hernia type, symptoms, and age.

Chapter 5: Is there still a place for open suture repair depending on defect size?

Chapter 6: Obese patients and incisional hernia

Chapter 7: Recurrence after open surgery—re-do better laparoscopically?

Chapter 8: Evidence for antibiotic and thromboembolic prophylaxis in laparoscopic ventral hernia surgery

Chapter 9: Positioning of the trocars and creating the capno pneumoperitoneumc

Chapter 10: Port type, positions, and number in laparoscopic ventral hernia repair

Chapter 11: Principles of adhesiolysis

Chapter 12: Laparoscopic ventral or incisional hernia repair—importance of defining hernial defect margins and gaging the size of the hernia preoperatively and intraoperatively

Chapter 13: Bridging–augmentation–reconstruction of the linea alba—closure of the defect before IPOM

Chapter 14: How much overlap is necessary?

Chapter 15/16: Fixation

Chapter 17: Mesh insertion

Chapter 18: Management of bowel injury during laparoscopic ventral incisional hernia repair

Chapter 19: Risk factors for infection in laparoscopic incisional/ventral hernia repair

Chapter 20: Mesh Infection

Chapter 21: Postoperative Seroma: Risk Factors, Prevention and Best Treatment

Chapter 22: Postoperative bulging

Chapter 23: Chronic pain—risk factors, prevention, and treatment

Chapter 24: Recurrence after laparoscopic ventral/incisional hernia repair—risk factors, mechanism, and prevention.

Chapter 25: Comparison of open vs. laparoscopic hernia repair: OR time, bowel lesion, seroma, and wound infection

Chapter 26: Comparison of hospital stay, return to activity, cost, quality of life, pain, and recurrence after laparoscopic and open ventral and incisional hernia repair

Chapter 27: Do we have an ideal mesh in terms of prevention of adhesions? Are coated meshes really necessary? Are there data to support the manufacturers’ claims of superiority? Is permanent or absorbable barrier preferred?

Chapter 28: Role of biological/biosynthetic meshes in laparoscopic incisional and ventral hernia repair? Are they advantageous in infected abdominal wall?

Chapter 29: What happens to synthetic mesh after it is inserted into the body?

Chapter 30: Open abdominal surgery and stoma surgery: indications for prophylactic mesh implantation and risk reduction strategies

Chapter 31: NOTES and Single-Port Surgery: Is there currently any role in ventral hernia repair today?

## Introduction

Treatment of abdominal wall hernias is a rapidly evolving field of surgery. Correspondingly there is a dramatic increase of publications. There are many reasons for this development: dramatic rise of the number of laparotomies and the number of major surgeries being performed, progress in anesthesiology,increase of older patients with weak connective tissue, increase of patients with risk factors for hernias, and significant increase of patients managed with an open abdomen in a damage-control situation. Worldwide as many as two million patients are operated on every year. A variety of new repair techniques came up, recently even robot-assisted operations. The surgical approach may be open, laparoscopic, endoscopically within the abdominal wall, or hybrid approaches combining these modalities. The volume of literature, often with low levels of evidence and conflicting results, can be difficult to interpret in a meaningful way to assist the surgeon in appropriate management of the hernia patient. Therefore, there is a need for evidence-based guidelines to help the surgeon in his daily decision making process. “Guidelines are the bridge between science and clinical practice (Eccles M, Mason J.Health Technol Assess. 2001; 5(16):1–69. Review.). In 2014 this same group (IEHS) published the first international “Guidelines for laparoscopic treatment of ventral and incisional abdominal wall hernias” [1–3]. It is generally accepted that guidelines require an update every three years to reflect the rapid evolution of techniques, materials and data available. The current update follows the same methodology as described in the original guidelines. The authors were encouraged to avoid redundancy and concentrate on the new studies showing a level of evidence 1, 2 and 3, and which were published between 2012 and 2017. Statements and recommendations which are still valid are not repeated. As such, this update should be read in the context and in conjunction with the initially published guidelines. New topics included in this update are: In which patient group is a component separation indicated? Should the component separation be done open or endoscopically? Is an anterior component separation better than the posterior one? Is preliminary treatment with Botox indicated in patients in whom a component separation is planned? Should TAR be done open or endoscopically? In patients presenting with a ventral hernia in combination with a rectus diastasis which is the best treatment option? Does robot- assisted surgery have a future in repair of primary and secondary ventral hernias? What is the optimal treatment of lateral primary or incisional hernias? We are well aware that with respect to these innovations the evidence is not yet strong enough to give valuable statements or recommendations, however, the guidelines should inform the surgical community and stimulate further studies to gain more knowledge in the coming years.

**References**

1. Bittner R, Bingener-Casey J, Dietz U, Fabian M, Ferzli GS, Fortelny RH, Köckerling F, Kukleta J, Leblanc K, Lomanto D, Misra MC, Bansal VK, Morales-Conde S, Ramshaw B, Reinpold W, Rim S, Rohr M, Schrittwieser R, Simon T, Smietanski M, Stechemesser B, Timoney M, Chowbey P; International Endohernia Society (IEHS) (2014) Guidelines for laparoscopic treatment of ventral and incisional abdominal wall hernias (International Endohernia Society (IEHS)-part 1. Surg Endosc Jan; 28(1):2–29. doi: 10.1007/s00464-013-3170-6. Epub 2013 Oct 11
2. Bittner R, Bingener-Casey J, Dietz U, Fabian M, Ferzli GS, Fortelny RH, Köckerling F, Kukleta J, Leblanc K, Lomanto D, Misra MC, Bansal VK, Morales-Conde S, Ramshaw B, Reinpold W, Rim S, Rohr M, Schrittwieser R, Simon T, Smietanski M, Stechemesser B, Timoney M, Chowbey P; International Endohernia Society (IEHS) (2014) Guidelines for laparoscopic treatment of ventral and incisional abdominal wall hernias (International Endohernia Society (IEHS)-part 2. Surg Endosc Feb;28(2):353–79.
3. Bittner R, Bingener-Casey J, Dietz U, Fabian M, Ferzli GS, Fortelny RH, Köckerling F, Kukleta J, Leblanc K, Lomanto D, Misra MC, Bansal VK, Morales-Conde S, Ramshaw B, Reinpold W, Rim S, Rohr M, Schrittwieser R, Simon T, Smietanski M, Stechemesser B, Timoney M, Chowbey P; International Endohernia Society (IEHS) (2014) Guidelines for laparoscopic treatment of ventral and incisional abdominal wall hernias (International Endohernia Society (IEHS)-part 3. Surg Endosc Feb;28(2):380–404. doi: 10.1007/s00464-013-3172-4. Epub 2013 Sep 17

## Chapter 1. How comparable are incisional and ventral hernias in terms of operative technique and outcomes?

### Bruce Ramshaw MD

**Acknowledgements** Uwe Klinge for review and editing of content, Jerome Berlin PhD for review and editing of content, Brandie Forman for review and clerical assistance.

**Key questions:**

1. Is the outcome of surgical treatment of primary ventral hernias different in comparison to the outcome of surgery of secondary ventral hernias?
2. In studies comparing different treatment options, does it make sense to mix primary and secondary ventral hernias in one treatment group?

Search terms (publications identified as pertinent to this topic/total publications returned by search): variability of incisional hernia (3/5), variability of ventral hernia (2/8), laparoscopic ventral hernia variability (0/0), laparoscopic incisional hernia repair variability (0/1), complexity of ventral hernia repair (2/14), complexity of laparoscopic ventral hernia repair (2/8), complexity of incisional hernia repair (0/7), complexity of laparoscopic incisional hernia repair (0/5).

The search was performed in October 2017 and a total of four unique publications were returned from this search. All four were clinical studies. A secondary search revealed additional 22 publications pertinent to this topic, ten of which were studies and twelve publications which were not clinical studies.

**Update:**For this update, additional search terms included clinical quality improvement, CQI, and quality improvement principles matched with hernia, ventral hernia, and incisional hernia.

There were no significant published manuscripts that led to a change in the statements and recommendations. (For the study of the original guidelines, read the publication in “Surg Endosc (2014) 28: page 4–7.”) The previous statements and recommendations are still valid and therefore not repeated. However, one additional statement and recommendation was included concerning the difference between a primary ventral hernia and an incisional and/or recurrent ventral hernia.

**New statement**

**Table:** 

Level 3	There are differences in outcomes when treating primary ventral hernias compared with incisional and/or recurrent ventral hernias

**New recommendation:**

**Table:** 

Grade B	When studying ventral hernias, the analysis of primary ventral hernias should be done separately from the analysis of incisional and recurrent ventral hernias

**Introduction**

What once was considered a relatively simple problem by many physicians and patients, abdominal wall hernia disease, is clearly more complex than previously thought. In addition, the patient groups presenting with incisional and ventral hernias are becoming more complex as the treatment options, including the varieties of mesh, continue to grow. This increasing complexity as well as the variability of outcomes leads us to challenge the traditional application of evidence-based medicine, which until now does not include knowledge generated from clinical quality improvement studies. This is not to say that this understanding of evidence-based medicine does not have value for complex problems, such as abdominal wall hernia disease. It is, however incomplete, and is but a starting point rather than a goal toward the understanding of how to improve the value of care for both the patient who presents with a ventral/incisional hernia and for the system in which that care is provided. In the previously published chapter (see above), the current evidence for the variability of ventral/incisional hernia patients was described and a brief framework for understanding how to apply new thinking to the study of complex problems such as ventral/incisional hernia disease is provided.

**Research:**

In our chapter published in the original guidelines it was emphasized that the knowledge of complex systems and increasing complexity impacts our understanding of the variability we see for the patient with a ventral/incisional hernia. Variability that can impact outcomes for ventral/incisional hernia repair may include patient factors, technique variability, surgeon skill, variability in mesh characteristics, and the variability in both the environmental conditions present in the patient’s home living conditions, as well as at the facility where treatment occurs. Studies on the variability of ventral/incisional hernias are few, but a comparison of studies of different types of ventral/incisional hernias clearly shows a large variety of outcomes based upon many complex factors.

One variable that currently has been studied is the type of ventral hernia, primary or incisional. Studies have consistently shown that the outcomes of these two types of ventral hernias are different, so it would be inappropriate to combine them when attempting to study ventral hernia repair (1–6). But, the science is more complex than that. It is too simple to just look at primary vs. incisional hernia. Some sub-populations of primary hernias will have more risk/worse outcomes than some sub-populations of incisional/secondary hernias. Because in the real world of complex systems science, we cannot uncouple the many factors that combine to result in many potential outcomes. We know that many factors like BMI, smoking, collagen disease, diabetes, can impact outcomes (and we will need to understand the complex interactions between factors), and we have even discovered that a patient’s cognitive/emotional state has a major impact on outcomes, but that is rarely being measured or modified/optimized preoperatively yet [7, 8]. Because of constant change and uncontrollable patient variability, learning to apply the principles of complex systems science will be essential to better understand the optimal treatment for complex disease processes, such as ventral/incisional hernia disease.

During the time between the first publication of these guidelines and this update, there have been at least six peer-reviewed publications demonstrating the use of the principles of complex systems science (using tools like clinical quality improvement) applied to hernia disease, five specifically for ventral/incisional hernia disease (8–13).

**Summary:**

In summary, the traditional human subjects’ clinical research approach to generate evidence-based medicine guidelines alone is unable to produce improved value for patient care that will be significant and sustainable for our increasingly complex healthcare system. Specifically, the increasing variability in ventral/incisional hernia patients and technique options minimizes the value of applying traditional research methods to improve outcomes. We will need to change our thinking and learn how to understand and implement research methods designed to address this increasing complexity in order to fully address healthcare challenges, such as ventral/incisional hernia disease. This will not only include an evolution of traditional/current evidence-based medicine, but also an evolution of evidence-based management in health care. Because complex systems research is most often applied in the real world of patient care in the community, hospital, clinic, and even the academic medical center, we will need to apply the principles of continuous learning and clinical quality improvement to our regular patient care in addition to using traditional clinical research methods. As we apply these new principles (new to healthcare, although currently used in other industries) and learn how to utilize complex systems science-driven data analytics (a variety of non-linear analytical tools), the patient clusters that emerge will guide our treatment options and lead to improved value for our entire system.

**References** (in parenthesis the level of evidence)

1. Kroese LF, Gillion JF, Jeekel J, Kleinrensink GJ, Lange JF (2018) Primary and incisional hernias are different in terms of patient characteristics and postoperative complications—A prospective cohort study of 4565 patients. Int J Surg. 51:114–119. (2)
2. Lambrecht JR, Vaktskjold A, Trondsen E, Oyen OM, Reiertsen O (2015) Laparoscopic ventral hernia repair: outcomes in primary versus incisional hernias: no effect of defect closure. Hernia. 19(3):479–486. (4)
3. Kurian A, Gallagher S, Cheeyandira A, Josloff R (2010) Laparoscopic repair of primary versus incisional ventral hernias: time to recognize the differences? Hernia. 14(4):383–387. (4)
4. Köckerling F, Schug-Paß C, Adolf D, Reinpold D, Stechemesser B (2015). Is pooled data analysis of ventral and incisional hernia repair acceptable? Front Surg May 12;2:15. 10.3389/fsurg.2015.00015.eCollection (2c)
5. Vincent M, Stirler A, Schoenrnaeckers EJP, de Haas RJ, Raymakers JTFJ, Rakic S (2014) Laparoscopic repair of primary and incisional ventral hernias: the differences must be acknowledged. A prospective cohort analysis of 1088 consecutive patients. Surg Endosc 28:891–895. (3)
6. Subramanian A1, Clapp ML, Hicks SC, Awad SS, Liang MK (2013) Laparoscopic ventral hernia repair: primary versus secondary hernias. J Surg Res. 2013 May 1;181(1):e1–5. 10.1016/j.jss.2012.06.028. Epub 201 (4)
7. Aspari AR, Lakshman K (2018) Effects of pre-operative psychological status on post-operative recovery: a prospective study. World J Surg. 42(1):12–18. (4)
8. Ramshaw B, Vetrano V, Jagadish M, Forman B, Heidel E, Mancini M (2017) Laparoscopic approach for the treatment of chronic groin pain after inguinal hernia repair. Surg Endosc. 31(12):5267–5274. (CQI)
9. Stephan B, Ramshaw B, Forman B (2015) Value-based clinical quality improvement (CQI) for patients undergoing abdominal wall reconstruction. Surg Technol Int. 26:135–42. (CQI)
10. Ramshaw B, Dean J, Forman B, Heidel E, Gamenthaler A, Fabian M (2016) Can abdominal wall reconstruction be safely performed without drains? Am Surg. 82(8):707–12. (CQI)
11. Ramshaw B, Forman B, Heidel E, Dean J, Gamenthaler A, Fabian M (2016) A clinical quality improvement (CQI) project to improve pain after laparoscopic ventral hernia repair. Surg Technol Int. XXIX:125–130. (CQI)
12. Ramshaw B, Forman B, Moore K, Heidel E, Fabian M, Mancini G, Joshi GP (2017) Real-world clinical quality improvement for complex abdominal wall reconstruction. Surg Technol Int. 13:30:155–164. (CQI)
13. Ganesh Kumar N, Faqih AA, Feng MP, Miller RS, Pierce RA, Sharp KW, Holzman MD, Poulose BK (2017) Using quality improvement principles to enhance long-term completion of patient-reported outcomes after ventral hernia repair. J Am Coll Surg 224(2):172–179. (CQI)

## Chapter 2a. Is the routine application of computed tomography (CT) and magnetic resonance imaging (MRI) recommended for the diagnosis of ventral hernias before laparoscopic ventral hernia repair?

### R Schrittwieser, F Mayer, H. Niebuhr

**Questions:**

1. How important are CT-Scan and MRI in preoperative diagnosis?
2. How important are CT and MRI in postoperative diagnosis?

**Search terms:**

The Pubmed search used the following search terms: “CT- scan” AND “ventral hernia” AND “laparoscopy”; “MRI” AND “ventral hernia” AND “laparoscopy.”

**Search machines:**

PubMed, Medline, and the Cochrane Library as well as the reference lists of the included studies were searched for relevant studies.

**New publications:**

A total of 3 new publications were identified since the publication of the original guidelines. Statements and recommendations were modified accordingly. For the study of the original guidelines, read the publication in Surgical Endoscopy (2014) 28: page 7–8.

**New statements preoperative**

**Table:** 

Level 4	A CT-scan can be helpful in predicting wound complications and the need for complex abdominal wall repair techniques
Level 4	Preoperative determination of abdominal wall defect ratios and hernia defect areas may be helpful to predict abdominal wall closure after Component Separation Techniques (CST)

**New recommendations preoperative:**

**Table:** 

Grade D	In bigger or incarcerated hernias, a CT-scan may be considered for better planning of op-strategy and patient information
Grade D	In planned CST, a CT-scan can be helpful to predict abdominal wall closure

**New statements postoperative:**

**Table:** 

Level 3	There is high interobserver variability in detecting a ventral hernia with CT-scan; exact definitions for a radiographic recurrence are needed

**Comments:**

• In 2016 Holihan et al. [1] published a study on the use of CT in diagnosing hernia recurrence and demonstrated astonishingly, that there was disagreement in 73 from 100 cases of CT-scans in patients with recurrence of incisional hernia between 9 blinded reviewers. The authors concluded that the concepts most frequently discussed were the absence of an accepted definition for a radiographic ventral hernia and differentiating pseudorecurrence from recurrence. Another topic that has gained importance over the last years was the use of component separation techniques. Franklin et al. [2] investigated the role of CT-scan in predicting abdominal wall closure after CST. Performing a retrospective study on 54 patients the authors concluded, that preoperative determination of abdominal wall defect ratios and hernia defect areas may represent a more accurate method to predict abdominal wall closure after CST. Blair et al. [3] investigated 151 cases of open ventral hernia repair. They measured the hernia defects and abdominal wall thickness to predict wound complications and the need for complex abdominal wall repair techniques. One of the conclusions was that obtaining preoperative CT imaging should be a consideration in preoperative planning and may help with patient counseling. An interesting study was done by G. Köhler et al. [4] who operated 10 patients in laparoscopic IPOM technique with MRI-visible Meshes. The authors could demonstrate mesh shrinkage by a significant decrease of the mesh surface area within the first 3 months by routine MRI in a limited number of comparable cases.

**References** (in parenthesis the level of evidence)

1. Holihan JL, Karanjawala B, Ko A, Askenasy EP, Matta EJ, Gharbaoui L, Hasapes JP, Tammisetti VS, Thupili CR, Alawadi ZM, Bondre I, Flores-Gonzalez JR, Kao LS, Liang MK (2016) Use of Computed Tomography in Diagnosing Ventral Hernia Recurrence: A Blinded, Prospective, Multispecialty Evaluation. JAMA Surg. 151(1):7–13. (1B)
2. Franklin BR, Patel KM, Nahabedian MY, Baldassari LE, Cohen EI, Bhanot P (2013) Predicting abdominal closure after component separation for complex ventral hernias: maximizing the use of preoperative computed tomography. AnnPlast Surg. 71(3):261–265 (4)
3. Blair LJ, Ross SW, Huntington CR, Watkins JD, Prasad T, Lincourt AE, Augenstein VA, Heniford BT (2015) Computed tomographic measurements predict component separation in ventral hernia repair. J Surg Res 2015 Dec;199(2):420–427 (2B)
4. Köhler G, Pallwein-Prettner L, Koch O, Luketina R, Lechner M, Emmanuel K (2015) Magnetic resonance-visible meshes for laparoscopic ventral hernia repair. JSLS 19(1): e2014.00175 (4)

## Chapter 2b. Key question: Can the routine application of ultrasound imaging be helpful in detecting ventral hernias and rectus diastasis preoperatively?

### H Niebuhr, R. Schrittwieser

The Pubmed search used the following search terms: “Ultrasound” AND “ventral hernia”; “Ultrasound” AND “rectus diastasis.” The search was performed in January 2018. The search detected 12 articles (7 for ultrasound and ventral hernia; 5 for ultrasound and rectus diastasis).

**Statements abdominal wall hernia:**

**Table:** 

Level 4	The evidence for the use of US in the daily routine is insufficient
	High-frequency US can be helpful in depicting/diagnosing epigastric abdominal wall hernias and incisional hernias of limited size
	The Field of view (FOV) can be extended by using panoramic ultrasound view
	Further information can be gained by using shear wave elastography (SWE)

**Recommendations:**

**Table:** 

Grade C	The reliability of shear wave elastography (SWE) in diagnosis of abdominal wall hernia disease should be further evaluated

**Literature search Abdominal wall hernia:**

5 case reports were identified [1, 3, 4, 6, 7] regarding:

- an incarcerated epigastric hernia in an elderly patient,
- a clinical manifestation of a tumor formation on the abdominal wall: differential diagnosis,
- a chronic infective osteomyelitis of the xiphoid process of the sternum (DD Abdominal wall hernia) in a young woman,
- an incarcerated small bowel in the hernia with no flow in the mesentery in a 90-year-old man,
- an emergent case of a Spigelian hernia involving the appendix.

All reports highlight the importance of sonography, both as a diagnostic and interventional modality to obtain the correct diagnosis in unclear abdominal wall lump, tumor, or mass.

Two feasibility studies [2, 5] identify ultrasound examination as a non-invasive diagnostic tool that allows the differentiation of hernia from other abdominal swellings [2]. The feasibility of US SWE (Ultrasound combined with shear wave elastography) to detect ventral hernias and evaluate mesh repair in vivo could be demonstrated [5].

The results indicate that the presence of a hernia and repair can be reliably visualized by SWE and three-dimensional reconstruction. This technique may provide both structural and functional information regarding the hernia and the repair.

**References** (in parenthesis the level of evidence)

1. Suarez Acosta CE, Romero Fenandez E, Calvo Manuel E (2015) Epigastric Hernia Indian J Surg. Aug; 77(4): 335. Published online 2015 Mar 23. 10.1007/s1226201512859 (4)
2. Amer MS, Hassan EA, Torad FA (2018) Radiographic and ultrasonographic characteristics of ventral abdominal hernia in pigeons (*Columba livia*). J Vet Med Sci 80(2):292–296. 10.1292/jvms.17-0517. Epub 2017; Dec 14 (5)
3. Tchernev G, Chokoeva A, Lotti J, Franca K, Lotti T (2017) Ventral Abdominal Hernia Open Access Maced J Med Sci 5(5): 694–695. Published online 2017 Aug 10. 10.3889/oamjms.2017.154 (4)
4. Bhandari Grover S, Aurora S, Kumar A, Grover H, Katyan A, Mair DM (2017) “Caught by the Eye of Sound” – Epigastric Swelling due to Xiphisternal tuberculosis. Pol J Radiol 82: 41–45. Published online 2017; Jan 27. 10.12659/pjr.899329 (4)
5. Chaudhry A, Fernandez-Moure JS, Shajudeen PS, Van Eps JL, Cabrera FJ, Weiner BK, Dunkin BJ, Tasciotti E, Righetti R (2017) Characterization of ventral incisional hernia and repair using shear wave elastography. J Surg Res 210: 244–252. 10.1016/j.jss.2016.11.041. Epub 2016; Nov 30 (5)
6. Abu-Zidan FM, Idris K, Khalifa M (2016) Strangulated epigastric hernia in a 90-year-old man: Point-of-Care. Ultrasound (POCUS) as a saving kit: Case report. Int J Surg Case Rep 22: 19–22. Published online 2016; Mar18. 10.1016/j.ijscr.2016.03.016 (4)
7. Xu L, Dulku G, Ho R (2017) A rare presentation of Spigelian hernia involving the appendix. Eur J Radiol Open 4: 141–143. Published online 2017 Nov 9. 10.1016/j.ejro.2017.11.002 (4)

**Statements rectus diastasis:**

**Table:** 

Level 4	High-frequency US can be helpful in depicting/diagnosing/measuring of a rectus diastasis
	The Field of view (FOV) can be extended by using panoramic ultrasound view

**Recommendations:**

**Table:** 

Grade A	High-frequency US is recommended to depict/diagnose/measure a rectus diastasis
	Panoramic ultrasound view can be used to extend the Field of view (FOV)

**Literature search rectus diastasis:**

A Systematic review and meta-analysis revealed thirteen studies to evaluate measurement properties of the ‘finger-width’ method, tape measure, calipers, ultrasound, CT, and MRI. Ultrasound was most evaluated. Methodological quality of these studies varied widely. The available information supports ultrasound and calipers as adequate methods to assess DRAM (Diastasis of Rectus Abdominal Muscle). For other methods, limited measurement information of low-to-moderate quality is available [1].

A longitudinal descriptive exploratory study evaluates the normal width of the linea alba in first-time pregnant women during pregnancy and postpartum. Different normative values for the width of the linea alba were found at different locations of the anterior abdominal wall. In primiparous women, the IRD may be considered “normal” up to values wider than in nulliparous [2].

A study describes the relationship between inter-rectus distance (IRD) and symptom severity. IRD was significantly correlated with worst abdominal pain in the last 24 h (*ρ* = 0.45, *p* = 0.005), and with overall body image (*ρ* = − 0.44, *p* = 0.006), but not with the other outcomes [3].

In an intra-rater between-session reliability study, the between-session reliability of IRD measurement was high, particularly when measuring IRD at or above the umbilicus. When performed by an experienced investigator, ultrasound imaging is a reliable tool to measure IRD in postpartum women who have diastasis recti [4].

Another Reliability and validity study promotes the value of extended field of view (FOV) technique: Ultrasound imaging is the gold standard for non-invasive IRD measurement in parous women when investigating diastasis recti; however, its use is limited when IRD is large. Extended FOV techniques (panoramic USI or using acoustic standoff pads) allow complete visualization of the linea alba when the IRD is large and conventional imaging is not sufficient. FOV techniques were highly correlated with those acquired using conventional imaging (*r* > 0.95, *p* < 0.0001) [5].

**References** rectus diastasis (in parenthesis the level of evidence)

1. Van de Water AT, Benjamin DR (2016) Measurement methods to assess diastasis of the rectus abdominis muscle (DRAM): A systematic review of their measurement properties and meta-analytic reliability generalisation. Man Ther 21:41–53. 10.1016/j.math.2015.09.013. Epub 2015; Oct 3. (1B)
2. Mota P, Pascoal AG, Carita AI, Bø K (2018) Normal width of the inter-recti distance in pregnant and postpartum primiparous women. Musculoskelet Sci Pract 35:34–37. 10.1016/j.msksp.2018.02.004. [Epub ahead of print] (3)
3. Keshwani N, Mathur S, McLean L (2018) Relationship Between Inter-rectus Distance and Symptom Severity in Women With Diastasis Recti in the Early Postpartum Period. Phys Ther 98(3):182–190. 10.1093/ptj/pzx117. [Epub ahead of print]
4. Keshwani N, Mc Lean L (2015) Postpartum Women With Diastasis Recti: Intrarater Between-Session Reliability. J Orthop Sports Phys Ther 45(9):713–718. 10.2519/jospt.2015.5879. Epub 2015 Jul 10. (3)
5. Keshwani N, Mathur S, McLean L (2015) Validity of Inter-rectus Distance Measurement in Postpartum Women Using Extended Field-of-View Ultrasound Imaging Techniques. J Orthop Sports Phys Ther 45(10):808–13. 10.2519/jospt.2015.6143. Epub 2015 Aug 24. (3)

## Chapter 3. Classification

### U.A. Dietz, A. Wiegering

In analyzing the literature since the first version of the IEHS Guidelines 2013, three following conclusions can be reached: a) the importance of classification is widely acknowledged in the literature, but considering the great majority of published data, classification criteria (besides the size of the hernia) have not yet reached prominent influence on treatment algorithms; b) in retrospective studies the classification of hernias seems to be established; and c) the available classifications need to be further developed and refined, since data analysis from registries and quality control databases will increase in importance.

The most widely used classification is the European Hernia Society (EHS) classification [1]. Kroese et al. (2018) [2], in a conjoint research from Erasmus Medical Centre and the French Club-Hernia, investigated the EHS classification as a predictor for postoperative complications after incisional hernia surgery, using a registry-based prospective collected database (*n* = 2191 patients). Fifteen percent of patients had at least one complication; EHS width class, incarceration, open surgery, duration of surgery, Altemeier wound class, and therapeutic antibiotic treatment were independent risk factors for postoperative complications. The authors concluded that the EHS classification is useful to identify patients at risk for complications. This study is an external validation of the EHS classification. Actual data regarding recurrence show that ventral and incisional hernias are distinctive entities, as already defined in the previous classifications [3].

The prospective study of Dietz et al. (2017) analyzed in a prospective and consecutive cohort of 486 patients if preoperative classification of the incisional hernia and the stratification of patients at risk for postoperative complications are helpful in a patient’s treatment algorithm including either retromuscular mesh repair, open IPOM, or laparoscopic IPOM [4, 5]. The aim was to submit each patient to a tailored procedure, in order to balance between abdominal wall reconstruction (large procedure with higher rate of complications) and symptomatic therapy (IPOM), avoiding higher risks of complications. Hernial gap width was an independent factor for the occurrence of postoperative complications (*p* = 0.002). The classification criteria applied were internally validated. The heuristic algorithm ensured that patients at high risk of complications did not have a higher perioperative complication rate than patients at low risk [4]. In a previous study [6], the same authors presented the internal validation of the Dietz classification. The criterion “recurrence rating” was found as predictive factor for postoperative complications in the multivariate analysis (OR 2.04; 95% CI 1.09–3.84; incisional vs. ventral hernia). The criterion “morphology” had influence neither on the incidence of the critical event “recurrence during follow-up” nor on the incidence of postoperative complications. Hernial gap “width” predicted postoperative complications in the multivariate analysis (OR 1.98; 95% CI 1.19–3.29; ≤ 5 vs. > 5 cm). Length of the hernial gap was found to be an independent prognostic factor for the critical event “recurrence during follow-up” (HR 2.05; 95% CI 1.25–3.37; ≤ 5 vs. > 5 cm). The presence of 3 or more risk factors was a consistent predictor for “recurrence during follow-up” (HR 2.25; 95% CI 1.28–9.92) [6].

Baucom et al. [7] analyzed in a retrospective study the prognostic differences between EHS classification medial and lateral incisional hernias regarding surgical site occurrence (SSO). The authors concluded that the rate of SSO by location (morphology) was 39% (*n* = 183) for midline, 23% (*n* = 11) for lateral, and 74% (*n* = 17) for hernias with midline and lateral components (*p* = <0.001). Patients whose midline hernia spanned more than one EHS category also had a higher rate of SSOs (*p* = 0.001). If hernia localization is an independent risk factor, SSO needs to be further evaluated in a respectively powered prospective study, since morphology was not found to be a risk factor in another study [6]. Nevertheless, the localization of the hernia (morphology) is important in planning the procedure, as Raakow et al. also showed regarding subxiphoideal incisional hernias [8].

While the EHS classification seems to be known and accepted by the surgeons, its impact on tailoring procedures has still to be demonstrated. Almost 10 years after publication of the EHS classification, there is a paradoxical gap in several studies between the use of the classification and its clinical impact on patients’ treatment: the classification is used to describe demography of the population but has also not been used to tailor the surgical procedure [9, 10]. Other casuistic classifications have not been used [examples: 11, 12, 13, 14]. Recently there was described an approach to stage incisional hernias for tailoring treatment: Stage I (< 10 cm/clean and associated with low SSO and recurrence risk), stage II (10–20 cm/clean or < 10 cm contaminated and carry an intermediate risk of SSO and recurrence), or stage III (≥ 10/contaminated or any hernia ≥ 20 cm, and these are associated with high SSO and recurrence risk) [15]. The future will show if a staging system will be more accepted by the surgical community as classification tools in tailoring procedures.

Finally, in addition to the EHS classification of ventral and incisional hernias, the EHS proposed a classification for parastomal hernias (PH), taking into account, that frequently parastomal hernia patients have concomitant incisional hernias (cIH): type I (small PH without cIH), type II (small PH with cIH), type III (large PH without cIH), and type IV (large PH with cIH); in addition, the classification grid includes details about whether the hernia recurs after a previous PH repair or whether it is a primary PH. This classification still needs to be validated [16]. The further refinement and implementation of an universally adopted ventral and incisional hernia classification will be of utmost importance, since individualized patient procedures will become more and more important. Last but not the least, register-based outcomes will in future also need to rely on validated classification criteria [17].

**Statements**

**Table:** 

Level 2B	The EHS ventral and incisional hernia classification is validated (external validation)
	The EHS classification is useful for identifying patients at risk for complications
	The classification of Dietz et al. is validated (internal validation)
	Hernia gap width is of prognostic relevance regarding postoperative complications (SSO)
	Hernia gap length is of prognostic importance regarding recurrence rate
	Ventral and incisional hernias are distinct entities with different prognosis
Level 5	A consensus exists among experts that it is necessary to classify ventral and incisional hernias as well as parastomal hernias prospectively, to create a useful data set to improve understanding of the disease, to allow comparability of results, to substantiate patient counseling, and to optimize therapeutic algorithms
	The acceptance and application of the available classifications remained low in the period from 2013 to 2018

**Recommendation**

**Table:** 

Grade D	The European Hernia Society (EHS) classification for ventral and incisional hernias is recommended

**References** (in parenthesis the level of evidence)

1. Muysoms FE, Miserez M, Berrevoet F, Campanelli G, Champault GG, Chelala E, Dietz UA, Eker HH, El Nakadi I, Hauters P, Hidalgo Pascual M, Hoeferlin A, Klinge U, Montgomery A, Simmermacher RK, Simons MP, Smietański M, Sommeling C, Tollens T, Vierendeels T, Kingsnorth A (2009). Classification of primary and incisional abdominal wall hernias. Hernia 13:407–414. (5)
2. Kroese LF, Kleinrensink GJ, Lange JF, Gillion JF and Hernia-Club (2018) External Validation of the European Hernia Society Classification for Postoperative Complications after Incisional Hernia Repair: A Cohort Study of 2191 Patients. J Am Coll Surg 226:223–229.e1. (2C)
3. Stirler VM, Schoenmaeckers EJ, de Haas RJ, Raymakers JT, Rakic S (2014) Laparoscopic repair of primary and incisional ventral hernias: the differences must be acknowledged: a prospective cohort analysis of 1088 consecutive patients. Surg Endosc 28:891–5. (2C)
4. Dietz UA, Fleischhacker A, Menzel S, Klinge U, Jurowich C, Haas K, Heuschmann P, Germer CT, Wiegering A (2017) Risk-adjusted procedure tailoring leads to uniformly low complication rates in ventral and incisional hernia repair: a propensity score analysis and internal validation of classification criteria. Hernia 21:569–582. (2B)
5. Dietz UA, Hamelmann W, Winkler MS, Debus ES, Malafaia O, Czeczko NG, Thiede A, Kuhfuss I (2007) An alternative classification of incisional hernias enlisting morphology, body type and risk factors in the assessment of prognosis and tailoring of surgical technique. J Plast Reconstr Aesthet Surg 60:383–388. (5)
6. Dietz UA, Winkler MS, Härtel RW, Fleischhacker A, Wiegering A, Isbert C, Jurowich Ch, Heuschmann P, Germer CT (2014) Importance of recurrence rating, morphology, hernial gap size, and risk factors in ventral and incisional hernia classification. Hernia 18:19–30. (2C)
7. Baucom RB, Ousley JM, Oyefule OO, Stewart MK, Holzman MD, Sharp KW, Poulose BK (2015) Incisional Hernia Classification Predicts Wound Complications Two Years after Repair. Am Surg 81:679–686. (3)
8. Raakow J, Schulte-Mäter J, Callister Y, Aydin M, Denecke C, Pratschke J, Kilian M (2018) A comparison of laparoscopic and open repair of subxiphoid incisional hernias. Hernia 10.1007/s10029-018-1815-z. [Epub ahead of print] (3)
9. García-Ureña MÁ, López-Monclús J, Cuccurullo D, Blázquez Hernando LA, García-Pastor P, Reggio S, Jiménez Cubedo E, San Miguel Méndez C, Cruz Cidoncha A, Robin Valle de Lersundi A (2018) Abdominal Wall Reconstruction Utilizing the Combination of Absorbable and Permanent Mesh in a Retromuscular Position: A Multicenter Prospective Study. World J Surg 21. 10.1007/s00268-018-4765-9. [Epub ahead of print] (2C)
10. Torregrosa-Gallud A, Sancho Muriel J, Bueno-Lledó J, García Pastor P, Iserte-Hernandez J, Bonafé-Diana S, Carreño-Sáenz O, Carbonell-Tatay F (2017) Modified components separation technique: experience treating large, complex ventral hernias at a University Hospital. Hernia 21:601–608. (3)
11. Pechman DM, Cao L, Fong C, Thodiyil P, Surick B (2018) Laparoscopic versus open emergent ventral hernia repair: utilization and outcomes analysis using the ACSNSQIP database. Surg Endosc 32(12):4999–5005. 10.1007/s00464-018-6312-z. [Epub ahead of print]. (2C)
12. Oviedo R, Robertson JC, Desai AS (2017) Robotic Ventral Hernia Repair and Endoscopic Component Separation: Outcomes. JSLS 21(3). pii: e2017.00055. 10.4293/jsls.2017.00055. (4)
13. Kokotovic D, Sjølander H, Gögenur I, Helgstrand F (2017) Correlation between early surgical complications and readmission rate after ventral hernia repair. Hernia 21:563–568. (4)
14. Savitch SL, Shah PC (2016) Closing the gap between the laparoscopic and open approaches to abdominal wall hernia repair: a trend and outcomes analysis of the ACS-NSQIP database. Surg Endosc 30:3267–3278. (2C)
15. Petro CC, O’Rourke CP, Posielski NM, Criss CN, Raigani S, Prabhu AS, Rosen MJ (2016) Designing a ventral hernia staging system. Hernia 20:111–117. (4)
16. Śmietański M, Szczepkowski M, Alexandre JA, Berger D, Bury K, Conze J, Hansson B, Janes A, Miserez M, Mandala V, Montgomery A, Morales Conde S, Muysoms F (2014) European Hernia Society classification of parastomal hernias. Hernia 18:1–6. (5)
17. Schwab R, Dietz UA, Menzel S, Wiegering A (2018) Pitfalls in interpretation of large registry data on hernia repair. Hernia 22(6):947–950. 10.1007/s10029-018-1837-6. [Epub ahead of print]. (5)

## Chapter 4: Indications for treatment dependence on size of defect or hernia sac, hernia type, symptoms, and age

### Zhenling Ji, Junsheng Li and G. Woeste

**Key questions:**

1. Is a “watchful waiting” strategy in the therapeutic concept of ventral hernias justified?
2. Is there a relationship between morbidity and size of the hernia defect?
3. Is there an age limit for ventral hernia repair?

**Search terms**

The following search terms were used: “watchful waiting”; “ventral hernia”; “umbilical hernia”; “incisional hernia”; “randomized controlled trial”; “controlled clinical trial.”

**Search machines**

Medline, PubMed, Cochrane Library, and relevant journals from July 2012 to September 2017 were used. For the study of the original guidelines, read the publication in “Surg Endosc (2014) 28: page 10–12.”

**New publications**

A total of 11 new studies were identified since the publication of the original guidelines. Statements and recommendations were modified accordingly. For the study of the original guidelines, read the publication in “Surg Endosc (2014) 28: page 10–12.”

**New statements**

**Table:** 

Level 2	Elective surgery improves hernia-related QoL and functional status (low- and moderate-risk patients), while emergency repair leads to higher morbidity and mortality
Level 2	Small hernia defects predict emergency repair (umbilical hernia defects between 2 and 7 cm and incisional hernia defects up to 7 cm) The size of the defect was an independent predictor for recurrence and postoperative complications
Level 3	Watchful waiting is safe for incisional and umbilical hernias, but it leads to high crossover rates (11–33%) with significantly greater incidence of intraoperative perforations, fistulas, and mortality for emergency surgery
Level 3	Older incisional hernia patients tend to have poor outcomes after incisional hernia repair

**New Recommendations**

**Table:** 

Grade B	Watchful waiting is suggested for medical optimization in patients with modifiable risk factors
Grade B	It is recommended that symptomatic hernias should be treated surgically. The laparoscopic technique should preferably be reserved for defect sizes smaller than 15 cm in diameter

**Comments**

There were conflicting data regarding the role of watchful waiting in ventral/incisional hernia treatment. Three studies reported that WW was a safe option [1–3], while a retrospective study reported higher crossover rate (33%) and higher unexpected intraoperative intestinal perforation rate in the crossover group (13%) compared with the operation treatment group (2%; *p* = 0.002) [4]. Another prospective study also reported that only repaired patients had improved functional scores on 6-month follow-up. In addition, a non-operative management was reported to be strongly associated with lower function scores (log odds ratio   = − 26.5; 95% confidence interval  = − 35.0 to − 18.0) [5]

**Emergency hernia**

A prospective nationwide study reported that the emergency rate accounted to 8.5% [6]. There were significantly more patients with concomitant bowel resection after emergency repairs than after elective repairs (*p* < 0.001). Furthermore, emergency umbilical/epigastric or incisional hernia repair was associated with up to 15-fold higher mortality, reoperation, and readmission rates than elective repair. Patients who underwent repair for incarceration were more likely to have strangulation, pain, and bowel obstruction than patients who had repair without incarceration or patients treated non-operatively (*p* < 0.02 each) [7]. Similarly, a prospective study also reported a complication rate of 21.3% in emergency repair [8].

**Age**

Old age was an important risk factor for emergency repair in both ventral/incisional hernias and umbilical hernias. One study demonstrates that older patients are more likely not aware that they have an incisional hernia [3]. Furthermore, advanced age was also a significant independent risk factor for poor early outcomes (readmission, reoperation, or death within 30 days) (*p* < 0.05) [3, 7].

**Indication related to size**

From a randomized clinical trial comparing laparoscopic vs open incisional hernia repair with 206 patients from 10 hospitals [9], the authors found that the defect size was an independent predictor for recurrence (*p* < 0.001) [9]. In a prospective nationwide study [3], they also found that defect size was a risk factor for emergency repair. Furthermore, older age, female gender, and umbilical hernia defects between 2 and 7 cm or incisional hernia defects up to 7 cm were important risk factors for emergency repair. A retrospective study also revealed that there was a correlation between the hernia gap size and risk factors [10], while another trial comparing laparoscopic and open mesh repair found that hernia size did not influence the surgical outcomes [11].

**References** (in parentheses the level of evidence)

1. Kokotovic D, Sjølander H, Gögenur I, Helgstrand F (2016) Watchful waiting as a treatment strategy for patients with a ventral hernia appears to be safe. Hernia 20:281–7 (2C)
2. Bellows CF, Robinson C, Fitzgibbons RJ, et al. (2014) Watchful waiting for ventral hernias: a longitudinal study. Am Surg 80:245–252 (3)
3. Ah-Kee EY, Kallachil T, O’Dwyer PJ. (2014) Patient awareness and symptoms from an incisional hernia. Int Surg 99:241–246 (3)
4. Verhelst J, Timmermans L, van de Velde M, Jairam A, Vakalopoulos KA, Jeekel J, Lange JF (2015) Watchful waiting in incisional hernia: is it safe? Surgery 157:297–303 (3)
5. Holihan JL, Henchcliffe BE, Mo J, et al. (2016) Is Nonoperative Management Warranted in Ventral Hernia Patients with Comorbidities?: A Case-matched, Prospective, Patient-centered Study. Ann Surg 264:585–590 (2)
6. Helgstrand F, Rosenberg J, Kehlet H, Bisgaard T (2013) Outcomes after emergency versus elective ventral hernia repair: a prospective nationwide study. World J Surg 37:2273–9 (2C)
7. Stey AM, Danzig M, Qiu S, Yin S, Divino CM (2014) Cost-utility analysis of repair of reducible ventral hernia. Surgery 155:1081–9 (3)
8. Bessa SS, Abdel-Razek AH (2013) Results of prosthetic mesh repair in the emergency management of the acutely incarcerated and/or strangulated ventral hernias: a seven years study. Hernia 17:59–65 (2A)
9. Eker HH, Hansson BM, Buunen M, Janssen IM, Pierik RE, Hop WC, Bonjer HJ, Jeekel J, Lange JF (2013) Laparoscopic vs. open incisional hernia repair: a randomized clinical trial. JAMA Surg 148:259–63 (3)
10. Dietz UA, Winkler MS, Härtel RW, et al. (2014) Importance of recurrence rating, morphology, hernial gap size, and risk factors in ventral and incisional hernia classification. Hernia 18:19–30 (2)
11. Rogmark P, Petersson U, Bringman S, Ezra E, Österberg J, Montgomery A (2016) Quality of Life and Surgical Outcome 1 Year After Open and Laparoscopic Incisional Hernia Repair: PROLOVE: A Randomized Controlled Trial. Ann Surg 263:244–50 (3)

## Chapter 5. Is there still a place for open suture repair depending on defect size?

### J. Kukleta, S. Morales-Conde

**Question:**

Up to which defect size suture repair may be justified or should all ventral hernias repaired by a mesh?

**Literature search**

Search terms were “small hernia” AND “non-mesh repair” AND “suture repair” AND “recurrence” AND “infection,” “umbilical hernia,” “epigastric hernia,” AND mesh repair, “incisional hernia” AND “ventral hernia.”

A systematic search was performed in September 2017 using Pubmed, Medline, and reference lists from January 2001 to September 2017 in order to pick up eventually missed articles in the previous search in 2012. Additional separate search was done in Pubmed, Springer link, BJS, and reference lists manually from 2012 till September 2017 using the filters for higher level of evidence.

Of the 5566 titles screened for small hernia (4764 for umbilical hernia and 716 for epigastric hernia), 646 for small hernia (179 for umbilical and 68 for epigastric) were checked. After adding Meta-Analysis, Randomized controlled trial, 5 years, and systematic review as filter 78 articles met the search criteria. Three Meta-analyses and nine RCTs are relevant for this review.

After thorough analysis of previous statements and recommendations (“Surg Endosc (2014) 28: page 12–14”), previous relevant literature and recently published articles of higher level of evidence the statements and recommendations remain valid.

Still several aspects of reporting have changed. The focus on the outcomes of the repair of small hernias (umbilical, epigastric, trocar hernias) has additionally widened from recurrence and infection to, e.g., readmission rate, acute and chronic pain, comparison of different devices, different approaches, or different positions of prosthetic material within the abdominal wall. The existing literature contains several new statements and recommendations.

**Statements**

**Table:** 

Level 1A	A mesh repair reduces the number of recurrences significantly. The suture repair is associated with more recurrent hernias than mesh repair
Level 2	Sublay mesh location may result in fewer recurrences and SSIs than onlay or inlay placement
Level 3	Surgical site infections and seromas are more common with mesh repair
	Rectus diastasis (divarication recti) is a significant risk factor for increased recurrence rate in repair of small midline hernias

**Recommendations**

**Table:** 

Grade A	Mesh reinforcement is recommended for all VH repairs (diameter > 1 cm) in a clean case
Grade B	Mesh reinforcement is suggested in even small umbilical or epigastric hernias (diameter > 1 cm) to lower the risk of reoperation for recurrence
	It is suggested that patients with small midline hernias and concomitant divarication recti should receive a mesh repair to decrease the risk of recurrence

The meta-analysis of Mathes [1] comments risk of bias of the ten included RCTs to be moderate to high (Table 1). The suture repair was in all comparisons associated with more recurrent hernias than mesh repair.

Table 1 Mathes T et al. World J Surg (2016) 40:826–835

**Figure:**
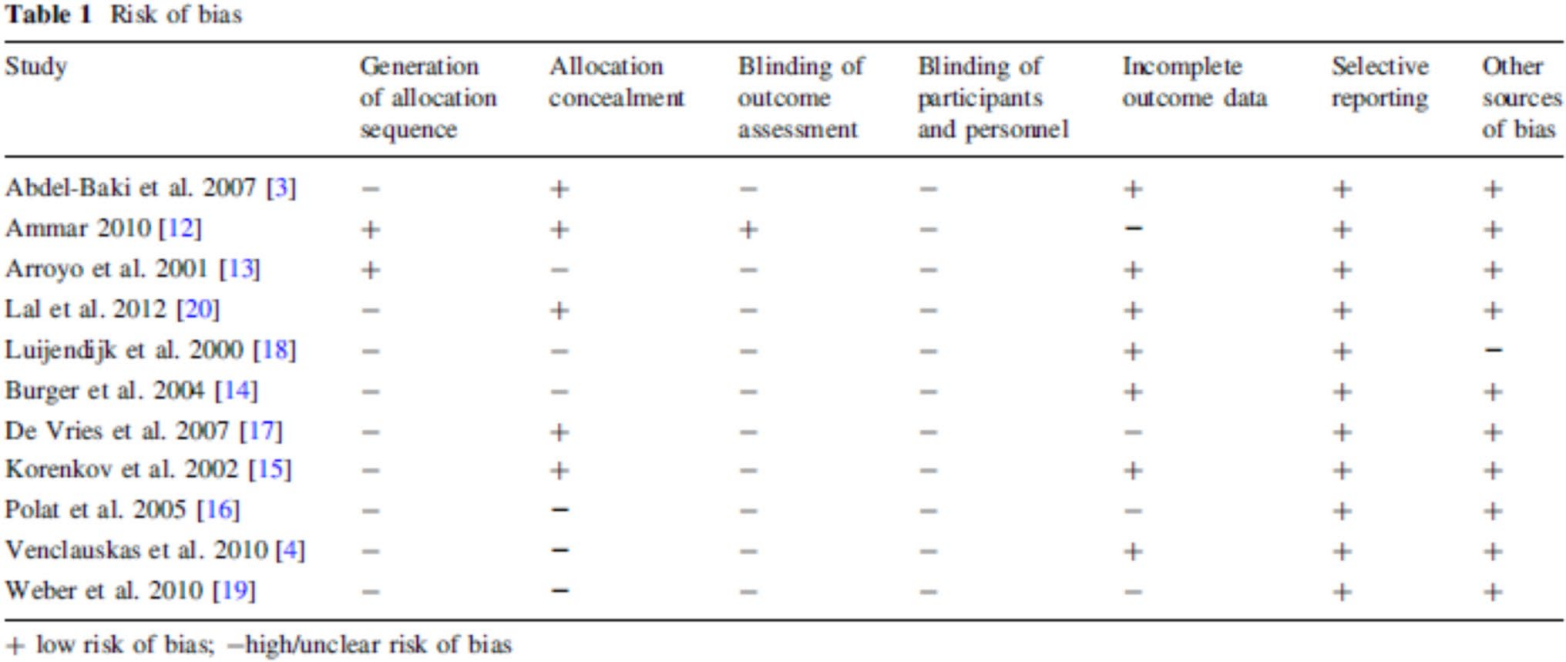

The suture repair was in all studies associated with more recurrent hernias than mesh repair. The RR for recurrence was 0.36 [95% CI (0.27, 0.49)] in favor of mesh repair. This difference was highly statistically significant (*p* < 0.00001). Nine of ten comparisons were statistically significant (Table 2).

**Table 2** Long-term complications, results. From Mathes T et al. World J Surg (2016) 40:826–835

**Figure:**
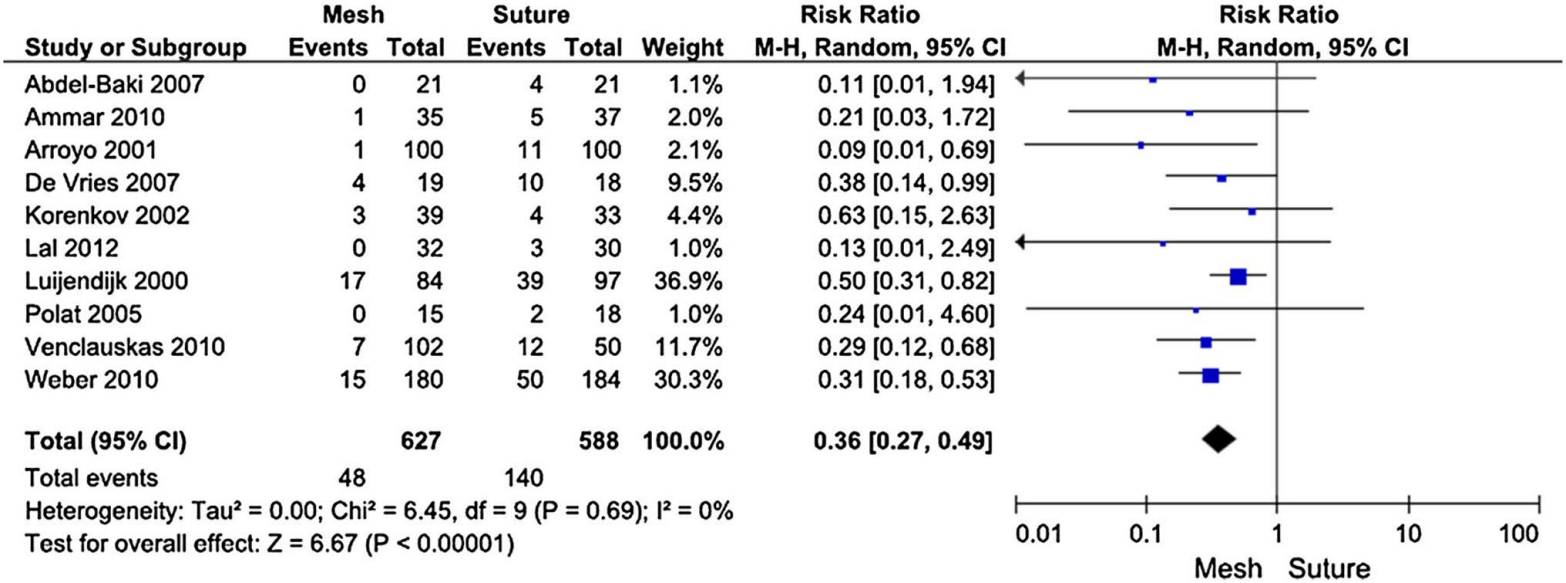

In the meta-analysis, mesh repair reduces the number of recurrences significantly. However, in patients without recurrence, mesh repair seems to be associated with a risk of chronic pain especially if the mesh is fixed sublay. This meta-analysis is based on best available evidence (RCTs) and is therefore a good basis for recommendations (level of evidence 1a) in surgical evidence-based clinical practice guidelines.

The meta-analysis of Holihan [2] includes 23 articles on quantitative synthesis and individually includes or excludes the papers in relation to three key questions. Key question 1: When is mesh reinforcement indicated during VH repair? Key question 2: What type of mesh is recommended for VH repair? And Key question 3: Where should mesh be placed during open VH repair? Evidence indicates that mesh reinforcement in clean cases can decrease hernia recurrence (number needed to treat = 7.9) but increase the risk of SSI (number needed to harm = 27.8). Placing mesh in the sublay position (as opposed to the onlay or underlay position) may decrease the risk of hernia recurrence and SSI. Holihan et al. conclude that mesh reinforcement is recommended for all VH repairs in a clean case (high grade of evidence). Sublay mesh location may result in fewer recurrences and SSIs than onlay or inlay placement (moderate grade of evidence).

The systematic review and meta-analysis of Nguyen [3] analyzes 9 studies with 637 mesh repairs and 1145 suture repairs. The pooled mesh repairs demonstrated a 2.7% recurrence rate, 7.7% seroma rate, and 7.3% SSI rate. The pooled suture repairs demonstrated an 8.2% recurrence rate, 3.8% seroma rate, and 6.6% SSI rate. Based on the multivariate meta-analysis, recurrence is more common with suture repair. SSIs and seromas are more common with mesh repair.

Kaufman et al. [4] published recently a randomized, double-blind, controlled, multicenter trial comparing mesh repair and suture repair of umbilical hernias in adults. It shows high level of evidence for mesh repair in patients with small hernias of diameter 1–4 cm. After a follow-up of 30 months, the recurrence rate was 4% for mesh and 12% for suture repair. The authors suggest mesh repair should be used for operations on all patients with an umbilical hernia of this size.

Lower Reoperation Rate for Recurrence after Mesh versus Sutured Elective Repair in Small Umbilical and Epigastric Hernias. A Nationwide Register Study is the title of the Christoffersen’s paper from 2013 [7]. In total, 4786 small (equal or smaller than 2 cm) elective open umbilical and epigastric hernia repairs were included. Follow-up was 21 months (range 0–47 months). The cumulated reoperation rates for recurrence were 2.2% for mesh reinforcement and 5.6% for sutured repair (*p* = 0.001) (Fig. 1a). The types of hernia repairs were divided into two groups: mesh repair (inlay/plug, sublay, onlay, and intraperitoneal) and sutured repair [fast absorbable sutures (e.g., polyglactin), slowly absorbable suture (e.g., polydioxanone), and non-absorbable suture (e.g., polypropylene)] (Fig. 1b).

**Fig. 1:**
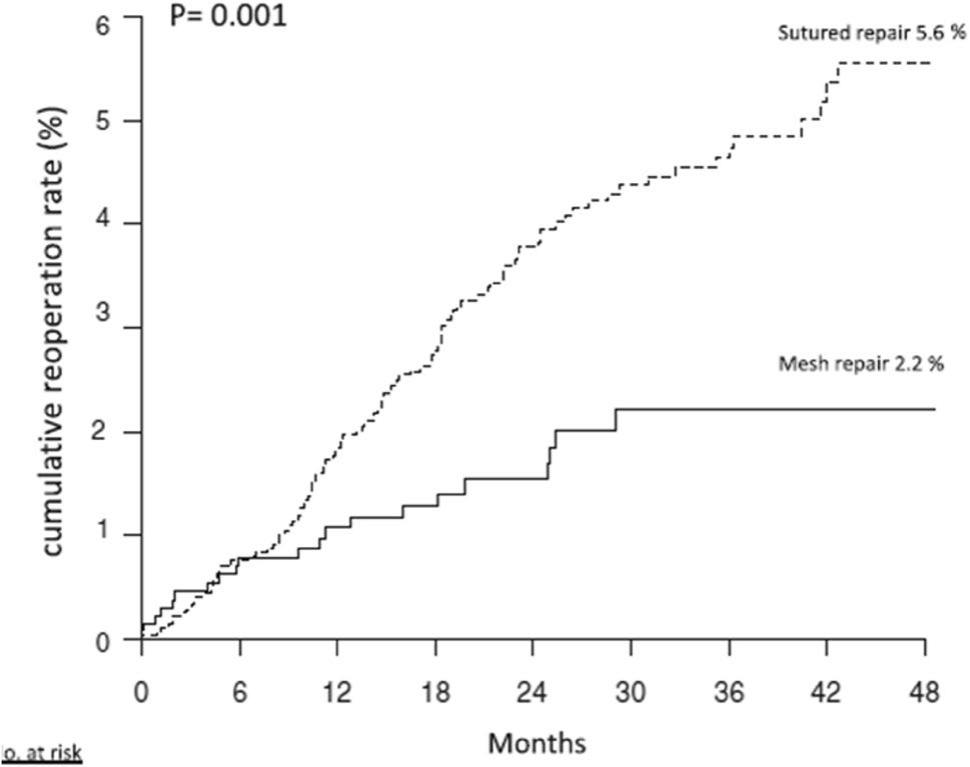
**A** Kaplan–Meier plot illustrating the cumulated risk of reoperation for recurrences for mesh versus sutured repair (log rank, *p* = 0.001)

**Fig. 1:**
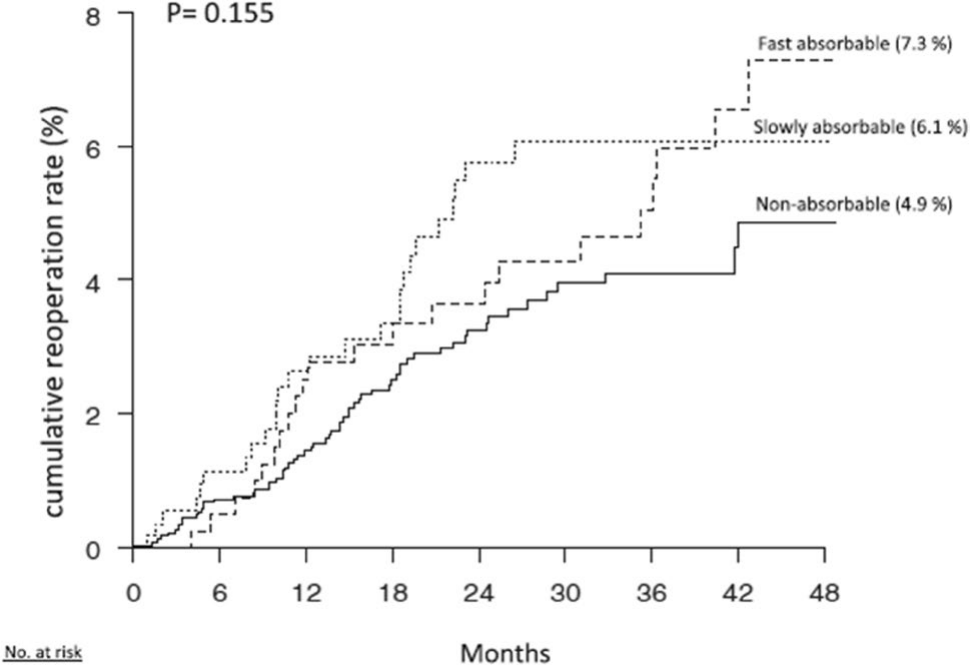
**B** Kaplan–Meier plot illustrating the cumulated risk of reoperation for recurrences for the three different suture types in the sutured repair group (log rank, *p* = 0.155)

The authors conclude that mesh reinforcement should be a routine in even small umbilical or epigastric hernias to lower the risk of reoperation for recurrence.

Important results were reported by Koehler et al. [10] in 2015. Patients with rectus diastasis suffered from a significantly increased rate of hernia recurrence (29/93 vs. 9/108; *p* = 0.001). The use of absorbable sutures also had a negative influence on the recurrence rate (26/90 vs. 12/111; *p* = 0.001). The authors strongly recommend preoperatively checking for rectus diastasis and using non-absorbable sutures as an alternative to mesh repair only when repairing small umbilical or epigastric hernias (< 2 cm) and there is no concomitant rectus diastasis. Patients with coexistent rectus diastasis definitely benefit from mesh-based repair of the midline to decrease the recurrence rate.

Ponten et al. [11] published 2015 retrospective comparison of suture and mesh repair in 235 epigastric hernia. Recurrence rate was 10.9% (*n* = 6) compared to 14.9% (*n* = 20) in the suture repair group. Recurrence occurred more often after sutured repair compared to mesh repair. No difference in chronic pain was seen between mesh and suture repaired patients.

The article of Ponten in 2014 [13] “A Collective Review on Mesh-Based Repair of Umbilical and Epigastric Hernias” summarizes the topic: 20 articles were selected according to the evidence. Primary outcome was the recurrence rate, while secondary outcomes were complications and postoperative pain. The pooled results of the studies are difficult to interpret: inhomogeneous data, different techniques, different mesh materials. One single study in the laparoscopic group reports 73.7% complication rate and distorts massively the overall results.

**Table:** 

Approach	Recurrence (%), CI (n/N)	Complications (%), CI (n/N)	Postoperative pain (%), CI (n/N)
Laparoscopic	1.0, 0.0–2.0 (3/312)	25.2, 18.1–32.3 (36/143)	0.0, 0.0–11.5 (0/26)
Open	2.3, 1.4–3.2 (25/1068)	10.2, 8.1–12.2 (85/835)	9.8, 7.0–12.6 (42/429)

N = total of which laparoscopic or open recurrence/complication/postoperative pain rate was noted

Another interesting aspect is the RCT of Armañanzas [17]—Prophylactic mesh vs suture in the closure of the umbilical trocar site after laparoscopic cholecystectomy in high-risk patients for incisional hernia. Their conclusion: Mesh closure of the umbilical trocar site after laparoscopic surgery could become the standard method for preventing trocar site incisional hernia in high-risk patients.

**Author’s comment:**

In terms of recurrence, the available evidence is sufficiently strong to recommend that all defects of the abdominal wall, whether inguinal, incisional, or umbilical hernias, and of whatever size should be repaired with the use of prosthetic mesh. However, mesh repair is associated with higher costs and probably with a higher rate of local complications and chronic pain. Cost/benefit analyses are urgently needed definitely to answer the key question.

Despite the existing evidence, suture repair is still very popular in the surgical community.

**References** (in parenthesis the level of evidence)

1. Mathes T, Walgenbach M, Siegel R (2016) Suture Versus Mesh Repair in Primary and Incisional Ventral Hernias: A Systematic Review and Meta-Analysis. World J Surg 40:826–835 10.1007/s00268-015-3311-2 (1A)
2. Holihan JL, Hannon C, Goodenough C, Flores-Gonzalez JR, Itani KM, Olavarria O, Mo J, Ko TC, Kao LS, Liang MK (2017) Ventral Hernia Repair: A Meta-Analysis of Randomized Controlled Trials. Surg Infect (Larchmt) 18(6):647–658. 10.1089/sur.2017.029. Epub 2017 May 30. (2A)
3. Nguyen MT, Berger RL, Hicks SC, Davila JA, Li LT, Kao LS, Liang MK (2014)Comparison of outcomes of synthetic mesh vs suture repair of elective primary ventral herniorrhaphy: A systematic review and meta-analysis. JAMA Surg 149:415–421. (2A)
4. Kaufmann R, Halm JA, Eker HH, Klitsie PJ, Nieuwenhuizen J, van Geldere D, Simons MP, van der Harst E, van ‘t Riet M, van der Holt B, Kleinrensink GJ, Jeekel J, Lange JF (2018) Mesh versus suture repair of umbilical hernia in adults: a randomised, double-blind, controlled, multicentre trial. Lancet 391(10123):860–869. (2A)
5. Helgstrand F, Rosenberg J, Kehlet H et al. (2012). Reoperation for recurrence vs. clinical recurrence rate after ventral hernia repair. Ann Surg 256:955–958 (2C)
6. Helgstrand F, Jørgensen LN, Rosenberg J et al. (2013) Nationwide prospective study on readmission after umbilical or epigastric hernia repair. Hernia. 10.1007/s10029-013-1120-9. (2B)
7. Christoffersen MW, Helgstrand F, Rosenberg J, Kehlet H, Bisgaard T (2013) Lower Reoperation Rate for Recurrence after Mesh versus Sutured Elective Repair in Small Umbilical and Epigastric Hernias. A Nationwide Register Study World J Surg 37:2548–2552 (2C)
8. Westen M, Christoffersen MW, Jorgensen LN, Stigaard T, Bisgaard T (2013) Chronic complaints after simple sutured repair for umbilical or epigastric hernias may be related to recurrence. Langenbecks Arch Surg 399:65–69. (3)
9. Christoffersen MW, Helgstrand F, Rosenberg J, Kehlet H, Strandfelt P, Bisgaard T (2015) Long-term recurrence and chronic pain after repair for small umbilical or epigastric hernias: a regional cohort study. Am J Surg 209:725–732. (3)
10. Koehler G, Luketina RR, Emmanuel K (2015) Sutured Repair of Primary Small Umbilical and Epigastric Hernias: Concomitant Rectus Diastasis Is a Significant Risk Factor for Recurrence. World J Surg 39:121–126 (3)
11. Ponten JE, Leenders BJ, Charbon JA, Nienhuijs SW (2015) A consecutive series of 235 epigastric hernias. Hernia 19(5):821–5 (3B)
12. Bensaadi H, Paolino L, Valenti A, Polliand C, Barrat C, Champault G (2014) Intraperitoneal tension-free repair of a small midline ventral abdominal wall hernia: randomized study with a mean follow-up of 3 years. Am Surg 80(1):57–65. (2B)
13. Ponten JE, Thomassen I, Nienhuijs SW (2014) A Collective Review on Mesh-Based Repair of Umbilical and Epigastric Hernias. Indian J Surg 76(5):371–7. 10.1007/s12262-013-0920-6. Epub 2013 Apr 28. (3A)
14. Earle DB, McLellan JA. Repair of umbilical and epigastric hernias (2013) Surg Clin North Am 93(5):1057–89. 10.1016/j.suc.2013.06.017. (2)
15. Stabilini C, Bracale U, Pignata G, Frascio M, Casaccia M, Pelosi P, Signori A, Testa T, Rosa GM, Morelli N, Fornaro R, Palombo D, Perotti S, Bruno MS, Imperatore M, Righetti C, Pezzato S, Lazzara F, Gianetta E (2013) Laparoscopic bridging vs. anatomic open reconstruction for midline abdominal hernia mesh repair [LABOR]: single-blinded, multicenter, randomized, controlled trial on long-term functional results. Trials 14:357. 10.1186/1745-6215-14-357. Ongoing trial
16. Ponten JE, Leenders BJ, Charbon JA, Lettinga-van de Poll T, Heemskerk J, Martijnse IS, Konsten JL, Nienhuijs SW (2014) Mesh Or Patch for Hernia on Epigastric and Umbilical Sites (MORPHEUS trial): study protocol for a multi-centre patient blinded randomized controlled trial. BMC Surg 14:33. 10.1186/1471-2482-14-33. Ongoing trial
17. Armañanzas L, Ruiz-Tovar J, Arroyo A, García-Peche P, Armañanzas E, Diez M, Galindo I, Calpena R (2014) Prophylactic mesh vs suture in the closure of the umbilical trocar site after laparoscopic cholecystectomy in high-risk patients for incisional hernia. A randomized clinical trial. J Am Coll Surg 218(5):960–8. 10.1016/j.jamcollsurg.2014.01.049. Epub 2014 Feb 18. (4)
18. Abo-Ryia MH, El-Khadrawy OH, Moussa GI, Saleh AM (2015) Prospective randomized evaluation of open preperitoneal versus preaponeurotic primary elective mesh repair for paraumbilical hernias. Surg Today 45(4):429–33. 10.1007/s00595-014-0907-3. Epub 2014 May 3. (1B)
19. Bessa SS, El-Gendi AM, Ghazal AH, Al-Fayoumi TA (2015) Comparison between the short-term results of onlay and sublay mesh placement in the management of uncomplicated para-umbilical hernia: a prospective randomized study. Hernia 19(1):141–6. 10.1007/s10029-013-1143-2. Epub 2013 Aug 10. (3)
20. Eriksen JR, Bisgaard T, Assaadzadeh S, Jorgensen LN, Rosenberg J (2013) Fibrin sealant for mesh fixation in laparoscopic umbilical hernia repair: 1-year results of a randomized controlled double-blinded study. Hernia 17(4):511–4. 10.1007/s10029-013-1101-z. Epub 2013 May 9. (1B)
21. Malik AM (2015) Laparoscopic versus open repair of para-umbilical hernia. Is it a good alternative? J Pak Med Assoc 65(8):865-8. (2C)
22. Cassie S, Okrainec A, Saleh F, Quereshy FS, Jackson TD (2014) Laparoscopic versus open elective repair of primary umbilical hernias: short-term outcomes from the American College of Surgeons National Surgery Quality Improvement Program. Surg Endosc 28: 741–746. (3)
23. Kulaçoglu H (2015) Current options in umbilical hernia repair in adult patients. Ulus Cerrahi Derg 31(3):157–61. 10.5152/ucd.2015.2955. eCollection 2015. (4)
24. Kulacoglu H, Yazicioglu D, Ozyaylali I (2012) Prosthetic repair of umbilical hernias in adults with local anesthesia in a day-case setting: a comprehensive report from a specialized hernia center. Hernia 16(2):163–170 (4)
25. Helgstrand F, Bisgaard T (2018) Time for use of mesh repair for all umbilical hernias? Lancet 391(10123): 821–822 (4)
26. A. Winsnes, Haapamäki MM, Gunnarsson U, Strigård K (2016) Surgical outcome of mesh and suture repair in primary umbilical hernia: postoperative complications and recurrence. Hernia 20:509–516 10.1007/s10029-016-1466-x (3B)
27. Porrero JL, Cano-Valderrama O, Marcos A, Bonachia O, Ramos B, Alcaide B, Villar S, Sánchez-Cabezudo C, Quirós E, Alonso MT, Castillo MJ (2015) Umbilical Hernia Repair: Analysis after 934 Procedures. Am Surg 81(9):899–903. (3B)

## Chapter 6. Obese patients and incisional hernia

### F. Köckerling, P. Chowbey, R. Khullar

**Question:**

Should obese patients be preferably operated on by laparoscopic technique?

**Search terms**

The following search terms were used:

“incisional hernia”; “incisional hernia and obesity”; “laparoscopic incisional hernia repair”; “laparoscopic ventral hernia repair”; “ventral hernia.”

**Search machines**

PubMed, Medline, and the Cochran Library as well as the reference lists of the included studies were searched for relevant studies. For the study of the original guidelines, read the publication in “Surg Endosc (2014) 28: page 15–16.”

**New publications**

A total of 10 new studies were identified since the publication of the original guidelines. Statements and recommendations were modified accordingly.

**New statements:**

**Table:** 

Level 1A	Laparoscopic ventral and incisional hernia repair is associated with fewer wound infections and wound complications (stronger evidence)
Level 2C	A BMI higher than 30 kg/m^2^ significantly increases the risk of recurrence (stronger evidence)
Level 4	Spinal anesthesia for laparoscopic ventral hernia repair in obese patients can be an alternative to general anesthesia (new statement)

**New recommendations**

**Table:** 

Grade A	For obese patients presenting with a ventral or incisional hernia, the laparoscopic approach is preferred because it reduces the wound infection and wound complication rates (stronger evidence)
Grade B	As the recurrence risk for obese patients is higher, there may be a need for additional technical steps (greater mesh fixation, more overlap, suture closure of the defect) when the laparoscopic approach is indicated (stronger evidence)

**Comments**

In two meta-analyses and systematic reviews of laparoscopic vs open incisional hernia repair, the wound infection and wound complication rates are significantly higher after open repairs [1–4]. The risk of infection for laparoscopic compared to open surgery was almost five times lower for laparoscopy (OR 0.22; 95% CI [0.11–0.44]) (1). Statistically significant reduction in wound complications was noted with laparoscopic surgery compared to the open repair based on six randomized controlled trials (RCTs) (OR 0.21; 95% CI [0.07–0.64]; *p* = 0.01) (4). A systematic review and meta-analysis of RCTs comparing laparoscopic with open surgery in a mixed surgical population found surgical site infection rate after laparoscopic surgery significantly lower (OR 0.33; 95% [0.26–0.42]; *p* = 0.00001). Laparoscopic surgery in obese patients reduces surgical site infection rate by 70–80% compared with open surgery (5). In a retrospective analysis of data from the database of the American College of Surgeons National Surgical Quality Improvement Program from 2005 to 2015, the cohort consisted of 102,191 patients with open ventral hernia repair, 58.5% of whom were obese. When stratified by body mass index class, higher classes were associated with all postoperative complications (*p* < 0.0001) with a steady increase in complication rates with increasing body mass index class (6). In a nationwide hospital survey comparing laparoscopic vs open ventral hernia repair in obese patients (*n* = 47,661), the laparoscopic repair was associated with a lower overall complication rate (6.3% vs 13.7%; *p* < 0.001), shorter median length of stay (3 vs 4 days, *p* < 0.001), and lower mean total hospital charges ($ 40.387 vs $ 48.513; *p* < 0.001) (7). In a registry-based multivariable analysis of 5214 patients with laparoscopic repair of incisional hernias, the recurrence rate in 1-year follow-up was significantly higher in obese patient in comparison to normal weight patients (OR 1.621; 95% CI [1.138–2.309]; *p* = 0.007) (8). In updated guidelines of a consensus development conference endorsed from the European Hernia Society and the European Association of Endoscopic Surgery on laparoscopic ventral and incisional hernia repair, the laparoscopic repair is strongly recommended in obese patients (9). In a case series of 23 obese patients with BMI > 30 kg/cm^2^, spinal anesthesia for laparoscopic ventral hernia repair proved an efficient and safe alternative to general anesthesia (10). No conversion were recorded from both the anesthetic and the surgical point of view (10). No major intra- and postoperative complications were reported.

**References** (in parentheses the level of evidence)

1. Al Chalabi H, Larkin J, Mehigan B, McCormick P (2015) A systematic review of laparoscopic versus open abdominal incisional hernia repair, with meta-analysis of randomized controlled trials
2. International Journal of Surgery 20:65–74. 10.1016/j.ijsu.2015.05.050. Epub 2015 Jun 12. Review (1A)
3. Awaiz A, Rahman F, Hossain MB, Yunus RM, Khan S, Memon B, Memon MA (2015) Meta-analysis and systematic review of laparoscopic versus open mesh repair for elective incisional hernia. Hernia 19:449–463. 10.1007/s10029-015-1351-z (1A)
4. Jensen KK, Jorgensen LN (2015) Comment to: Meta-analysis and systematic review of laparoscopic versus open mesh repair for elective incisional hernia. Awaiz A et al. Hernia 2015; 19:449–463. Hernia 19:1025–1026. 10.1007/s10029-015-1412-3 (5)
5. Awaiz A, Rahman F, Hossain MB, Yunus RM, Khan S, Memon B, Memon MA (2015) Reply to comment to Meta-analysis and systematic review of laparoscopic versus open mesh repair for elective incisional hernia. Jensen K, Jorgenson LN. Hernia 19:1027–1029. 10.1007/s10029-015-1432-z (1A)
6. Shabanzadeh DM, Sørensen LT (2012) Laparoscopic surgery compared with open surgery decreases surgical site infection in obese patients: a systematic review and meta-analysis. Ann Surg 256(6):934–45. 10.1097/sla.0b013e318269a46b (1A)
7. Owei L, Swendiman RA, Kelz RR, Dempsey DT, Dumon KR (2017) Impact of body mass index on open ventral hernia repair: A retrospective. Review. Surgery 162 (6): 1320–1329. DOI: 10.1016 (3)
8. Lee J, Mabardy A, Kermani R, Lopez M, Pecquex N, McClune A (2013) Laparoscopic vs open ventral hernia repair in the era of obesity. JAMA Surg 148(8):723–6. 10.1001/jamasurg.2013.1395 (2C)
9. Köckerling F, Simon T, Hukauf M, Hellinger A, Fortelny R, Reinpold W, Bittner R (2018) The Importance of Registries in the Postmarketing Surveillance of Surgical Meshes. Ann Surg 268(6):1097–1104. 10.1097/sla.0000000000002326. [Epub ahead of print]
10. Silecchia G, Campanile FC, Sanchez L, Ceccarelli G, Antinori A, Ansaloni L, Olmi S, Ferrari GC, Cuccurullo D, Baccari P, Agresta F, Vettoretto N, Piccoli M (2015) Laparoscopic ventral/incisional hernia repair: updated guidelines from the EAES and EHS endored Consensus Development Conference. Surg Endosc 29:2463–2484. 10.1007/s00464-015-4293-8 (1A)
11. Symeonidis D, Balyiannis I, Georgogpulou S, Koukoulis G, Athanasiou E, Tzovaras G (2013) Laparoscopic ventral hernia repair in obese patients under spinal Anesthesia. Int J Surg 11: 926–929. 10.1016/j-ijsu.2013.07.002 (4)

## Chapter 7. Recurrence after open surgery—re-do better laparoscopically?

### R Schrittwieser, F. Berrevoet

**Question:**

Is a reoperation after open surgery better done laparoscopically?

**Search terms:**

(open [All Fields] AND (“hernia, ventral [MeSH Terms]” OR (“hernia”[All Fields] AND “ventral”[All Fields]) OR “ventral hernia”[All Fields] OR (“ventral”[All Fields] AND “hernia”[All Fields]) AND (“recurrence”[MeSH Terms] OR “recurrence”[All Fields])

**Search machines:**

PubMed, Medline, and the Cochrane Library as well as the reference lists of the included studies were searched for relevant studies.

**New publications**

A total of 3 new studies were identified since the publication of the original guidelines. Statements and recommendations were modified accordingly. For the study of the original guidelines, read the publication in “Surg Endosc (2014) 28: page 16–17.”

**New statements**:

**Table:** 

Level 4	Complication rates after laparoscopic repair vary according to both hernia and patient characteristics and type of previous repairs
Level 4	There is no consensus in the literature on the complication rate according to previously used techniques

**New recommendation:**

**Table:** 

Grade C	It is recommended that each case of recurrent hernia should be evaluated separately to judge best treatment

**Comments:**

Ferrari et al. [1] reported a mean follow-up of 41 months and could not find any differences in the postoperative outcome between primary and recurrent ventral hernias after treatment of 69 patients. As their group of patients was treated over a 10-year period, the patients may be selected and rather small meshes were used in their bridging technique. Meyer et al. [2] published on 149 patients with 34 recurrent hernias. The recurrence rate for primary hernias was 3.9%, for incisional hernias 11%, and for recurrent hernias 26.5%. Picazo-Yeste et al. [3] reported on 124 patients, of which 96 had recurrent hernias, with rather small defects. After a mean follow-up of 30 months they observed 3 recurrences (2.6%). In an univariate analysis related to demographic, clinical, and perioperative variables, no significant relationship between the number of previous recurrences and operating time, conversion rate, hospital length of stay, overall morbidity, or recurrence was identified.

However, it should be emphasized that reinforcement of the complete scar is recommended for recurrent hernias, in both open and laparoscopic repair techniques.

**References** (in parentheses the level of evidence)

1. Ferrari G, Bertoglio C, Magistro C, Girardi V, Mazzola M, Di Lernia S, Pugliese R (2013) Laparoscopic repair for recurrent incisional hernias: a single institute experience of 10 years, Hernia 17(5):573–580 (4)
2. Meyer R, Häge A, Zimmermann M, Bruch HP, Keck T, Hoffmann M, Schlöricke E (2015) Is laparoscopic treatment of incisional and recurrent hernias associated with an increased risk for complications? Int J Surg 19:121–127 (4)
3. Picazo-Yeste J, Moreno-Sanz C, Sedano-Vizcaino C, Morandeira-Rivas A, Sanchez-De Pedro F (2017) Outcomes after laparoscopic ventral hernia repair: does the number of previous recurrences matter? A prospective study, Surg Endosc 31(11):4514–4521 (4)

## Chapter 8. Evidence for antibiotic and thromboembolic prophylaxis in laparoscopic ventral hernia surgery

### Rudolf Schrittwieser, B. Stechemesser

**Key questions:**

1. Is there evidence for antibiotic prophylaxis in laparoscopic ventral hernia repair?
2. Is there evidence for thromboembolic prophylaxis in laparoscopic ventral hernia repair?

**Search terms**

The following search terms were used: “ventral hernia” AND “antibiotic prophylaxis”; “ventral hernia” AND “antibiotic prophylaxis” AND “laparoscopy”; “ventral hernia” AND “antibiotic prophylaxis” AND “randomized studies”; “abdominal wall hernia” AND “antibiotic prophylaxis”; “ventral hernia” AND “thromboembolic prophylaxis”; “hernia” AND “thromboembolic prophylaxis” AND “laparoscopy”; “ventral hernia” AND “thromboembolic prophylaxis” AND “randomized studies”; “abdominal wall hernia” AND “thromboembolic prophylaxis.”

**Search machines**

PubMed, Medline, and the Cochrane Library as well as the reference lists of the included studies were searched for relevant studies.

**New publications**

One new study was identified since the publication of the original guidelines. Statements and recommendations have not changed since the original guidelines. For the study of the original guidelines, read the publication in “Surg Endosc (2014) 28: page 17–18.”

**Table:** 

Unchanged Statements and recommendations	

**Comments**

In 2015, Bahar et al. published a study in Asian journal of surgery (1) where they investigated the role of prophylactic cephalosporin in the prevention of infection after various types of hernia repairs with mesh. In their conclusion they stated that it did not significantly decrease the risk of wound infection. But the study dealt only with open hernia repairs and in the incisional hernia group there were only 7 patients without antibiotic prophylaxis and 94 with prophylaxis. So these results are not relevant for the recommendations.

**References** (in parenthesis the level of evidence)

1. Mehrabi Bahar M, Jabbari Nooghabi A, Jabbari Nooghabi M, Jangjoo A (2015) The role of prophylactic cefazolin in the prevention of infection after various types of abdominal wall hernia repair with mesh. Asian J Surg 38(3):139–144. (2B)

## Chapter 9 Positioning of the trocars and creating the capno pneumoperitoneum

### Jie Chen, VK Bansal

**Key questions:**

1. Should an open access always be performed?
2. In which cases could the Veress needle be preferred for establishment pneumoperitoneum?
3. Where is the best place for the first access?

The following search terms were used: “laparoscopic” AND “ventral” AND “incisional” AND “abdominal wall” AND “hernia” AND “technique.” An updated systematic search of the literature was performed in September 2017 of Medline, PubMed, Cochrane Library, and relevant journals and reference lists using the above-listed search terms to include the period from January 2012 to September 2017. 53 articles were found and analyzed, and 3 articles were suitable to update this review. For the study of the original guidelines, read the publication in “Surg Endosc (2014) 28: page 18.”

**New statements**

**Table:** 

Level 2	There is no difference in major complication rates with direct trocar insertion without pneumoperitoneum compared with pneumoperitoneum with a Veress needle prior to initial trocar insertion
Level 2	Pneumoperitoneum creation with a Veress needle followed by entrance into the abdomen with an optical trocar is the method most frequently used
Level 4	The most safe place for insertion of the first trocar seems to be in the left (Palmer’s point) (or right) upper quadrant subcostally in the midclavicular line for midabdominal and lower abdominal hernias

**New recommendation**

**Table:** 

Grade B	It is recommended that the Veress needle and the first trocar should be inserted at Palmer’s point and as far as possible from the site of expected adhesions
Grade B	It is recommended that the surgeons should use the access technique that they are most skilled with
Grade C	It is recommended that secondary port placement should be performed under vision and placed as far as possible from the hernia defect and expected
	Adhesions as well to allow the surgeon to work in a favorable position for release of adhesions and placement/fixation of mesh

The method of creation of pneumoperitoneum for LVHR has not been reported in many published literature. Capno pneumoperitoneum can be obtained using an open or closed technique [1] including the use of the Veress needle, direct trocar insertion with an optical trocar, and an open Hasson’s technique. The method used for capno pneumoperitoneum creation should be chosen according to suspected presence of adhesions, the size, site, and number of hernial defects and surgeon’s experience [2].

Although there are no studies available in the literature comparing the different methods of pneumoperitoneum creation in patients undergoing LIVHR, but in multiple meta-analyses and randomized controlled trials in patients undergoing general surgical and gynecological laparoscopic procedures, there was no difference in major complication rates with direct trocar insertion without pneumoperitoneum compared with the establishment of pneumoperitoneum with Veress needle prior to initial trocar insertion [2].

Regardless of the technique used, great care must be exercised to minimize the risk of complications such as vascular or visceral injuries. Placement of a Veress needle in a reoperative abdomen may be difficult and requires familiarity with this technique. In general, the surgeon should use the access technique that they are most skilled with [1].

Most surgeons prefer the creation of a pneumoperitoneum with a Veress needle followed by entrance into the abdomen with an optical trocar [2].The first trocar is optimally placed in the left (Palmer’s point) (or right) upper quadrant subcostally in the midclavicular line for midabdominal and lower abdominal hernias [3].

To facilitate instrument manipulation along with adequate visualization during laparoscopy, trocars usually are placed in triangular fashion termed triangulation. It is recommended that the trocar entry points should be as far as possible from the site of expected adhesions and the size, site, and number of wall defects, and that they should be placed to achieve triangulation of the hernia site [2].

**References** (in parenthesis the level of evidence)

1. Hope WW, Hooks WB 3rd. (2013) Atypical hernias: suprapubic, subxiphoid, and flank. Surg Clin N Am 93(5):1135–62. (2A)
2. Earle D, Roth S, Saber A, et al. (2016) SAGES guidelines for laparoscopic ventral hernia repair. Surg Endosc 30(8):3–3183. (1A)
3. Alexander AM, Scott DJ (2013) Laparoscopic ventral hernia repair. Surg Clin North Am 93(5): 1091–110. (2A)

## Chapter 10 Port type, positions, and number in laparoscopic ventral hernia repair

### David Radvinsky, Mazen Iskandar, George Ferzli

**Key questions:**

1. Should radially expanding trocars or cutting trocars be used?
2. What is the optimal size of the trocars?
3. What is better—0°, 30°, or 45° optic?
4. How many trocars should be used?
5. Should additional contralateral trocars routinely be used?

The following search terms were again used: “laparoscopic” AND “ventral” AND “incisional” AND “abdominal wall” AND “hernia” AND “technique.” A systematic search of the literature was performed in May 2017 using PubMed, and the Cochrane Library, as well a search of reference lists for the time period of January 2012 to May 2017 to cover the update period. Additional 51 articles were found and analyzed. Seven additional articles were used to bolster this review.

After careful analysis of all articles, including those used in the original guidelines and since 2012, the statements and recommendations made from the original guidelines remain valid. The additional literature review supports and improves on the level of evidence and grade of recommendation for placement of trocars for laparoscopic ventral hernia. Additional recommendations based on new literature since the previous guidelines have been added on the use of radially expanding blunt-tipped trocars. For the study of the original guidelines, read the publication in “Surg Endosc (2014) 28: page 18–19.”

**New statement**

**Table:** 

Level 2	The use of radially expanding blunt-tipped trocars are associated with lower risk of trocar site bleeding, but data are lacking for major trocar-related complications between trocar types

**New recommendation**

**Table:** 

Grade C	It is recommended to use radially expanding blunt tip trocars where possible to reduce port-site bleeding

Port positions and number

The fundamental principles of laparoscopic and robotic-assisted surgery, namely, triangulation around the operative field and optimal distance (16–18 cm) from the target, apply to laparoscopic and robotic ventral hernia surgery [1]. Since the original publication, there has been an increase in literature on robotic-assisted ventral hernia repair. The principles of port positioning, type, and number have not appeared to vary based on operative technique or robotic platform [2].

Entry into the abdomen is performed per the surgeon’s preference and skill. The first trocar should always be placed as far as possible laterally from the defect to provide clear visualization of the defect margin and mesh overlap. The majority of surgeons prefer the left hypochondrium at Palmer’s point. This trocar is typically 8–12 mm to accommodate a 10-mm camera and mesh insertion. In dealing with midline and right-sided abdominal wall defects, three inline trocars in the left abdomen are ideal. Left-sided abdominal defects are approached via three trocars on the right. For abdominal wall defects > 10 cm, one or two trocars can be inserted on the opposite side of the abdomen to perform safe mesh fixation [3, 4].

Small subxiphoid defects can be managed with the patient in a modified lithotomic position and with the surgeon between the patient’s legs. The camera port is placed at the umbilicus, and a 5-mm trocar on each side provides excellent triangulation around the hernia. For larger subxiphoid defects, ports are arranged as lateral as possible along the flanks, to facilitate range of motion of instruments and mesh overlap, typically in a semicircular configuration with the lowermost port closest to midline [3–5].

Suprapubic defects can be managed in a similar fashion. For smaller defects, the umbilicus can be used as the camera port with two small working ports on either side of the abdomen. Larger suprapubic hernias also can be repaired using three left-flank trocars in a semicircular fashion, with the uppermost port closer to the midline [3–6]. Additional ports can be placed in the right flank. This certainly will be of benefit for difficult cases in which extensive adhesiolysis is required or a large hernia sac is encountered.

Port type

Visually guided insertion of trocars does not decrease the incidence of visceral or vascular injury but does decrease the size of the port-site wounds [7]. Additionally, using radially expanding blunt-tipped trocars has been associated with a lower risk of trocar site bleeding compared to bladed trocars. Data are lacking on the incidence of major trocar-related complications comparing radially expanding vs conical blunt-tipped and cutting trocars [8, 9].

**References** (in parenthesis the level of evidence)

1. Ferzli GS, Fingerhut A (2004) Trocar placement for laparoscopic abdominal procedures: a simple standardized method. J Am Coll Surg 198(1):163–73. (5)
2. Gonzalez A, Escobar E, Romero R, Walker G, Mejias J, Gallas M, Dickens E, Johnson CJ, Rabaza J, Kudsi OY (2017)Robotic-assisted ventral hernia repair: a multicenter evaluation of clinical outcomes. Surg Endosc 31(3):1342–1349. (3)
3. Alexander AM, Scott DJ (2013) Laparoscopic ventral hernia repair. Surg Clin North Am 93(5):1091–110. (2)
4. Nardi M Jr, Millo P, Brachet Contul R, Lorusso R, Usai A, Grivon M, Persico F, Ponte E, Bocchia P, Razzi S (2017) Laparoscopic ventral hernia repair with composite mesh: Analysis of risk factors for recurrence in 185 patients with 5 years follow-up. Int J Surg. 40:38–44. (3)
5. Hope WW, Hooks WB 3^rd^ (2013) Atypical hernias: suprapubic, subxiphoid, and flank. Surg Clin North Am 93(5):1135–62. (2A)
6. Sharma A, Dey A, Khullar R, Soni V, Baijal M, Chowbey PK (2011) Laparoscopic repair of suprapubic hernias: transabdominal partial extraperitoneal (TAPE) technique. Surg Endosc 25(7):2147–52. (3)
7. Vilos GA, Ternamian A, Dempster J, Laberge PY (2007) Society of Obstetricians and Gynaecologists of Canada. Laparoscopic entry: a review of techniques, technologies, and complications. J Obstet Gynaecol Can 29(5):433–65. (2)
8. La Chapelle CF, Swank HA, Wessels ME, Mol BW, Rubinstein SM, Jansen FW (2015) Trocar types in laparoscopy. Cochrane Database Syst Rev Dec 16;(12). (1A)
9. Antoniou SA, Antoniou GA, Koch OO, Pointner R, Granderath FA (2013) Blunt versus bladed trocars in laparoscopic surgery: a systematic review and meta-analysis of randomized trials. Surg Endosc 27(7):2312–20 (1A)

## Chapter 11. Principles of adhesiolysis

### H. Hoffmann, J. Chen, K. LeBlanc

**Key question:**

What is the best dissection technique and what instruments should be used to avoid bowel lesions?

**Search items**

The following search terms were used: “hernia” AND “adhesiolysis,” “abdominal” AND “adhesiolysis,” and “abdominal” AND “adhesiolysis” AND “treatment.”

**Search machines**

Medline, PubMed, the Cochrane Library, as well as the reference lists of the included studies were searched for relevant studies.

**New publications**

A total of eight new studies were identified since the publication of the original guidelines. Statements and recommendations were modified accordingly. For the study of the original guidelines, read the publication in “Surg Endosc (2014) 28: pages 19–20.”

**New statements**

**Table:** 

Level 2c	Enterotomy due to adhesiolysis is the most common intraoperative complication in VHR of which half occur during adhesiolysis
	Extensive adhesiolysis predicts increased rates for morbidity, enterotomy, surgical site infection and length of hospital stay
	Compared to primary ventral hernia repair, incisional ventral hernia repair requires more adhesiolysis
Level 3	Adhesiolysis is necessary in majority of patients undergoing VHR
	Prolonged adhesiolysis time and preexisting intra-abdominal meshes are independent risk factors for enterotomy

**New recommendations**

**Table:** 

Grade B	Adhesiolysis should be limited to reduce the risk of inadvertent enterotomy
Grade D	Before completion of surgery, the bowel should carefully be inspected to identify any unrecognized enterotomies or thermal injury

**Comments**

Adhesiolysis is an important part of the laparoscopic ventral hernia repair, as nearly all incisional hernias exhibit adhesions to the abdominal wall, with relatively fewer adhesions associated with primary hernias (1). Inadvertent enterotomy is the most common intraoperative complication during adhesiolysis in abdominal procedures with an incidence up to 11% (2–4), this untoward event is known to lead to sepsis, intra-abdominal complications, surgical site infections, prolonged length of hospital stay, and a mortality rate up to 8% (4). Additionally, the laparoscopic approach itself may have a higher rate of intestinal injuries compared to the open approach, particularly in small bowel obstruction (5) and ventral hernia repair (6). Extended adhesiolysis during laparoscopic IPOM repair has also been shown to increase the risk for seroma formation (7). Also, adhesiolysis itself does not offer additional benefit such as less chronic abdominal pain (8). Therewith, adhesiolysis during laparoscopic ventral hernia repair should be limited to the expected landing zone of the mesh on the peritoneal surface of the abdominal wall to allow optimal mesh ingrowth and adequate mesh overlap.

**References (in parentheses the level of evidence)**

1. Stirler VMA, Schoenmaeckers EJP, De Haas RJ, Raymakers JTFJ, Rakic S (2014) Laparoscopic repair of primary and incisional ventral hernias: The differences must be acknowledged—A prospective cohort analysis of 1088 consecutive patients. Surg Endosc 28(3):891–895. (2b)
2. Mavros MN, Velmahos GC, Larentzakis A, et al. (2014) Opening Pandora’s box: understanding the nature, patterns, and 30-day outcomes of intraoperative adverse events. Am J Surg 208(4):626–631. (2c)
3. Mavros MN, Velmahos GC, Lee J, Larentzakis A, Kaafarani HMA (2014) Morbidity related to concomitant adhesions in abdominal surgery. J Surg Res. 192(2):286–292. (2c)
4. ten Broek RPG, Strik C, Issa Y, Bleichrodt RP, van Goor H (2013) Adhesiolysis-related morbidity in abdominal surgery. Ann Surg 258(1):98–106. (2b)
5. Behman R, Nathens AB, Byrne JP, Mason S, Look Hong N, Karanicolas PJ (2017) Laparoscopic Surgery for Adhesive Small Bowel Obstruction Is Associated With a Higher Risk of Bowel Injury: A Population-based Analysis of 8584 Patients. Ann Surg 266(3):489–498. (2c)
6. Ahonen-Siirtola M, Rautio T, Ward J, Kössi J, Ohtonen P, Mäkelä J (2015) Complications in Laparoscopic Versus Open Incisional Ventral Hernia Repair. A Retrospective Comparative Study. World J Surg 39(12):2872–2877. (3b)
7. Huang C-C, Lien H-H, Huang C-S (2013) Long-Term Follow-Up of Laparoscopic Incisional and Ventral Hernia Repairs. J Laparoendosc Adv Surg Techn 23(3):199–203. (3c)
8. Strik C, Stommel MWJ, Hol JC, van Goor H, ten Broek RPG (2017) Quality of life, functional status and adhesiolysis during elective abdominal surgery. Am J Surg 215(1):104–112. (2b)

## Chapter 12. Laparoscopic ventral or incisional hernia repair—importance of defining hernial defect margins and gaging the size of the hernia preoperatively and intraoperatively

### P. Chowbey, A. Sharma

**Key questions:**

1. How to measure the size of the hernia defect intraoperatively?
2. Which method is more reliable, the pre- or the intraoperative measurement?

**Search terms:**

A systematic search and review of the literature was performed in Pubmed, Medline, the Cochrane Library, EMBASE, the British Journal of Surgery database, UK Pubmed Central, Google, Google scholar, Scirus, Ovid, and the Directory of Open Journal Access (DOAJ). The following search terms were used: “hernial defect size,” “hernial defect margins,” “hernial defect diameter,” “hernial defect area,” “laparoscopic contraindications,” “mesh size,” “measuring hernial defect size,” “incisional hernia,” and “ventral hernia.”

**Search machines:**

PubMed, Medline, and the Cochrane Library as well as the reference lists of the included studies were searched for relevant studies.

**New Publications:** A total of 5 new studies were identified since the publication of original guidelines. For original guidelines, read the publication in “Surg Endosc (2014) 28: page 20–21.” The previous statements and recommendations are still valid.

**New statements**

**Table:** 

Level 2b	Among common methods of measuring abdominal wall hernia defect, sizes are only weakly to moderately correlated
Level 2a	Large defect widths and total area have a greater chance of pain and activity limitation at 1-month follow-up, but not long term
Level 3	Dynamic rather than static measurements of ventral hernia area during laparoscopy provide a simple way of in vivo objective measurement that helps the surgeon choose the appropriate size of mesh
Level 1	Despite the ability to characterize ventral hernia morphology and recurrence with precision, most indexed studies do not employ imaging

**New recommendation**

**Table:** 

Grade B	Dynamic rather than static measurements of ventral hernia area during laparoscopy are recommended

**Discussion:**

A prospective randomized trial involving 50 patients and 5 different modalities of measuring hernia defect showed weak correlation for length, moderate correlation for width, and moderate correlation for area. Different types of measurements affected mesh selection in up to 56% of cases (1).

In a retrospective study of 865 patients in International Hernia mesh registry, patients were classified on the basis of hernia defect. They were classified into large, ≥ 10 cm and small, < 10 cm, along with area as large, ≥ 100 cm and small, < 100 cm. Larger defects were associated with greater chance of pain and activity limitation at 1 month (2).

In a prospective study of lap ventral hernia repair, defect was measured using a sterile paper ruler at IAP-8 mmHg and IAP-15 mmHg. Changing the IAP significantly changed the values of horizontal and vertical measurements, and consequently the area of hernia defect. The mesh required to cover the defect increased by a median of 5% (3)

In a systematic review done by Parker et al., out of 31 RCTs only 14 (45%), 11 (35%), and 6 (20%) trials reported CT measurements of hernia defect area, width, and loss of domain, respectively [4–5].

**References** (in parenthesis the level of evidence)

1. Cherla, D.V., Lew, D.F., Escamilla, R.J. et al. (2018) Differences of alternative methods of measuring abdominal wall hernia defect size: a prospective observational study. Surg Endosc 32: 1228. [2B]
2. Wormer BA, Walters AL, Bradley JF 3rd et al. (2013) Does ventral hernia defect length, width, or area predict postoperative quality of life? Answers from a prospective, international study. J Surg Res 184(1):169–177 [2A]
3. Qandeel H, O’Dwyer PJ (2016) Relationship between ventral hernia defect area and intra-abdominal pressure: dynamic in vivo measurement. Surg Endosc 30:1480–1484 [3]
4. Halligan S, Parker SG, Plumb AAO, Wood CP, Bolton RW, Mallett S, Windsor AC (2018) Use of imaging for pre- and post-operative characterisation of ventral hernia: systematic review. The British Journal of Radiology 91(1089):20170954 [1]
5. Parker SG, Wood CPJ, Butterworth JW, Boulton RW, Plumb AAO, Mallett S, Halligan S, Windsor ACJ (2018) A systematic methodological review of reported perioperative variables, postoperative outcomes and hernia recurrence from randomised controlled trials of elective ventral hernia repair: clear definitions and standardised datasets are needed. Hernia 22:215–26. [1]

## Chapter 13. Bridging–augmentation–reconstruction of the linea alba—closure of the defect before IPOM

### J Kukleta, D. Chen, P. Chowbey, A. Sharma

**Key questions:**

1. Is closure of the hernia defect superior to non-closure regarding postoperative complications?
2. Is closure of the hernia defect superior to non-closure regarding recurrence rate?
3. In large hernia repairs, are there additional measures available in order to facilitate the defect closure/reconstruction of linea alba?

**Search terms:**

“augmentation repair” AND “incisional hernia” AND “bridging repair” AND “defect closure”; “hybrid repair” AND “linea alba reconstruction” AND “incisional hernia”; “augmentation repair” AND “incisional hernia” AND “bridging repair” AND “defect closure”; “hybrid repair” AND “linea alba reconstruction” AND “incisional hernia”

A systematic search of the available literature using Pubmed, Medline, and Cochrane Library, as well as search of other relevant journals and reference lists was performed in September 2017. Applying the filter of last 5 years, there were 1242 articles for “ventral hernia repair,” 755 for “incisional hernia repair,” and 1720 for “abdominal hernia repair” to start with. Additional filters like meta-analysis, RCT, systematic review, and review reduced the number to 26, 58, 103, and 214, respectively. Respecting the topic of this chapter, 23 articles were included to this review. 4 Meta-analyses, 3 newer RCTs, and 16 comparative studies. For the study of the original guidelines, read the publication in “Surg Endosc (2014) 28: page 21–22.”

**New statements**

**Table:** 

Level 2A	The primary goal of the reconstruction of linea alba is the restitution of functionality of the abdominal wall. The improved cosmesis is a positive side effect
Level 2C	Closure of the defect prior to intraperitoneal onlay mesh (IPOM-Plus) results in less recurrence, seroma formation, and bulging in some studies.
	There are significantly fewer adverse events noted following the closure of fascial defect when compared to non-closure repair
	Closure of the fascial defect during laparoscopic ventral/incisional hernia repair reduces significantly seroma rate in the most studies
Level 3	The bridged repair (cIPOM, c = classic) is associated with a significantly higher risk of hernia recurrence and a higher overall complication rate
	IPOM-Plus repair patients show better satisfaction with the result in some studies and have better functional status
	Concomitant correction of diastasis recti in middle and lower abdomen defeats the muscular dysbalance of the trunk and its consequences
	Some studies did not demonstrate the advantage of the defect closure over the non-closure repair
	Some studies have reported similar postoperative outcomes in hernia defect closure and non-closure groups

**New recommendations 2017**

**Table:** 

Grade B	The restoration of normal anatomy (reconstruction of linea alba) during the laparoscopic abdominal wall repair should be attempted
Grade C	In case of too high tension while reconstructing linea alba, additional component separation techniques may be necessary
Grade D	In large hernia repairs, additional measures (temporary chemical components relaxation with Botulinum toxin A, preoperative progressive pneumoperitoneum, or intramuscular expanders) must be considered in order to facilitate the defect closure/reconstruction of linea alba

**Nguyen et al. [1]** performed a systematic review of 11 studies, including case series and retrospective reviews. Recurrence rate ranged from 0% to 7.7%, and seroma rates were 0% to 11.4%. Three of the retrospective reviews included compared laparoscopic hernia repairs with and without primary defect closure. Clapp et al. published the only risk-adjusted study and followed 72 cases for an average of 24 mo. Hernia recurrence was 16.7% in the group without primary defect closure, whereas no recurrences were seen in the group with primary defect closure. Bulging in this study was decreased from 69.4% in the non-closure group to 8.3% in the closure group. Three comparative studies examined the difference between closure and non-closure of the fascial defect in laparoscopic ventral incisional hernia repairs.

The recurrence rate ranged from 4.8 to 16.7% in the non-closure group and from 0 to 5.7% in the closure group. Seroma formation rate ranged from 4.3 to 27.8% in the non-closure group and from 5.6 to 11.4% in the closure group.

Closure of the central defect during LVHR resulted in less recurrence, bulging, and seroma than non-closure. Patients with closure were more satisfied with the results and had better functional status. The quality of the data was poor, however.

Primary fascial closure techniques: The techniques for closure varied. Overall, two studies used intracorporeal closure only, seven studies utilized an extracorporeal technique, and two studies had a mixed technique.

**Clapp et al. [2]** reported a retrospective study that examined the outcomes of defect closure. It was the only study that was risk-adjusted with minimum follow-up criteria. The median follow-up period was 24 months (7–34 months). In the non-closure group (*n* = 36), the recurrence rate was 16.7%. The closure group (*n* = 36) experienced no recurrences. Bulging rate was 69.4% in the non-closure group and 8.3% in the closure group. SSI rate was 13.9% in the non-closure group and 8.3% in the closure group. Seroma formation rate was 27.8% in the non-closure group and 5.6% in the closure group.

**Zeichen et al. [3]** performed a retrospective study that compared non-closure (*n* = 93) to closure of the primary fascia (*n* = 35). The mean follow-up period was 26 months (1–108 months). The study used both percutaneous (*n* = 18) and intracorporeal (*n* = 17) closure of the defect. The non-closure group had 15.1% recurrence rate. Two cases of recurrence appeared in the percutaneous closure group.

**Banerjee et al. [4]** published in 2012 a retrospective study. The recurrence rate was 4.8% in the non-closure group and 3.0% in the closure group.

**Results:** One hundred ninety-three consecutive patients underwent LVHR for incisional (*n* = 136), umbilical (*n* = 44), epigastric (*n* = 9), and parastomal (*n* = 4) hernia. Hernia recurrence was documented in eight patients (4.1%). The mean follow-up period was 10.5 months (range 1–36 months). Incisional hernias accounted for all eight recurrences. The rate of recurrence in closure group was 3% (2/67 in comparison with 4.8% (6/126) associated with mesh alone. The rate of recurrence in the recurrent hernia group, treated with mesh only, was 10.5% (4/38) compared with 4.8% (1/21) in the closure group.

Conclusions: Primary laparoscopic repair along with mesh placement for the management of ventral hernia was found to be effective in selected cases as evidenced by the low rate of recurrence when compared with conventional laparoscopic repair with mesh alone.

**Tandon et al. [5]** published in 2016 an important meta-analysis.

The primary outcome of interest was adverse events (recurrence, pseudorecurrence, mesh eventration, tissue eventration, or clinical eventration/bulging). Secondary outcomes were seroma, postoperative pain, mean hospital stay, mean duration of operation, and surgical techniques employed.

Results: A total of 16 studies were identified involving 3638 patients, 2963 in the CFD group, and 675 in the non-closure of facial defect group. Significantly fewer adverse events were noted following CFD than non-closure [4.9% (79 of 1613) versus 22.3% (114 of 511)], with a combined risk ratio (RR) of 0.25 (95% CI 0.18–0.33; *p* < 0.001). CFD resulted in a significantly lower rate of seroma [2.5% (39 of 1546) vs. 12.2% (47 of 385)], with a combined RR of 0.37 (0.23–0.57; *p* < 0.001), and shorter duration of hospital stay. No significant difference was noted in postoperative pain.

**Fig. 3:**
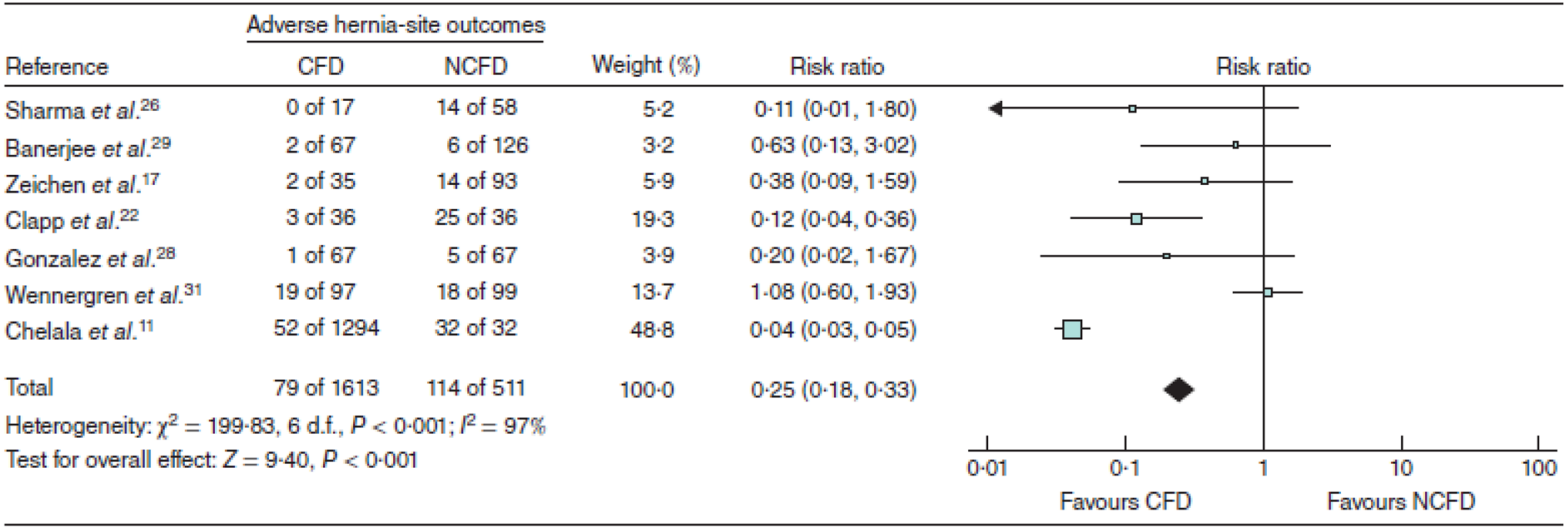
Forest comparing adverse hernia-site outcomes after laparoscopic incisional and ventral hernia repair with (CFD) and without (NCFD) closure of the fascial defect. A Mantel–Haenszel fixed-effect model was used for meta-analysis. Risk ratios are shown with 95% confidence intervals. From Tandon A et al. Br J Surg. 2016 Nov; 103(12):1598–1607

**Fig. 4:**
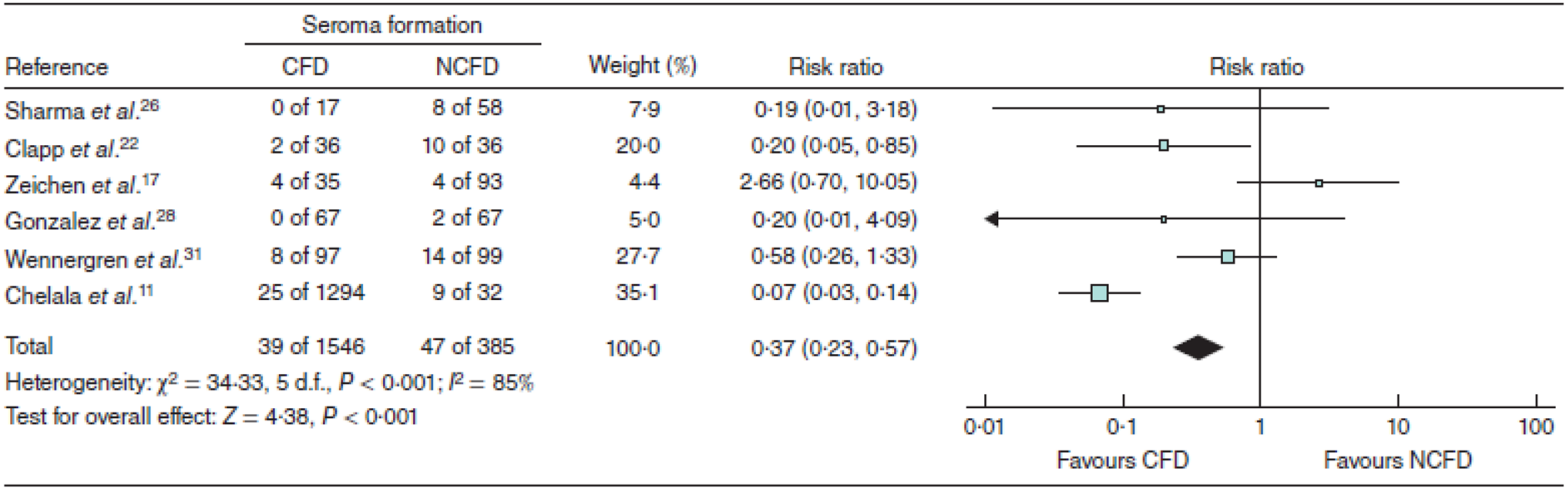
Forest plot comparing the formation after laparoscopic incisional and ventral hernia repair with (CFD) and without (NCFD) closure of the fascial defect. A Mantel–Haenszel fixed-effect model was used for meta-analysis. Risk ratios are shown with 95% confidence intervals. From Tandon A et al. Br J Surg. 2016 Nov; 103(12):1598–1607

Conclusion: Closure of fascial defect (CFD) during LIVHR reduces the rate of seroma formation and adverse hernia-site events (Figs. 3, 4).

**Table 1** Patient characteristics, study type, and quality scoring. From Tandon A et al. Br J Surg. 2016 Nov;103(12):1598–1607.
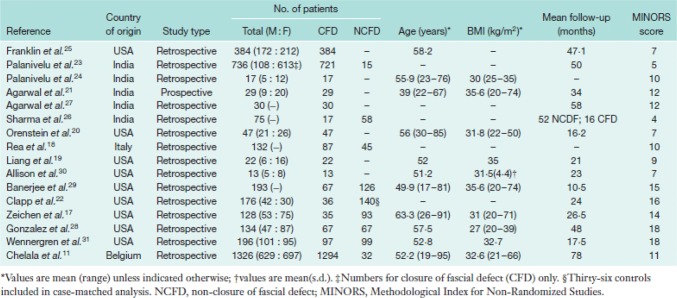


**Table 2** Hernia sizes and clinical outcomes. From Tandon A et al. Br J Surg. 2016 Nov;103(12):1598–1607.
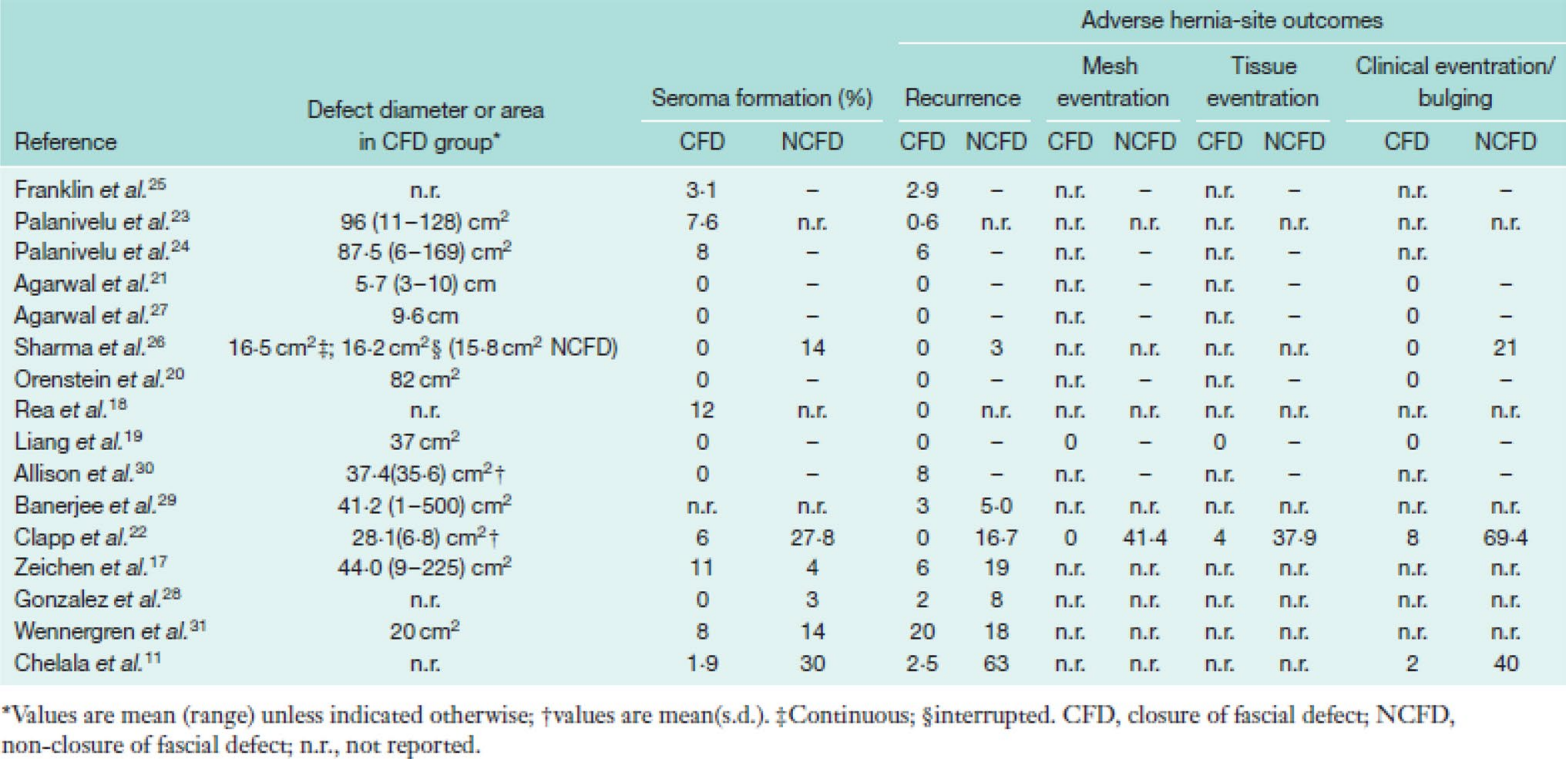


Seroma formation was reported in 15 studies with rates ranging from 0 to 12% (Table 2, Fig. 4). Six studies had a comparator non-fascial closure group. There were significantly higher rates of seroma formation after non-fascial closure [2.5% (39 of 1546) versus 12.2% (47 of 385)], with a combined RR of 0.37 (95% CI 0.23–0.57; *p* < 0.001).

Only non-randomized studies could be included. Their inclusion in meta-analysis remains controversial, but they are increasingly being included. Although RCTs are designed to minimize the risk of bias, their selection criteria are sometimes restrictive, which may not be the case with observational studies, which are often more representative of routine clinical practice.

In a meta-analysis of **Holihan et al.** [6] published in 2016, bridged repair was associated with more SSOs than was PFC (primary fascial closure), and PFC should be used whenever feasible. This study is included here for its clear statement about the superiority of augmentation repair compared to bridging repair. Endoscopic and perforator-sparing CS (components separation) were associated with the fewest complications; however, these conclusions are limited by heterogeneity between studies and poor methodological quality. These results should be used to guide future trials, which should compare the risks and benefits of each CS method to determine in which setting each technique will give the best results.

In a study by **Rea R et al.** [7], eighty-seven (43 over 65) patients were subjected to ventral hernia repair by laparoscopic approach with the primary closure of the hernial defect with external access. At ultrasound guidance in 15 days, 10 patients had moderate fluid collections (0.3 × 1.5 cm to 0.7 x4 cm). The primary closure of the hernia defect allows better to reinforce the wall, to reduce the dead space, and the possibility of formation of seromas. Its transparietal realization appears simple and safe, inexpensive of time.

**Booth et al.** [8] reported a retrospective review of prospectively collected data from consecutive patients with 1 year or more of follow-up, who underwent midline AWR between 2000 and 2011 at a single center. 222 patients (195 mesh-reinforced and 27 bridged repairs) with a mean follow-up of 31.1 ± 14.2 months were studied. The bridged repairs were associated with a significantly higher risk of hernia recurrence (56% vs 8%; hazard ratio [HR] 9.5; *p* < 0.001) and a higher overall complication rate [74% vs 32%; odds ratio (OR) 3.9; *p* < 0.001]. The interval to recurrence was more than 9 times shorter in the bridged group (HR 9.5; *p* < 0.001). Multivariate Cox proportional hazard regression analysis identified bridged repair and defect width > 15 cm to be independent predictors of hernia recurrence (HR 7.3; *p* < 0.001 and HR 2.5; *p* = 0.028, respectively).Conclusions: Mesh-reinforced AWRs with primary fascial coaptation resulted in fewer hernia recurrences and fewer overall complications than bridged repairs. Surgeons should make every effort to achieve primary fascial coaptation to reduce complications.

Review of the literature on IPOM-Plus in the PubMed database was published by **Suwa et al.** [9] in 2015. They identified 16 reports in which the recurrence rate, incidence of seroma formation, and incidence of mesh bulging were 0-7.7, 0-11.4, and 0%, respectively. Several comparison studies between IPOM and IPOM-Plus seem to suggest that IPOM-Plus is associated with more favorable surgical outcomes; however, larger-scale studies are essential. Primary outcome was hernia recurrence. Secondary outcomes were surgical site infection, seroma formation, bulging, and patient-centered items (satisfaction, chronic pain, functional status). Eleven studies were identified, eight of which were case series (level 4 data). Three comparative studies examined the difference between closure and non-closure of the fascial defect during laparoscopic ventral incisional hernia repairs (level 3 and 4 data).

These studies suggested that primary fascial closure (*n* = 138) compared to non-closure (*n* = 255) resulted in lower recurrence rates (0–5.7 vs. 4.8–16.7%) and seroma formation rates (5.6–11.4 vs. 4.3–27.8%).

It showed better outcomes with primary fascial closure. Closure of the central defect during LVHR resulted in less recurrence, bulging, and seroma than non-closure. Patients with closure were more satisfied with the results and had better functional status. The quality of the data was poor.

**Mitura et al**. [10] compares the outcomes of bridging (IPOM) and augmentation (IPOM-Plus) in laparoscopic midline hernia repairs. Conclusions: Laparoscopic ventral hernia repair is generally safe and is associated with the low recurrence rate. Closure of fascial defects before mesh insertion offers better treatment outcomes. Non-closure of fascial defects with only bridging of the hernia defect using standard laparoscopic intraperitoneal onlay mesh repair (IPOM) causes more frequent recurrence and bulging. As a result, patient satisfaction with treatment is lower, and they are concerned about hernia recurrence.

82 hernia repairs using laparoscopic technique with Physiomesh. Patients were divided into IPOM and IPOM-plus groups. After 12 months, eight patients (20%) in IPOM group reported subjectively perceived recurrence and none in IPOM-plus group (*p* = 0.002). Six patients (14.3%) in IPOM group reported suspected recurrence, as compared to three patients (7.1%) in IPOM-plus group (*p* = 0.13). Eventually, four cases of hernia recurrence were confirmed in IPOM group (10%) and none in IPOM-plus group (*p* = 0.018). Other patients presented with mesh bulging.

In the non-fascial closure group *n* = 38, by **Light D, Bawa S.** [19], five patients developed a seroma (12%). One patient developed a wound infection (3%). Six patients presented with a recurrence over the study period (15%). In the fascial closure group (*n* = 74), four patients had a seroma, which was managed conservatively (5%). One patient developed a wound infection (1%). Five patients developed a recurrence over the study period (7%). Conclusion: We have shown comparable rates for seroma and recurrence to other series. Laparoscopic incisional hernia repair with defect closure is feasible and reduces seroma rate and recurrence.

**Limited confirmation**

**Papageorge et al**. [12] have failed to demonstrate benefit of PFC (primary fascial closure) in preventing 30-day postoperative wound morbidity.

The study included 1280 patients, 69% (*n* = 887) underwent PFC. The primary outcome was seroma formation, diagnosed either clinically or radiographically. Secondary outcomes included surgical site infections (SSI), surgical site occurrences (SSO), and SSO requiring intervention. PFC does not decrease the risk of short-term wound complications. Recurrence rates of both groups are not commented.

**Wennergren JE et al**. [13] published in 2016 a comparative study of primary fascial closure versus bridged repair in laparoscopic ventral hernia repair. Conclusion: Primary fascial closure during laparoscopic hernia repairs did not result in reduced recurrence, seroma, and SSI as compared to bridge repairs in a retrospective multi-institutional study. Despite the conclusion on seroma, there were more seromas in the non-closure group!

**Lambrecht JR et al.** [14] reported 37 patients with PH (primary hernia) and 70 with IH (incisional hernia) which were enrolled in a prospective cohort study, treated with laparoscopic ventral hernia repair (LVHR) and randomized to ±transfascial sutures.

Closure of the hernia defect did not influence rate of seroma, pain at 2 months, protrusion, or recurrence. An overall increased complication rate was seen after defect closure (OR 3.42; CI 1.25–9.33). Defect closure (raphe), when using absorbable suture, did not benefit long-term outcomes and caused a higher overall complication rate.

**Ongoing studies**

**Liang MK.** Primary fascial closure with laparoscopic ventral hernia repair: A randomized controlled trial. 2015. https://clinicaltrials.gov/ct2/show/NCT02363790?term = liang + hernia&rank = 1#wrapper (Last accessed September 25, 2017 This study is ongoing, but not recruiting participants.)

**Muysoms, Berrevoet, Tollens et al**. The Influence of closing the gap on postoperative seroma and recurrences in laparoscopic ventral hernia repair (CLOSURE). 2012. https://clinicaltrials.gov/ct2/show/NCT01719718?term = laparoscopic + hernia&rank = 65 (Last accessed October 6, 2017. The recruitment status of this study is unknown. The completion date has passed and the status has not been verified in more than 2 years.)

**Stabilini C, Bracale U, Pignata G, Frascio M, Casaccia M, Pelosi P, Signori A, Testa T, Rosa GM, Morelli N, Fornaro R, Palombo D, Perotti S, Bruno MS, Imperatore M, Righetti C, Pezzato S, Lazzara F, Gianetta E.** Laparoscopic bridging vs. anatomic open reconstruction for midline abdominal hernia mesh repair [LABOR]: single-blinded, multicenter, randomized, controlled trial on long-term functional results. Trials. 2013 Oct 28;14:357. 10.1186/1745-6215-14-357.

**Christoffersen MW.** The effect of laparoscopically closing the hernia defect in laparoscopic ventral hernia repair on postoperative pain (CLOSE-GAP). 2013. https://clinicaltrials.gov/ct2/show/NCT01962480?term = fascial + and + closure&rank = 34. (Last accessed September 25, 2017. This study is enrolling participants by invitation only.)

**Interesting, but low level of evidence or not exact topic**

**Lyons et al. [**16] used a porcine model to show that barbed suture requires the application of 75% less force than conventional suture, while maintaining adequate mesh fixation strength.

**Vorst et al**. [17] state in their “Evolution and advances in laparoscopic ventral and incisional hernia repair” (2015) that re-approximating the abdominal fascia is thought to be a more physiologic repair, and thus stronger. Additionally, it provides a greater surface area of abdominal wall for the mesh to be in contact with. Furthermore, it prevents postoperative bulging of the mesh into the defect.

Conversely, closing the defect increases tension, which may be counterproductive. Also, placement of extra suture in the abdominal wall increases the risk of postoperative pain.

**Awaiz A et al.** [18] concludes in their meta-analysis and systematic review (2015) that laparoscopic and open repair of incisional hernia is comparable. A larger randomized controlled multicenter trial with strict inclusion and exclusion criteria and standardized techniques for both repairs is required to demonstrate the superiority of one technique over the other. Six RCTs, four of them old, two of 2013.

**References** (in parenthesis the level of evidence)

1. Nguyen DH, Nguyen MT, Askenasy EP, Kao LS, Liang MK (2014) Primary fascial closure with laparoscopic ventral hernia repair: Systematic review. World J Surg 38:3097–3104. (1A)
2. Clapp ML, Hicks SC, Awad SS, Liang MK (2013) Trans-cutaneous Closure of Central Defects (TCCD) in laparoscopic ventral hernia repairs (LVHR). World J Surg; 37: 42-51 [PMID: 23052806 10.1007/s00268-012-1810-y] (2C)
3. Zeichen MS, Lujan HJ, Mata WN, Maciel VH, Lee D, Jorge I, Plasencia G, Gomez E, Hernandez AM (2013) Closure versus non-closure of hernia defect during laparoscopic ventral hernia repair with mesh. Hernia 17: 589-596 [PMID: 23784711 10.1007/s10029-013-1115-6] (3)
4. Banerjee A, Beck C, Narula VK et al. (2012) Laparoscopic ventral hernia repair: does primary repair in addition to placement of mesh decrease recurrence? Surg Endosc 26:1264–1268. 10.1007/s00464-011-2024-3 (2C)
5. Tandon A, Pathak S, Lyons NJ, Nunes QM, Daniels IR, Smart NJ (2016) Meta-analysis of closure of the fascial defect during laparoscopic incisional and ventral hernia repair. Br J Surg 103(12):1598-1607. 10.1002/bjs.10268. Epub 2016 Aug 22. (1A)
6. Holihan JL, Askenasy EP, Greenberg JA, Keith JN, Martindale RG, Roth JS, Mo J, Ko TC, Kao LS, Liang MK (2016) Ventral Hernia Outcome Collaboration Writing Group. Component Separation vs. Bridged Repair for Large Ventral Hernias: A Multi-Institutional Risk-Adjusted Comparison, Systematic Review, and Meta-Analysis. Surg Infect (Larchmt) 17(1):17-26. 10.1089/sur.2015.124. Epub 2015 Sep 16. Review. (1A)
7. Rea R, Falco P, Izzo D, Leongito M, Amato B (2012) Laparocopic ventral hernia repair with primary transparietal closure of the hernial defect. BMC Surg 12(Suppl 1):S33 (4)
8. Booth JH, Garvey PB, Baumann DP, Selber JC, Nguyen AT, Clemens MW, Liu J, Butler CE (2013) Primary fascial closure with mesh reinforcement is superior to bridged mesh repair for abdominal wall reconstruction. J Am Coll Surg 217(6):999-1009. 10.1016/j.jamcollsurg.2013.08.015. Epub 2013 Sep 29. (3)
9. Suwa K, Okamoto T, Yanaga K (2016) Closure versus non-closure of fascial defects in laparoscopic ventral and incisional hernia repairs: a review of the literature. Surg Today 46(7):764-73. 10.1007/s00595-015-1219-y. Epub 2015 Jul 22. Review. (2A)
10. Mitura K, Skolimowska-Rzewuska M, Garnysz K (2017) Outcomes of bridging versus mesh augmentation in laparoscopic repair of small and medium midline ventral hernias Surg Endosc 31:382–388 10.1007/s00464-016-4984-9 (2C)
11. Chelala E, Barake H, Estievenart J, Dessily M, Charara F, Alle JL (2016) Long-term outcomes of 1326 laparoscopic incisional and ventral hernia repair with the routine suturing concept: a single institution experience. Hernia 20: 101–110. (2C)
12. Sharma D, Jindal V, Pathania OP, Thomas S (2010) Novel technique for closure of defect in laparoscopic ventral hernia repair. J Minim Access Surg 6: 86–88. (4)
13. Papageorge CM, Funk LM, Poulose BK, Phillips S, Rosen MJ, Greenberg JA (2017) Primary fascial closure during laparoscopic ventral hernia repair does not reduce 30-day wound complications. Surg Endosc 31(11):4551-4557. 10.1007/s00464-017-5515-z. (3B)
14. Wennergren JE, Askenasy EP, Greenberg JA, Holihan J, Keith J, Liang MK, Martindale RG, Trott S, Plymale M, Roth JS (2016) Laparoscopic ventral hernia repair with primary fascial closure versus bridged repair: a risk-adjusted comparative study. Surg Endosc 30:3231–3238 10.1007/s00464-015-4644-5 (3A)
15. Lambrecht JR, Vaktskjold A, Trondsen E, Øyen OM, Reiertsen O (2015) Laparoscopic ventral hernia repair: outcomes in primary versus incisional hernias: no effect of defect closure. Hernia 19(3):479-86. 10.1007/s10029-015-1345-x. Epub 2015 Feb 7. (3)
16. Lyons C, Joseph R, Salas N, Reardon PR, Bass BL, Dunkin BJ (2012) Mesh fixation with a barbed anchor suture results in significantly less strangulation of the abdominal wall. Surg Endosc 26: 1254-1257 [PMID: 22083327 10.1007/s00464-011-2014-5] (4)
17. Vorst AL, Kaoutzanis C, Carbonell AM, Franz MG (2015) Evolution and advances in laparoscopic ventral and incisional hernia repair World J Gastrointest Surg 7(11):293-305. 10.4240/wjgs.v7.i11.293. Review. (4)
18. Awaiz A, Rahman F, Hossain MB, Yunus RM, Khan S, Memon B, Memon MA. (2015)Meta-analysis and systematic review of laparoscopic versus open mesh repair for elective incisional hernia. Hernia 19(3):449-63. 10.1007/s10029-015-1351-z. Epub 2015 Feb 4. Review. (1A)
19. Light D, Bawa S (2016) Trans-fascial closure in laparoscopic ventral hernia repair. Surg Endosc 30:5228–5231 (3)

## Chapter 14. How much overlap is necessary?

### A. De Beaux, S Morales-Conde

**Key questions:**

1. Is there an optimal relation between the size/area of the defect and the size/area of the mesh regarding recurrence rate?
2. Should the overlap always be the same independent on the size of the defect?

**Search terms**

A Medline search using the previous search terms failed to produce any new papers of relevance to this topic. A Medline search on “laparoscopic repair” and “ventral hernia” identified a meta-analysis published in 2016 on the subject [LeBlanc 2016]. All titles and abstracts of papers published since this meta-analysis were reviewed, and papers of interest identified. The reference list of these papers was also reviewed to identify any further papers relevant to the topic. For the study of the original guidelines, read the publication in “Surg Endosc (2014) 28: page 22-23.”

**New publications**

An updated meta-analysis related to the key question and two further cohort studies were identified.

**New statements**

**Table:** 

Level 3	Increasing hernia defect size, and reducing overlap size, among other factors was related to the risk of hernia recurrence
Level 3	The mesh area-to-defect area ratio appears to be more important to minimize recurrence, than a single-mesh overlap length (such as 5 cm)

**New recommendations**

**Table:** 

Grade C	The mesh area-to-defect area ratio should be at least 16:1. In other words, the radius of the mesh used should be at least four times the radius of the defect
Grade C	The use of a rule such as a 5-cm mesh overlap for all hernia defects should be abandoned
Grade C	As the defect size increases, the size of the mesh overlap should also increase

**Comments**

A meta-analysis (1) of mesh overlap following laparoscopic ventral hernia, identified studies, grouped by mesh overlap less than 3 cm, 3–5 cm, and over 5 cm. Only 3 studies (1 published since 2012) were included in the last category. The pooled estimate of risk based on the extent of mesh overlap was 8.6, 4.6, and 1.4%, respectively, for the 3 categories given above. In the studies included for this meta-analysis, there were not enough studies to allow the comparison of hernia defect size to mesh overlap size. However, results from the limited dataset available suggested that the recurrence rate dropped with increasing area of mesh overlap for defects of all sizes. However, the meta-analysis commented that none of the studies were primarily set up to evaluate the extent of mesh overlap as a risk factor for recurrence. Heterogeneity between the studies was high; follow-up time was very short—in some studies less than a month; definition of assessment of recurrence was variable; and 50% of studies were retrospective cohorts. Since this meta-analysis was published, one further cohort study (2) has reported that increasing hernia defect size, and reducing overlap size, among other factors was related to the risk of hernia recurrence. 185 laparoscopic ventral hernia repairs with 13 (7%) recurrence rate was reported.

A theoretical relationship between the area of the hernia defect and the area of the mesh was proposed by Tulloh and de Beaux [3], with a ratio of 1:16 for area, or 1:4 for radius of defect to radius of mesh. This theory was tested in a prospective series of laparoscopic ventral hernia repair using a bridging technique [4]. 213 consecutive patients were followed up for a mean of 69 months, with 16 (7.5%) patients identified to have a recurrence hernia. On multivariate analysis (incorporating a number of risk factors identified from univariate analysis), the mesh:defect area ratio was the only significant risk factor for hernia recurrence. With a mesh:defect ratio of 8 or less, between 9 and 12, between 13 and 16, and 17 or over, the recurrence rate was 70, 35, 9, and 0%, respectively.

The effect on closure of the defect on mesh size, the landing surface of the mesh, mesh type, and mesh fixation method of the mesh may all affect the size of the mesh required to minimize the risk of recurrence. However, it is unlikely that such factors will change the new concept that the area of the mesh covering the area of the hernia defect maybe a more useful tool to calculate mesh size than a length of mesh overlap, such as 5 cm. However, more studies are required to identify the appropriate ratios to cover the various hernia locations in differing patient populations, mesh types, and fixation methods on an appropriately prepared landing surface.

**References** (in parenthesis the level of evidence)

1. LeBlanc K (2016) Proper mesh overlap is a key determinant in hernia recurrence following laparoscopic ventral and incisional hernia repair. Hernia 20:85-99. 10.1007/s10029-015-1399-9 (2A)
2. Nardi M, Millo P, Brachet Contul R, Lorusso R, Usai A, Grivon M, Persico F, Ponte E, Bocchia P, Razzi S (2017) Laparoscopic ventral hernia repair with composite mesh: analysis of risk factors for recurrence in 185 patients with 5 years follow up. Int J Surg 40:38-44. 10.1016/j.ijsu.2017.02.016 (3)
3. Tulloh B, de Beaux A (2016) Defects and donuts:the importance of the mesh:defect area ratio. Hernia 20:893-5. 10.1007/s10029-016-1524-4 (4)
4. Hauters P, Desmet J, Gherardi D, Dewaele S, Poilvache H, Malvaux P (2017) Assessment of predictive factors for recurrence in laparoscopic ventral hernia repair using a bridging technique. Surg Endosc Sept 31 (9):3656-3663. 10.1007/s00464-016-5401-0 (3)

## Chapter 15/16. Fixation

### R. Fortelny, M.C. Misra, V.K.Bansal, F. Köckerling, L. Jorgensen, J. Kukleta

**Key questions:**

1. In terms of recurrence rate, what is better absorbable or permanent fixation?
2. In terms of recurrence rate, what is better tack fixation or transfascial suture fixation or a combination of both?
3. In terms of pain and quality of life, what is better absorbable or permanent fixation?
4. In terms of pain, what is better tack fixation or transfascial suture fixation or a combination of both?
5. In terms of recurrence rate and chronic pain, are self-adhesive meshes better than tack or suture fixation?
6. In terms of costs and hospital stay, what is better non-absorbable tack fixation or absorbable tack fixation?

**Search terms:**

“laparoscopic hernia repair” AND “LVHR” AND “incisional hernia” AND “suprapubic hernia” AND “parapubic hernia” AND “subxiphoidal hernia” AND “fixation” AND “tacks” AND “staples” AND “recurrences” AND “pain” AND “long term results”

In September 2017, a systematic search of the available literature published after August 2011 was performed using Medline, PubMed, and the Cochrane Library, as well as a search of relevant journals and reference lists. After selection of 135 papers concerning the suitability of the different topics in total, 16 new articles including 3 reviews, 7 RCT, 1 cohort study, 3 retrospective study, and 2 CCS were included in this review.

For the study of the original guidelines, read the publication in “Surg Endosc (2014) 28: page 23-28.”

**Recurrence**

**New statements:**

**Table:** 

Level 1A	The risk of hernia recurrence after tacker or suture fixation is similar
Level 1B	The use of glue only fixation is associated with an increased risk for recurrence
Level 2C	The use of absorbable tacks may be a risk factor for recurrence compared to non-absorbable tacks

**New Recommendations:**

**Table:** 

Grade A	Suture fixation alone or in combination with tacks or double-crown tacker fixation alone is recommended to minimize the risk of hernia recurrence

**Pain**

**New statements:**

**Table:** 

Level 1B	Combined fixation with tacks and transfascial sutures causes more pain compared to double-crown tack fixation in the first 3 months
	There is no difference in postoperative pain between absorbable and non-absorbable tack fixation

**New recommendations:**

**Table:** 

Grade B	The use of non-absorbable or absorbable tacks is equally recommended in terms of postoperative pain

**Quality of Life**

**New statements:**

**Table:** 

Level 1 B	The use of non-absorbable or absorbable tacks is equally recommended in terms of postoperative pain

**New recommendations:**

**Table:** 

Grade B	Fixation by absorbable and non-absorbable tacks is equally recommended in terms of QoL.

**Costs**

**New statements:**

**Table:** 

Level 1B	Non-absorbable tack fixation is cheaper in comparison to absorbable tack fixation

New Recommendations:

**Table:** 

Grade B	Non-absorbable tack fixation should be preferred to absorbable tack fixation in terms of cost

**Hospital stay**

**New statements:**

**Table:** 

Level 1A	The length of hospital stay after tacker and/or suture mesh fixation is similar
Level 1B	The length of hospital stay after absorbable or non-absorbable tack fixation is similar

**New recommendations:**

**Table:** 

Grade A	Different tacker/suture mesh fixation techniques are equally recommended in terms of hospital stay

**Recurrence and pain**

**Suture versus tacker fixation**

Two RCT (LoE 1B) studies [11, 12], 1 retrospective (LoE 2B) study [8], and 2 (LoE 1A) reviews [3, 4] were published comparing suture and tacker fixation that were relevant for the update of the guidelines regarding recurrence and pain.

The randomized controlled trial of Bansal et al. [11] included a total of 110 patients and compared suture fixation of the mesh in a distance of 1–2 cm on the borders by using a non-absorbable suture material (55 patients) with a tacker fixation by metallic tacker in double-crown technique (55 patients). The postoperative pain up to 1 month (1 h, 6 h 24 h, 1 week) was significantly lower (*p* < 0.001) in the suture group and the return to daily activities was significantly shorter in the suture group (< 0.01). No significant differences were found concerning chronic pain and recurrences in a mean follow-up of 32.2 months.

The comparison of the fixation time was significantly in favor of the tacker group (18.8 min. vs 39.1 min.; *p* < 0.001).

Grubnik et al. [12] published a RCT in 2012 enrolling 92 patients with randomized allocation of 43 to tacker fixation using a ePTFE mesh and 49 to suture fixation using self-expanding double-layer mesh, respectively. The mean fixation time for metal tacker in double-crown technique was significantly longer in comparison to the suture fixation by non-absorbable material, which was placed predominantly only at the four corners (*p* < 0.01). The postoperative stay for the suture group was significant shorter (*p* < 0.01) compared to the tacker group. In the follow-up of mean 13.7 months, the rate of seroma (7 vs 1), hematoma (8 vs 0), and recurrences (4 vs 1) was significantly increased in the suture group.

**Critical comment:** The use of only 4 sutures for fixation is unusual especially in terms of fixation times compared to double-crown fixation. Furthermore, there is also lack of description of the metal tacker type. In case of the use of Endopath EMS^®^ stapler, the minimal penetration depth could be the reason for the high recurrence rate. Another problem arises with the statement of fixation time and operative time, which are described as identical. Another detectable risk of bias is the use of two different types of meshes (ePTFE only and combination of ePTFE with a nitinol frame) in combination with two different fixation techniques. In summary, this paper has some crucial shortcomings, and conclusions should be interpreted in light of this methodological short coming.

Another study comparing suture versus tack fixation in a retrospective analysis of 86 patients (33 suture versus 53 tacks group) was published by Kitamura et al. [8]. In a mean follow-up of 2.7 years (90% in suture group and 58% in the tacker group), no significant difference was detected (3 vs 2) using telephone interview (48% of suture group and 57% of tacks group). No significant difference in postoperative pain was observed.

**Critical comment:** Emergency cases were included only in the tack group (6 vs 0). Low number of patients enrolled. In addition, different types of meshes were used without selective analysis and only 58% of the tack group was available for follow-up.

2 Reviews – Level IA

In the meta-analysis of Sajid et al. [3] comparing tacker fixation with suture fixation, 2 randomized controlled trials of level of evidence 1B (Bansal et al. 2011; Beldi 2011) and 2 non-randomized studies (Greenstein 2008 and Nguyen 2008) were enrolled. The operation time of the 99 patients treated by tacker fixation compared to 108 patients by suture fixation was significant shorter (MD, − 23.65; 95% CI − 31.06, − 16.25; *z* = 6.26; *p* < 0.00001). In the four to six-week follow-up, the postoperative pain score in the tacker group was significantly lower (MD, − 0.69; 95% CI − 1.16, − 0.23; *z* = 2.92; *p* < 0.004). The perioperative complication rate (*p* = 0.65), length of hospital stay (*p* = 1), and risk of hernia recurrence (OR 1.54; 95% CI 0.38, 6.27; *z* = 0.61; *p* = 0.54) after tacker and suture fixation did not reveal any statistical difference.

**Critical comment:**

The result of postoperative pain in favor for suture fixation is based only on 3 studies (2 RCT and 1 non-RCT).

In the review published by Reynvoet et al. [4] comparing fixation methods in laparoscopic ventral hernia repair, a total of 25 series (13 trials used both tacks and sutures, 10 used only tacks, and 2 used only sutures) were included for statistical evaluation. The overall recurrence rate was 2.7% (95% CI [1.9–3.4%]) without statistical difference between the fixation techniques. The test for heterogeneity of the included studies was not statistically significant (p > 0.1). This review reported the postoperative pain in an overview but without statistical analysis.

**Critical comment:**

This review was based on a high number of studies, but the only conclusion out of the analysis was that there is no difference of outcome regarding the recurrence rates.

**Suture and tacker versus double-crown tacker fixation**

Only 1 randomized study comparing ‘suture + tacker’ versus tacker fixation alone was published by Muysoms et al. [9] relevant for this update. In total, 76 patients were included and allocated randomly to both groups (43 sut. + tack. versus 33 tacker alone). The primary endpoint of pain assessed by VAS 4 h postoperatively and after 3 month of follow-up obtained significantly less favorable results for the double-crown tacker group (4 h postop. in rest: 0.013 versus 0.028 and after 3 months—VAS ≥ 1.0 cm: 8.3% vs. 31.4%). The operation time was significantly shorter for the double-crown fixation (74 vs 96 min.; *p* = 0.014). The second endpoint defined as recurrence rate after 24 months was without significant difference (11.1. vs 3.7%; *p* = 0.83).

**Critical comment:**

The estimated sample size of 270 patients was changed to 220 patients and finally due to lack of enrollment only 34.5% of the aimed sample size was achieved. Therefore, the statistical significance and the results might be unsound. In addition, the patients were not blinded and only 82.9% of patients were available for the follow-up.

The results of this RCT in comparison to the RCT of Wassenaar et al. [17] are quite different. Muysoms et al. argued the difference might be based on the inclusion of different patient cohorts—Wassenaar recruited smaller defect size cases and less incisional hernia in comparison to the patients of Muysoms—this might be of importance based on the fact that the number of suture fixation is associated with higher pain scores (Carbonell et al. [18]).

**Comparison of two different mesh and resorbable fixation devices**

Pawlak et al. [10] published a single-center, prospective randomized study comparing mesh and dedicated fixation systems (group I: Physiomesh^®^/Securestrap^®^ and group II Ventralight ST^®^/SorbaFix^®^) with inclusion of two groups of patients (50 per group planned). Due the significant results of a planned interim analysis after 6 months which detected 20% of recurrences in group I versus 0% in group II group, the study was stopped due to safety reasons. The postoperative pain in group I revealed significantly higher pain after 3 months (*p* < 0.0001).

**Fibrin glue fixation versus tacker fixation in umbilical hernia**

The one-year results of the RCT in umbilical hernia with defect sizes of 1.5 to 5 cm comparing mesh fixation by fibrin glue versus tacker published by Eriksen et al. [13] reported a recurrence rate of 26% (6 patients) in the glue group versus 6% (1 patient) in the tack group (*p* = 0.182). The sub-analysis detected a significant association between recurrence and defect size of 4.0.cm and 2.8 cm (*p* = 0.009). A 12-cm mesh was used in all cases regardless of defect size, leaving overlap between 3.5 cm and 5 cm, which may have significantly impacted the outcomes. The results of SF 36 as well as pain, discomfort, and fatigue were similar in both after 1 year.

**Non-absorbable versus absorbable tacker**

The comparison of fixation by non-absorbable versus absorbable tacker was the focus in 2 (LoE 1B) RCTs [6, 7], 1 registry study (LoE2C), and 2 (LoE 2B) retrospective studies.

Colak et al. [7] performed a prospective randomized trial comparing non-absorbable with absorbable tacks in laparoscopic incisional hernia repair in terms of pain using VAS and recurrence in median follow-up of 31 months. In both groups (non-absorbable, *n* = 25; absorbable group *n* = 26), the tacks were placed in a single crown technique at a distance of 1.5–2.0 cm and a mesh overlap of at least 5 cm. After a median follow-up time of 31 months, recurrence of 7.8% in each group (2 patients) was observed. With regard to operation time, hospital stay, morbidity, and postoperative pain (VAS at 1 day, 2 weeks, and six-month postop.), no significant differences between both groups were found.

**Critical comment:** Study is likely underpowered to truly confirm or refute the stated hypothesis.

Another randomized controlled trial comparing non-absorbable versus absorbable tacker was published by Bansal et al. [6] in 2016. 90 patients were admitted for LIVHR repair (defect size < 15 cm) and randomized into 2 groups including 45 patients each. Incidence of immediate postoperative and chronic pain, as well as postoperative quality of life outcomes, and patient satisfaction scores after a mean follow-up of 8.8 months showed no significant differences between the groups. The cost of the procedure was significantly higher in the absorbable tacker group (P < 0.01).

A prospective, nationwide, registry-based study regarding the long-term risk of recurrence (clinical/radiological recurrence or reoperation) and chronic pain in patients undergoing primary, elective, laparoscopic incisional hernia repair with absorbable or non-absorbable tack fixation performed by Christoffersen et al. [5] was published 2015. 816 patients were included for analysis. After a median observation time of 40 months, the cumulative recurrence-free survival rate was 71.5 and 82.0% after absorbable and non-absorbable tack fixation, respectively (*p* = 0.007). The use of absorbable tacks was an independent risk factor for recurrence in a multivariable analysis (hazard ratio 1.53, 95% CI 1·11 to 2·09; *p* = 0.008). The rate of moderate or severe chronic pain was without significant difference between the groups (*p* = 0.765).

**Critical comment:** The hernia size in the non-absorbable fixation group was significantly larger (7 versus 9 cm; *p* < 0.001) and the follow-up for the absorbable tacker group was significantly shorter (34 versus 44 month). There is a lack of information regarding the type, number, and placement (e.g., single or double crown, use of a central suture for mesh positioning) of absorbable tacker used, as well as the size of overlap of the mesh. These shortcomings may potentially influence the results of this analysis. Furthermore, this outcome study differs mainly to that of the RCTs of Colak et al. and Bansal et al.

A small retrospective analysis of 40 laparoscopic incisional hernia repairs in 38 patients comparing titanium tacks versus absorbable tacks was published by Cavallaro et al. [1]. The 24-h postoperative pain (VNS) was significant higher in the group of titanium tacks (*p* < 0.05), but no differences in recurrence rates in mean follow-up of 14.6 and 10.7 months for the titanium tack and absorbable tack group, respectively, were detected.

Caruso et al. [2] reported in a retrospective study comparing non-absorbable and absorbable fixation devices (EMS^®^ in 260 patients, ProTack^®^ in 210 patients, and AbsorbaTack^®^ in 30 patients), a significant higher recurrence rate (10%) after AbsorbaTack^®^ fixation in a mean follow-up of 19 months. Postoperative neuralgia was detected in 3.8% after ProTack^®^ fixation versus 0.7% after EMS^®^ fixation and 0% after AbsorbaTack^®^ fixation.

**Quality of life**

In terms of QoL, 2 (LoE) RCTs by Bansal et al. [6, 11] were included in this update.

In the RCT of Bansal et al. [11] comparing the long-term outcome and quality of life after laparoscopic repair of incisional and ventral hernias with suture fixation with and without tacks, no difference in improvement in scores from preoperative and 3 months postoperatively in any of the domains of WHO-QOL BREF physical, psychological, social, and environmental was detected. There was also no difference in how patients perceived their overall QoL and their overall satisfaction at 3 months postoperatively in both the groups.

In another RCT of Bansal et al. [6] comparing absorbable and non-absorbable tacks, postoperative QoL and patient satisfaction scores did not show any difference.

**Costs**

Regarding the update of costs, 2 (LoE 1B) RCTs [4, 7] were published.

Bansal et al. [4] in their RCT comparing absorbable and non-absorbable tacks have found significantly less cost in non-absorbable tack fixation compared to absorbable tacks. Cost effectiveness was compared by calculation of incremental cost-effectiveness ratio. Non-absorbable tacks were found to be more cost effective.

In the opposite, Colak et al. [7] found in a prospective randomized trial comparing non-absorbable with absorbable tacks in laparoscopic incisional hernia repair that absorbable tacks may be the preferable option due to the lower cost (the price of non-absorbable tacker was $ 325, whereas only $ 190 for absorbable tacker).

**Hospital stay**

The update concerning the hospital stay includes 3 (LoE 1B) RCTs [7, 9, 11, 12] and 2 comparative (LoE 2B) studies.

In the RCT by Muysoms et al. [9] comparing combined suture and tack fixation with double-crown tack fixation, hospital stay was comparable between the two groups. However, Grubnik et al. [12], in a RCT comprising 92 patients, found significant shorter hospital stay in patients undergoing suture fixation of mesh compared to tack fixation (2.5 days versus 4.5 days). A previous study by Bansal et al. [11] showed no difference in hospital stay between suture and tack fixation. Kitamura et al. [8] also did not find any difference in hospital stay in a comparative study between suture and tack fixation. Colak et al. [7], in a RCT comprising 51 patients, found comparable hospital stay, operative time, postoperative pain scores, morbidity, and recurrence in patients undergoing mesh fixation with absorbable and non-absorbable tacks. In a comparative study, Cavallaro et al. [1] have found significant lower postoperative pain scores and a shorter hospital stay in patients undergoing fixation with absorbable tacks.

**Subxiphoidal hernia**

**Key questions:**

1. Is the complete detachment of the falciform (lg. teres hepatis) ligament necessary to decrease recurrence?
2. What is the optimal fixation to the pubic bone?
3. What is the optimal fixation for subxiphoid hernia repair and to the diaphragm?

**New statements:**

**Table:** 

Level 4	The use of penetrating fixation devices (tacks and sutures) above the costal margin is associated with the risk of pain and pericardial injuries

**New recommendations:**

**Table:** 

Grade D	The use of penetrating fixation devices (tacks and sutures) is not recommended above the costal margin
	Above the costal margin, only non-penetrating fixation devices (e.g., glues) are recommended

**1 Review**

Hope et al. [14] published a review regarding atypical hernias including suprapubic and subxiphoid and flank hernias in 2013. Based on the existing literature, the summarized recommendations are stated in this review as follows:

**Table:** 

Grade B	Tacks should be placed at the costal margin, whereas additional transfascial suture fixation should be placed just below the costal margin
	For mesh fixation above, the costal margin and xiphoid glue should be used.

1 Case–control study

Ghanem et al. [15] reported their experience in a case study of 4 patients treated by laparoscopic repair and recommended to dissect the falciform ligament superiorly toward the diaphragm, as well as to use multiple non-absorbable intracorporeal sutures to anchor the mesh to the diaphragm above the costal margins. Below the costal margin, transfascial non-absorbable sutures and tacks were used to fix the mesh to the anterior abdominal wall

**Suprapubic Hernia**

New statements:

**Table:** 

Level 4	Additional mesh fixation by bone anchors in complex suprapubic hernias is associated with a low recurrence rate

**New recommendations:**

**Table:** 

Grade D	Additional mesh fixation by the use of bone anchors is suggested in complex cases

**1 Review**

Hope et al. [14] reported in this review that the most common techniques in the literature is a combination of suture and tack fixation and the double-crown technique. The risk of recurrence is most likely found in the inferior/pubic region. Additional suture fixation may reduce this complication

1 Case–control study

Yee et al. [16] published a study with bone anchor fixation in complex laparoscopic ventral hernia repair. In 17 suprapubic and 13 lateral hernias, the mesh fixation was performed by the use of these anchors (mean of 2.8 and 3.2 bone anchors). In a follow-up of 13.2 months, 23.3% postoperative complications and 2 recurrences (6.7%) were described.

**References** (in parentheses graduation of evidence)

Cavallaro G, Campanile FC, Rizzello M, Greco F, Iorio O, Iossa A, Silecchia G (2013) Lightweight polypropylene mesh fixation in laparoscopic incisional hernia repair. Minim Invasive Ther Allied Technol 22(5):283–7. 10.3109/13645706.2013.808228. Epub 2013 Jun 30. PubMed PMID: 23808370 (2B)
Caruso F, Ciccarese F, Cesana G, Uccelli M, Castello G, Olmi S (2017) Massive Incisional Hernia Repair with Parietex: Monocentric Analysis on 500 Cases Treated with a Laparoscopic Approach. J Laparoendosc Adv Surg Tech A 27(4):388–392. 10.1089/lap.2016.0623. Epub 2017 Mar 1. PubMed PMID:28249126. (2B)
Sajid MS, Parampalli U, McFall MR (2013) A meta-analysis comparing tacker mesh fixation with suture mesh fixation in laparoscopic incisional and ventral hernia repair. Hernia 17(2):159–66. 10.1007/s10029-012-1017-z. Epub 2012 Nov 9. PubMed PMID: 23138861. (1A)
Reynvoet E, Deschepper E, Rogiers X, Troisi R, Berrevoet F (2014) Laparoscopic ventral hernia repair: is there an optimal mesh fixation technique? A systematic review. Langenbecks Arch Surg 399(1):55–63. Review. PubMed PMID: 24121735. (1A)
Christoffersen MW, Brandt E, Helgstrand F, Westen M, Rosenberg J, Kehlet H, Strandfelt P, Bisgaard T (2015) Recurrence rate after absorbable tack fixation of mesh in laparoscopic incisional hernia repair. Br J Surg 102(5):541–7. 10.1002/bjs.9750. Epub 2015 Feb 19. PubMed PMID: 25703637. (2C)
Bansal VK, Asuri K, Panaiyadiyan S, Kumar S, Subramaniam R, Ramachandran R, Sagar R, Misra MC (2016) Comparison of Absorbable Versus Nonabsorbable Tackers in Terms of Long-term Outcomes, Chronic Pain, and Quality of Life After Laparoscopic Incisional Hernia Repair: A Randomized Study. Surg Laparosc Endosc Percutan Tech 26(6):476–483. PubMed PMID: 27846175. (1B)
Colak E, Ozlem N, Kucuk GO, Aktimur R, Kesmer S, Yildirim K (2015) Prospective randomized trial of mesh fixation with absorbable versus nonabsorbable tacker in laparoscopic ventral incisional hernia repair. Int J Clin Exp Med 15;8(11):21611–6.eCollection 2015. PubMed PMID: 26885113; PubMed Central PMCID: PMC4723958. (1B)
Kitamura RK, Choi J, Lynn E, Divino CM (2013) Suture versus tack fixation of mesh in laparoscopic umbilical hernia repair. JSLS 17(4):560–4. 10.4293/108680813x13693422520044. PubMed PMID: 24398197; PubMed Central PMCID: PMC3866059. (2B)
Muysoms P, Vander Mijnsbrugge G, Pletinckx P, Boldo E, Jacobs I, Michiels M, Ceulemans R (2013) Randomized clinical trial of mesh fixation with “double crown” versus “sutures and tackers” in laparoscopic ventral hernia repair. Hernia 17(5):603–12. 10.1007/s10029-013-1084-9. Epub 2013 Apr 2. PubMed PMID: 23546864. (1B)
Pawlak M, Hilgers RD, Bury K, Lehmann A, Owczuk R, Śmietański M (2016) Comparison of two different concepts of mesh and fixation technique in laparoscopic ventral hernia repair: a randomized controlled trial. Surg Endosc 30(3):1188–97. 10.1007/s00464-015-4329-0. Epub 2015 Jul 3. PubMed PMID: 26139491. (1B)
Bansal VK, Misra MC, Babu D, Singhal P, Rao K, Sagar R, Kumar S, Rajeshwari S, Rewari V (2012) Comparison of long-term outcome and quality of life after laparoscopic repair of incisional and ventral hernias with suture fixation with and without tacks: a prospective, randomized, controlled study. Surg Endosc 26(12):3476–85. 10.1007/s00464-012-2390-5. Epub 2012 Jun 23. PubMed PMID: 22729705. (1B)
Grubnik VV, Grubnik AV, Vorotyntseva KO (2014) Laparoscopic repair of incisional and ventral hernias with the new type of meshes: randomized control trial. Wideochir Inne Tech Maloinwazyjne 9(2):145–51. 10.5114/wiitm.2014.41623. Epub 2014 Apr 1. PubMed PMID: 25097679; PubMed Central PMCID: PMC4105668. (1B)
13. Eriksen JR, Bisgaard T, Assaadzadeh S, Jorgensen LN, Rosenberg J. (2013) Fibrin sealant for mesh fixation in laparoscopic umbilical hernia repair: 1-year results of a randomized controlled double-blinded study. Hernia 17(4):511–4. 10.1007/s10029-013-1101-z. Epub 2013 May 9. PubMed PMID: 23657861. (1B)
Hope WW, Hooks WB 3rd. (2013) Atypical hernias: suprapubic, subxiphoid, and flank. Surg Clin North Am 93(5):1135–62. 10.1016/j.suc.2013.06.002. Epub 2013 Jul 29. Review. PubMed PMID: 24035079 (1A)
Ghanem OM, Zahiri HR, Devlin S, Sibia U, Park A, Belyansky I (2016) Laparoscopic Subxiphoid Hernia Repair with Intracorporeal Suturing of Mesh to the Diaphragm as a Means to Decrease Recurrence. J Laparoendosc Adv Surg Tech A 26(2):129–32. 10.1089/lap.2015.0518. Epub 2016 Jan 27. PubMed PMID: 26863296. (4)
Yee JA, Harold KL, Cobb WS, Carbonell AM (2008) Bone anchor mesh fixation for complex laparoscopic ventral hernia repair. Surg Innov 15(4):292–6. 10.1177/1553350608325231. Epub 2008 Oct 22. PubMed PMID: 18945708. (4)
Wassenaar E, Schoenmaeckers E, Raymakers J, van der Palen J, Rakic S (2010) Mesh-fixation method and pain and quality of life after laparoscopic ventral or incisional hernia repair: a randomized trial of three fixation techniques. Surg Endosc 24(6):1296–302. 10.1007/s00464-009-0763-1. Epub 2009 Dec 24. PubMed PMID: 20033726; PubMed Central PMCID: PMC2869434. (1B)
Carbonell AM, Harold KL, Mahmutovic AJ, Hassan R, Matthews BD, Kercher KW, Sing RF, Heniford BT (2003) Local injection for the treatment of suture site pain after laparoscopic ventral hernia repair. Am Surg 69(8):688–91; discussion 691–2. PubMed PMID: 12953827. (4)

## Chapter 17. Mesh insertion

### M. Misra, V. K. Bansal, R. Fortelny

**Questions:**

1. How to avoid contamination of the mesh?
2. Should mesh be inserted through a trocar or the skin?
3. What is the best way to get a large mesh into the abdominal cavity?

**Table:** 

No changes with regard to the original statements and recommendations which are still valid (—see “Surg Endosc (2014) 28: page 28”)	

## Chapter 18. Management of bowel injury during laparoscopic ventral incisional hernia repair

### Vadim Meytes, Kevin Bain, Karl LeBlanc, George Ferzli

**Questions**

1. What are the incidences of bowel injury, and what are the safest techniques for avoiding them?
2. What is the safest management for bowel injury, and do alternatives exist?

**Search terms:**

“laparoscopic ventral hernia repair” AND “enterotomy” AND “mesh”

A systematic search of the literature was performed in June 2017 using Medline, PubMed, Cochrane library, and reference lists for the time period of January 2012 to June 2017 to update the period. Additional 11 articles were found and analyzed. Six additional articles that met criteria were included in this updated review.

For the study of the original guidelines, read the publication in “Surg Endosc (2014) 28: page 353–355.”

After careful analysis of all articles, the statements and recommendations made from the original guidelines remain valid, therefore they are not repeated. The additional literature review supports the Level 2 statement regarding adhesiolysis. Furthermore based off the new data, Grade C recommendations have been expanded to include finishing the hernia repair without massive enteric leakage and interval hernia repair once signs of infection have dissipated.

**Statements**

**Table:** 

Level 2 Adhesiolysis time was a significant and independent predictive factor for enterotomy	

**Recommendations**

**Table:** 

Grade C	In cases of recognized bowel injury, without significant enteric fluid leakage, it is suggested that the enterotomy can be repaired, followed by a mesh repair of the hernia
	If there is a conversion to open surgery to repair the enterotomy, the hernia repair can be accomplished laparoscopically after an interval of 5–7 days if no signs of infection are present

**Introduction**

The first laparoscopic repair of a ventral incisional hernia (LVHR) was reported by LeBlanc and Booth in 1993. Approximately 90,000 ventral incisional hernia repairs are performed in the United States each year. The LVHR procedure continues to gain increasing popularity over open repair. We have been presenting the updated review of literature on this topic since 2012.

Avoiding bowel injury during LVHR is paramount. The management of bowel injury during LVHR remains controversial. In a recent paper by Misiakos et al. [1], mortality reached 40% in patients who had their enterotomy discovered in the postoperative period. Predictably, the small bowel was injured in 92% of the reported cases.

Conversion to laparotomy for bowel injury sustained during LVHR is an option to be considered. Management is best dictated by the extent of injury, contamination, and by the level of the surgeon’s skill and experience. Options include immediate conversion to open surgery to repair the bowel injury and repair of the hernia with or without mesh implantation. If the surgeon is adept at laparoscopic bowel repair and contamination is limited, the injury may be repaired laparoscopically and the LVHR performed immediately. An alternative is to repair the bowel and delay the hernia repair until after a period of inpatient observation and administration of parenteral antibiotics [1]. In 2012, Choi et al. [4] showed that mesh can be used in clean-contaminated and contaminated cases; however, it carried an increased risk of superficial surgical site infections (SSI), deep SSI and organ/space SSI, wound disruption, pneumonia, and sepsis.

In the event of a bowel injury, there are several alternatives to conversion to a laparotomy. In a 2013 Italian review, Cuccurullo et al. [3] reported that in the presence of a recognized lesion of the small intestine during laparoscopy, one can suture repair the injury laparoscopically and successfully complete the planned hernia repair using prosthetic mesh with acceptable results. This was done only in the absence of any significant contamination of enteric contents.

Finally, the use of biologic mesh also has been described as a safe method for the completion of the LVHR in the presence of contamination. Although synthetic mesh generally is preferred over biologic mesh in terms of recurrence prevention, biologic mesh has been used successfully in contaminated and infected fields. In a recent study, Carbonell et al. [7] reported on outcomes of 2 institutions’ experience implanting lightweight polypropylene synthetic mesh in clean-contaminated and contaminated fields. The authors reported that the incidence of surgical site occurrence was 26.2% in clean-contaminated cases and 34% in contaminated cases. The 30-day surgical site infection rate was 7.1% for clean-contaminated cases and 19.0% for contaminated cases. There were a total of 7 recurrences with a mean follow-up of 10.8 ± 9.9 months. In 2015, Rosen et al. [8] looked at the use of biosynthetic absorbable mesh in contaminated ventral hernia repair. In their study, biosynthetic absorbable mesh showed efficacy in terms of long-term recurrence and quality of life for these patients and offered an alternative to biologic and permanent synthetic meshes in these complex situations. The hernia recurrence rate was 17%. The authors did not comment on other postoperative complications.

**References:** (in parenthesis the level of evidence)

1. Misiakos EP, Patapis P, Zavras N, Tzanetis P, Machairas A. (2015) Current Trends in Laparoscopic Ventral Hernia Repair. JSLS 19(3). (1B)
2. Patel PP, Love MW, Ewing JA, Warren JA, Cobb WS, Carbonell AM. (2017) Risks of subsequent abdominal operations after laparoscopic ventral hernia repair. Surg Endosc 31(2):823-828. (3)
3. Cuccurullo, D., Piccoli, M., Agresta, F, Magnone S, Corcione F, Stancanelli V, Melotti G. (2013) Laparoscopic ventral incisional hernia repair: evidence-based guidelines of the first Italian Consensus Conference. Hernia 17: 557- 566. (1B)
4. Choi JJ, Palaniappa NC, Dallas KB, Rudich TB, Colon MJ, Divino CM. (2012) Use of mesh during ventral hernia repair in clean-contaminated and contaminated cases: outcomes of 33,832 cases. Ann Surg 255(1):176-80. (2C)
5. ten Broek RP, Schreinemacher MH, Jilesen AP, Bouvy N, Bleichrodt RP, van Goor H. (2012) Enterotomy risk in abdominal wall repair: a prospective study. Ann Surg 256(2):280-7. (2B)
6. Sharma A, Khullar R, Soni V, Baijal M, Kapahi A, Najma K, Chowbey PK. (2013) Iatrogenic enterotomy in laparoscopic ventral/incisional hernia repair: a single center experience of 2346 patients over 17 years. Hernia 17(5):581-7. (3)
7. Carbonell AM, Criss CN, Cobb WS, Novitsky YW, Rosen MJ (2013) Outcomes of synthetic mesh in contaminated ventral hernia repairs. J Am Coll Surg 217:991–8. (3)
8. Rosen MJ, Carbonell AM, Cobb WS, Cobb WS, Matthews B, Goldblatt MI, Selzer DJ, Poulose BK, Hansson BM, Rosman C, Chao JJ, Jacobsen GR (2017) Multicenter, prospective, longitudinal trial evaluating recurrence, surgical site infection and quality of life after contaminated ventral hernia repair using biosynthetic absorbable mesh: The COBRA study. Ann Surg 265(1):205-211. (2C)

## Chapter 19. Risk factors for infection in laparoscopic incisional/ventral hernia repair

### P. Chowbey, F. Mayer

**Question:**

1. What are the risk factors for infection in laparoscopic ventral hernia repair?

**Search terms**

The following search terms were used: “risk factors for SSI”; “risk factors for infection”; “laparoscopic ventral hernia repair and perioperative risk factors for infection.”

**Search machines**

PubMed, Medline, and the Cochrane Library as well as the reference lists of the included studies were searched for relevant studies.

For the study of the original guidelines, read the publication in “Surg Endosc (2014) 28: page 357 358.”

**New publications**

A total of 5 new studies were identified since the publication of the original guidelines. Statements and recommendations were modified accordingly.

**New statements**

**Table:** 

Level 2	Following evidence-based guidelines and the specialized hernia clinic were associated with lower SSI rates
Level 2	A body mass index ≥ 30 kg/m^2^, smoking, American Society of Anesthesiology (ASA) class 3, open surgical approach, prolonged operative times, and inpatient admission following ventral incisional hernia repairs are significant predictors of postoperative SSIs
Level 2	Obesity and smoking are modifiable risk factors for SSIs after LVHR
Level 2	SSI was more common with open repair in both primary and incisional hernia groups
Level 3	The institution where surgery is performed and the number of prior abdominal operations are factors associated with SSI
Level 3	Postoperative infectious complications are similar between defect closure and non-closure patients

**New recommendation**

**Table:** 

Grade B	In terms of low SSI rates, the surgeons should follow evidence-based guidelines and the patient should be operated in institutions with appropriate expertise

**Comments**

In a two-pronged quality improvement (QI) project, total of 399 patients in the pre-QI period and 390 patients in post-QI period (178 patients in general surgery clinics; 212 patients in the specialty hernia clinic) underwent ventral hernia repair [1]. Patients treated in the post-QI period were less likely to experience an SSI (13.5% vs 1.5%; *p* < 0.001). On subgroup analysis of the post-QI clinics, specialty hernia clinic patients had an even lower risk of SSI than those in the general surgery clinics (1.4 vs 1.7%).

In a study which identified cases of ventral incisional hernia repair from 2009 to 2010 in American College of Surgeons National Surgical Quality Improvement Program database, a total of 28,269 cases were identified. 25,172 cases met the inclusion criteria. The study demonstrated that body mass index ≥ 30 kg per meter square, smoking, American Society of Anaesthesiology (ASA) class 3, open surgical approach, prolonged operative times, and inpatient admission following LVHR were significant predictors of postoperative SSIs [2]. Obesity and smoking are modifiable risk factors [2].

In a systematic review and meta-analysis which included five and fifteen studies for primary and incisional cohorts, SSI was found to be more common with open repair in both hernia groups: primary (OR 4.17, 95% CI [2.03–8.55]) and incisional (OR 5.16, 95% CI [2.79–9.57]) [3].

In a retrospective study of 201 consecutive patients who underwent elective LVHR with prosthetic mesh placement at two institutions from January 2000 to December 2010, it was noted that the only predictor of SSIs was the institution where the operation was performed and the number of prior abdominal operations [4].

In a retrospective review of 176 patients who underwent LVHR between January 2007 and December 2010, postoperative infectious complications and early complications classified by the Dindo–Clavien system were found to be similar between central defect closure and non-closure groups [5].

**References:** (in parenthesis the level of evidence)

1. Cherla DV, Holihan JL, Flores-Gonzalez JR, Lew DF, Escamilla RJ, Ko TC, Kao LS, Liang MK (2017) Decreasing Surgical Site Infections after Ventral Hernia Repair: A Quality-Improvement Initiative. Surg Infect 18(7):780–6. [2]
2. Kaoutzanis C, Leichtle SW, Mouawad NJ, Welch KB, Lampman RM, Wahl WL, Cleary RK (2013) Risk factors for postoperative wound infections and prolonged hospitalization after ventral/incisional hernia repair. Hernia 19(1):113–23. [2]
3. Arita NA, Nguyen MT, Nguyen DH, Berger RL, Lew DF, Suliburk JT, Askenasy EP, Kao LS, Liang MK (2015) Laparoscopic repair reduces incidence of surgical site infections for all ventral hernias. Surg Endosc 29 (7):1769–80. [2]
4. Brahmbhatt R, Carter SA, Hicks SC, Berger DH, Liang MK (2014) Identifying Risk Factors for Surgical Site Complications after Laparoscopic Ventral Hernia Repair: Evaluation of the Ventral Hernia Working Group Grading System. Surg Infect 15(3):187–93. [3]
5. Clapp M, Awad S, Subramanian A, Liang M (2012) Trans-cutaneous Closure of Central Defects (TCCD) in Laparoscopic Ventral Hernia Repairs (LVHR). J Surg Research 172(2):286–7. [3]

## Chapter 20. Mesh Infection

### F. Köckerling, P. Chowbey, A. Sharma

**Key questions:**

1. How should a mesh infection be treated?
2. What is the role of biologic meshes in the treatment of mesh infection?

**Search terms**

The following search terms were used: “incisional hernia”; “ventral hernia”; “laparoscopic ventral hernia repair”; “laparoscopic incisional hernia repair”; “mesh infection”; “Hernia repair and mesh infection.”

**Search machines**

PubMed, Medline, and the Cochran Library as well as the reference lists of the included studies were searched for relevant studies.

**New publications**

A total of 6 new studies were identified since the publication of the original guidelines. Statements and recommendations were modified accordingly. For the study of the original guidelines, read the publication in “Surg Endosc (2014) 28: page 358-360.”

**New statements**

**Table:** 

Level 1A	The rate of mesh infection after laparoscopic ventral and incisional hernia repair is low (1-2%) (stronger evidence)
Level 1A	If bridging is required, the use of a biologic mesh for replacement results in a very high recurrence rate (new statement)
Level 4	When conservative treatment of a mesh infection after laparoscopic ventral and incisional hernia repair fails, either a synthetic or biologic mesh seems to work as a replacement when facial closure can be achieved (new statement)

**New recommendation**

**Table:** 

Grade D	If bridging is required, the replacement of an infected mesh can be performed with a synthetic mesh (new recommendation)

**Comments**

In a randomized controlled trial with a total of 124 patients comparing laparoscopic versus open incisional hernia repair, no mesh infection was found in the laparoscopic, but two in the open group (1). In a registry-based study with 1757 laparoscopic and 1119 open incisional hernia repair with a median follow-up of 59 and 61 months, respectively, the mesh removal rates were 1% for the laparoscopic and 2.6% for the open group (2). The reasons for mesh removal were infection (63%), pain (19.6%), bowel obstruction (8.7%), and adhesions (6.5%) (2). In a retrospective review of a prospectively maintained database with 733 patients having undergone laparoscopic ventral hernia repair at a mean follow-up of 2.2 years, the need of a subsequent abdominal operation was documented (3). 15 patients (2.1%) needed infected mesh removal (3). The incidence of secondary mesh infection after subsequent operation was 2.4% (3). In a review article about replacement of infected meshes, the authors concluded that after failure of conservative treatment either a synthetic or a biological mesh seems to work as a replacement when fascial closure can be achieved (4). If bridging is required in laparoscopic ventral and incisional hernia repair, the use of a biologic mesh for replacement resulted in a very high recurrence rate (4). In a systematic review and single-institution experience, a hernia recurrence rate of 21.4% was achieved when the biologic mesh was placed in a retrorectus or underlay fashion after the removal of an infected mesh. Bridged repairs were very prone to recurrence (88.9%; *p* = 0.001) (5). In a consensus conference of the BioMesh Study Group, it was summarized that biologic mesh use should be avoided when bridging is needed in ventral hernia repairs due to the very high risk of recurrence (6).

**References** (in parentheses the level of evidence)

1. Rogmark P, Petersson U, Brigman S, Ezra E, Österberg J, Montgomery A (2016) Quality of Life and Surgical Outcome 1 Year After Open and Laparoscopic Incisional Hernia Repair. Ann Surg 263:244-250. 10.1097/sla.0000000000001305 (1B)
2. Kokotovic D, Bisgaard T, Helgstrand F (2016) Long-term Recurrence and Complications Associated With Elective Incisional Hernia Repair. JAMA 316(5):1575-1582. 10.1001/jama.216.15217 (2C)
3. Patel PP, Love MW, Ewing JA, Warren JA, Cobb WS, Carbonell AM (2017) Risks of subsequent abdominal operations after laparoscopic ventral hernia repair. Surg Endosc 31(2):823-828. 10.1007/s00464-016-5038-z (3)
4. Montgomery A, Kallinowski F, Köckerling F (2016) Evidence for Replacement of an Infected Synthetic by a Biological Mesh in Abdominal Wall Hernia Repair. Front Surg 2:67. 10.3389/fsurg.2015.00067. eCollection 2015. Review. (4)
5. Shubinetes V, Carney MJ, Colen DL, Mirzabeigi MN, Weissler JM, Lanni MA, Braslow BM, Fischer JP, Kovach SJ (2018) Management of infected mesh after abdominal hernia repair. Ann Plast Surg 80(2):145-153. 10.1097/sap.0000000000001189 (1A)
6. Köckerling F, Alam NN, Antoniou SA, Daniels IR, Famiglietti F, Fortelny RH, Heiss MM, Kallinowski F, Kyle-Leinhase I, Mayer F, Miserez M, Montgomery A, Morales-Conde S, Muysoms F, Narang SK, Petter-Puchner A, Reinpold W, Scheuerlein H, Smietanski M, Stechemesser B, Strey C, Woeste G, Smart NJ. (2018) What is he evidence for the use of biologic or biosynthetic meshes in abdominal wall reconstruction? Hernia 22(2):249-269.10.1007/s10029-018- 1735-y (2)

## Chapter 21. Postoperative Seroma: Risk Factors, Prevention, and Best Treatment

### J. Bingener, B. Ramshaw

**Key questions:**

1. Is obesity a risk factor for seroma formation?
2. Does the size of the hernia sack influence the frequency of seroma formation?
3. Will closure of the defect prevent seroma formation?
4. Could electrocauterization of the hernia sack be beneficial in preventing seromas?

Pubmed was searched with the search terms laparoscopic ventral hernia AND seroma. The search from August 2011 until September 2018 resulted in 98 new citations for the time period, with 12 studies relevant to the question, 1 multicenter RCT, 1 systematic review of low-level evidence study, 1 meta-analysis of retrospective study, 8 retrospective studies, and 1 registry study.

For the study of the original guidelines read, the publication in “Surg Endosc (2014) 28: page 360-361.”

**Table:** 

No changes in statements and recommendations with regard to incidence of seromas	

Risk factors

The old statements and recommendations of the original guidelines (see Surg Endosc (2014) 28:360-361) are still valid.

**New statement**

**Table:** 

Level 3	Robotic retrorectus approach may increase clinically detected seroma rate

Prevention

**New statement**

**Table:** 

Level 3	Closure of hernia defect may decrease seroma formation
Level 4	Abdominal binder may decrease seroma formation
Level 4	Injection of fibrin glue in the hernia sac may reduce seroma formation

**New recommendation**

**Table:** 

Grade C (meta-analysis of poor studies)	Surgeons should attempt to close the hernia defect when possible
Grade C (case report and small retrospective study)	Surgeons can consider injecting fibrin glue to prevent seroma

Since the publication of the guidelines, much effort has been made to investigate the effect of hernia defect closure in small-to-medium size hernias, including on seroma formation. A systematic review in 2014 [1] and a meta-analysis of 6 retrospective studies in 2016 revealed a decrease in seroma formation from 12–27 to 3–11% with defect closure compared to without defect closure [2].

Additional large retrospective studies and a prospective trial with hybrid surgical approach have confirmed these findings since then [3, 4].

Additional work has been done regarding abdominal binders and electrocautery of the hernia sac without changing the recommendations [5–7].

**References** (in parentheses graduation of evidence)

1. Nguyen DH, Nguyen MT, Askenasy EP, Kao LS, Liang MK (2014) Primary fascial closure with laparoscopic ventral hernia repair. systematic review. World J Surg 38(12):3097-104. (1A)
2. Tandon A, Pathak S, Lyons NJ, Nunes QM, Daniels IR, Smart NJ (2016) Meta-analysis of closure of the fascial defect during laparoscopic incisional and ventral hernia repair. Br J Surg 103(12):1598-1607. (2A)
3. Martin-Del-Campo LA, Miller HJ, Elliott HL, Novitsky YW (2018) Laparoscopic ventral hernia repair with and without defect closure: comparative analysis of a single-institution experience with 783 patients. Hernia 22(6):1061-1065. 10.1007/s10029-018-1812-2. Epub 2018 Aug 25 (3).
4. Ahonen-Siirtola M, Nevala T, Vironen J, Kössi J, Pinta T, Niemeläinen S, Keränen U, Ward J, Vento P, Karvonen J, Ohtonen P, Mäkelä J, Rautio T (2018) Laparoscopic versus hybrid approach for treatment of incisional ventral hernia: a prospective randomized multicenter study of 1-month follow-up results. Hernia 22(6):1015-1022. 10.1007/s10029-018-1784-2. Epub 2018 Jun 7. (1B).
5. Rothman JP, Gunnarsson U, Bisgaard T (2014) Abdominal binders may reduce pain and improve physical function after major abdominal surgery—a systematic review. Dan Med J 61(11)A4941. (1A)
6. Prassas D, Schumacher FJ (2018) Electric cauterization of the hernia sac in laparoscopic ventral hernia repair reduces the incidence of postoperative seroma: a propensity score-matched analysis. Hernia 22(5):747-750. 10.1007/s10029-018-1790-4. Epub 2018 Jun 14 (3)
7. Lambrecht JR1, Vaktskjold A, Trondsen E, Øyen OM, Reiertsen O (2015) Laparoscopic ventral hernia repair: outcomes in primary versus incisional hernias: no effect of defect closure. Hernia 19(3):479-86. (3)
8. Clapp ML, Hicks SC, Awad SS, Liang MK (2013) Trans-cutaneous closure of central defects (TCCD) in laparoscopic ventral hernia repairs (LVHR). World J Surg 37(1):42-51. (3)
9. Huang CC, Lien HH, Huang CS (2013) Long-term follow-up of laparoscopic incisional and ventral hernia repairs. J Laparoendosc Adv Surg Tech A 23(3):199-203. (3)
10. Morales-Conde S, Suarez-Artacho G, Socas M, Barranco A (2013) Influence of fibrin sealant in preventing postoperative seroma and normalizing the abdominal wall after laparoscopic repair of ventral hernia. Surg Endosc 27:3214-3219. (3)
11. Karim MA, Ali A (2013) Simple technique to manage redundant skin after laparoscopic ventral hernia repair. Asian J Endosc Surg 6(2):137-9. (4)
12. Wennergren JE, Askenasy EP, Greenberg JA, Holihan J, Keith J, Liang MK, Martindale RG, Trott S, Plymale M, Roth JS (2016) Laparoscopic ventral hernia repair with primary fascial closure versus bridged repair: a risk-adjusted comparative study. Surg Endosc 30(8):3231-8. (3)

## Chapter 22. Postoperative bulging

### Jianxiong Tang, L. Jorgensen, ShaoJie Li, Lei Zhu

**Key questions:**

1. Is bulging a result of insufficient overlap of the mesh?
2. Is bulging a result of insufficient fixation?
3. Is the occurrence of bulging related to the size of the hernia defect?
4. How can bulging be prevented?
5. Should bulging be treated?

**Search terms:**

“laparoscopic incisional hernia repair” AND “incisional hernia,” “ventral hernia,” “postoperative bulging abdominal wall” AND “abdominal wall bulging” AND “abdominal wall hernia and bulging” AND “complication bulging” AND “incisional hernia and bulging” AND “bulging after hernia repair” AND “protrusion” AND “eventration” AND “pseudo recurrence”

A systematic search of the available literature was performed in October 2017 based on Medline, PubMed, Web of Science, and the Cochrane Library, as well as relevant journals and reference lists using the aforementioned search terms. The first search found 110 relevant articles. In a second-level search, 13 articles were added. A total of 19 publications were used for this systematic review.

Statements and recommendations of the original guidelines (see Surg Endosc (2014) 28: page 361-362) are still valid and therefore they are not mentioned again.

**New statements**

**Table:** 

Level 3	Failure to position/fix the mesh flat may contribute to postoperative bulging
Level 3	Larger hernia defect size is associated with a higher rate of postoperative bulging
Level 4	There is no significant difference in the incidence of bulging according to mesh fixation technique
Level 4	Mesh type may influence the rate of postoperative bulging

**New recommendations**

**Table:** 

Grade B	It is recommended that mesh should be tensioned appropriately such that the mesh is flat without any wrinkles/folds following desufflation of the abdomen
Grade C	Larger defects need a larger overlap to resist the intra-abdominal forces.

**Introduction**

As one of the adverse outcomes of laparoscopic ventral hernia repair (LVHR), unlike recurrence and pain, postoperative bulging which can be cosmetically dissatisfying was rarely mentioned in previous literature. A bulge in the area of previous repair of a ventral or incisional hernia which is frequently impossible to clinically differentiate from a recurrence is usually called “postoperative bulging” [1]. However, the name of this condition is still inconsistent. Lambrecht et al. [2] utilized “mesh protrusion,” some surgeons called it “eventration” [3–7], and Cater [4] and Tse [8] referred to this condition below “pseudorecurrence” in their manuscripts, respectively.

The anatomic basis for this problem lies in the fact that neither the hernia defect nor the rectus diastasis (if present) was closed during laparoscopic hernia repair. These issues, relevant mainly with large hernias, should be discussed with the patient preoperatively [9].

This section concerns the prevalence, diagnosis, clinical significance, and treatment of bulging in the area of laparoscopic repair of ventral hernia caused by mesh protrusion through the hernia opening, but with intact peripheral fixation of the mesh forming an adequate repair.

In a study of 765 patients who underwent laparoscopic ventral hernia repair, all the patients with swelling in the repaired area (*n* = 29) were identified and subjected to further examination by computed tomography (CT). The exam showed that 17 patients (2.2%) had a recurrence hernia. For additional 12 patients (1.6%), the CT indicated only bulging of the mesh but no recurrence [1]. Liang et al. found 17 of 79 (21.5%) patients developed postoperative bulging in their LVHR group when it was 1.3% in open ventral hernia repair group in their retrospective case–control study [10].

- Is bulging a result of insufficient overlap of the mesh?

In the comparative study of Lambrecht et al. [2], large overlap (overlap ≥ 5 cm) counters the risk of mesh protrusion (OR 0.59; CI 0.16–2.13). However, as Cater et al. [4] reported, mesh overlap in their patients with no eventration (*n* = 49), mesh eventration (*n* = 38), tissue eventration (*n* = 25) was 3.6 ± 0.2 cm, 3.3 ± 0.2 cm, 3.3 ± 0.2 cm respectively, and there was no significant statistically difference. This is partly correlated with the size of the abdominal defect. According to experience of Cobb [11], lager defect may need more overlap of mesh to resist the intra-abdominal forces that act to push the mesh into the hernia defect.

As reported previously, even with a sufficient repair, remaining patients experienced a postoperative bulging [1]. In their study, the four patients who had symptoms suggestive of recurrent hernia but in whom laparotomy or laparoscopy definitively excluded recurrence were all found to have bulging of the mesh. Meanwhile, in three of them, laparoscopy showed protrusion or bulging of mesh that may have been too loosely stretched across the hernia defect during LIVHR [1].

So, it could be more appropriate to mention the effective sufficient overlap, other than only insist on the value of the overlap. Mesh should be stretched tightly over the defect and whole scar while sufficient overlap is performed in the LVHR.

• **Is bulging a result of insufficient fixation?**

Also in Schoenmaeckers’s study [1], the mesh was fixed either by a double circle of tacks a technique popularly known as a “double crown” (*n* = 455), or with a single circle of tacks along the periphery of the mesh and transabdominal sutures placed equidistant along the perimeter of the mesh (*n* = 310). There was no significant difference in the incidence of bulging according to mesh fixation technique (double crown of tacks 5/455 versus transabdominal sutures and tacks 7/310, NS).

• **Is the occurrence of bulging related to the size of the hernia defect?**

Cater [4] found that while hernia area (cm^2^) of 49 patients without eventration is 22.3 ± 4.9, this value got approximately doubled (48.8 ± 8.7, *p* = 0.006) in their 38 patients with mesh eventration. Similar result was verified in another study [2], for patients with large hernia size (ellipsoid hernia area > 20 cm^2^) the OR for protrusion was 2.30 (CI 0.73–7.19). In a biomechanical study of Guérin et al. [12], the defect size was also found that it is the most influent parameter with a significant increase of bulging. Interestingly, bulging was observed in a wide range of initial hernia defects, and no relation was observed between hernia defect size and development of bulging in Schoenmaeckers’s study, an absolutely contrary result [1].

**• How to prevent bulging?**

To reduce the incidence bulging, Orenstein et al. [13] modified their LVHR approach to routine closure of the fascia defect (“shoelacing” technique) before mesh placement. In their study, 47 consecutive patients undergoing LVHR with shoelacing were reviewed retrospectively. While providing reliable hernia repair, the addition of defect closure and routine use of the shoelace technique reduced bulging in their experience.

Mitura et al. [14] compared standard laparoscopic intraperitoneal onlay mesh repair (IPOM) technique and closure of hernia defect in IPOM-plus technique of small and medium midline ventral hernia repair in their prospective, single-center study. After 12 months, 2 cases of mesh eventration were confirmed in IPOM group (2/40) and none in IPOM-plus group (0/42).

In a retrospective case–control study (*n* = 36, 1:1), Clapp et al. [3] reported that laparoscopic ventral hernia repair with trans-cutaneous closure of central defects (LVHR-TCCD) procedure had significantly lower rates of mesh eventration (0.0% versus 41.4%, *p* = 0.0002), tissue eventration (4.0% versus 37.9%, *p* = 0.003), clinical eventration (8.3% versus 69.4%, *p* = 0.0001) when compared to the LVHR.

Agarwal et al. [15] introduced a “Double-Breasted” fascial closure of hernia defect technique in their LVHR with IPOM in 2008 and found no postoperative bulging in their 30 patients with a mean follow-up of 58 months (range 26–84).

Another study confirmed the positive results of defect closure, in a single-institutions experience in 1326 laparoscopic incisional and ventral hernia repairs. Laparoscopy approximating the linea alba under physiological tension was assigned by either the transparietal U reverse-interrupted stitches or the extracorporeal closure in larger defects; there were only 1.5% (20/1326) patients who developed skin bulging while seroma counted together [16].

However, there is also voice from opposite side; Lambrecht et al. [2] declared that defect closure had no significant effect on mesh protrusion in their two separate studies comprising 225 patients.

In a retrospective review of 201 consecutive patients who underwent elective LVHR at two institutions, it was found that besides defect closure the mesh type has also a statistically significant influence on the development of postoperative eventration or not. Patients who underwent LVHR with polypropylene mesh (43/57), or polytetrafluoroethylene mesh (7/9), were found to have significantly higher rates of mesh and tissue eventration than patients repaired with polyethylene mesh (12/45) [4].

Some surgeons described a condition that retained pre-peritoneal fat or hernia sac extends beyond the boundaries of the anterior abdominal wall fascia, and defined this as “tissue eventration” [3, 4, 6]. To a large extent, failure to remove the hernia sac intraoperatively lead to “tissue eventration.” They also believed that closure of, especially larger, defects results in no longer a “tension-free” repair. However, the primary goal of repair is to achieve apposition of the fascial edges with reinforcement of the muscle layers. The lack of any fascial or muscular covering of the defect has the consequence that only the mesh buttresses the defect against intra-abdominal pressure. That may predispose to protrusion of the mesh into the hernia. In fact, excise redundant sac and repair of the fascial defect are general principles which dictate the management of abdominal wall hernias [17].

• **Should bulging be treated?**

Schoenmaeckers et al. [1] reported that bulging was associated with pain in four patients, who underwent re-laparoscopy and got a new, larger mesh tightly stretched over the entire previous repair in their study. Other eight asymptomatic patients agreed to “watchful waiting.” All the patients remained symptom-free during a median follow-up period of 22 months.

Symptomatic bulging, although not a recurrence, requires a new repair and must be considered as an important negative outcome of laparoscopic ventral hernia repair. For asymptomatic patients without loss of abdominal domain, “watchful waiting” seems justified.

**Comment**

Symptomatic bulging, although not a recurrence, requires a new repair and must be considered as an important negative outcome of laparoscopic ventral hernia repair. For asymptomatic patients without loss of abdominal domain, “watchful waiting” seems justified. The addition of defect closure in LVHR could reduce the rate of postoperative bulging. Larger defects may need larger overlap of mesh to resist the intra-abdominal forces that act to push the mesh into the hernia defect. Last but very important, mesh should be stretched tightly over the defect and whole scar while sufficient overlap is performed in the LVHR.

Abdominal bulging is a specific problem associated with laparoscopic repair of large incisional hernias. It occurs in 1.6–21.5% of patients [1, 8, 10, 14, 18, 19]. This wide range may be attributable in part to surgical technique differences between surgeons whether close hernia defect, or in part to interpretation by the examiner and the opinion of the patient, but evidence for this is limited. There is an urgent need for more studies regarding this topic.

**References** (in parentheses graduation of evidence)

1. Schoenmaeckers E J P, Wassenaar E B, Raymakers J T F J, Rakic S (2010) Bulging of the mesh after laparoscopic repair of ventral and incisional hernias. Journal of the Society of Laparoendoscopic Surgeons 14:541-546. 10.4293/108680810x12924466008240 (2C)
2. Lambrecht J R, Vaktskjold A, Trondsen E, Oyan OM, Reiestsen O (2015) Laparoscopic ventral hernia repair: outcomes in primary versus incisional hernias: no effect of defect closure. Hernia 19: 479-486. 10.1007/s10029-015-1345-x(3)
3. Clapp M L, Hicks S C, Awad S S, Liang MK (2013) Trans-cutaneous Closure of Central Defects (TCCD) in laparoscopic ventral hernia repairs (LVHR). World J Surg 37: 42-51. 10.1007/s00268-012-1810-y (3)
4. Carter S A, Hicks S C, Brahmbhatt R, Liang MK (2014) Recurrence and pseudorecurrence after laparoscopic ventral hernia repair: predictors and patient-focused outcomes. Am Surg 80: 138-148. (4)
5. Liang M K, Clapp M, Li L T, Burger RL, Hicks SC, Awad S (2013) Patient satisfaction, chronic pain, and functional status following laparoscopic ventral hernia repair. World J Surg 37: 530-537. 10.1007/s00268-012-1873-9 (3)
6. Huang C C, Lien H H, Huang C S (2013) Long-term follow-up of laparoscopic incisional and ventral hernia repairs. J Laparoendosc Adv Surg Tech A 23: 199-203. 10.1089/lap.2012.0359. (2B)
7. Liang M K, Subramanian A, Awad S S (2012) Laparoscopic transcutaneous closure of central defects in laparoscopic incisional hernia repair. Surg Laparosc Endosc Percutan Tech 22: e66-e70. 10.1097/sle.0b013e3182471fd2 (4)
8. Tse G H, Stutchfield B M, Duckworth A D, de Beaux AC, Tulloh B (2010) Pseudo-recurrence following laparoscopic ventral and incisional hernia repair. Hernia 14: 583-587. 10.1007/s10029-010-0709-5 (4)
9. Sauerland S, Walgenbach M, Habermalz B, Seiler CM, Miserez M (2011) Laparoscopic versus open surgical techniques for ventral or incisional hernia repair. Cochrane Database Syst Rev 16: CD007781. 10.1002/14651858.cd007781.pub2 (1A)
10. Liang, Mike K., Li LT, Davila JA, Hicks SC, Kao LS (2013) Outcomes of laparoscopic vs open repair of primary ventral hernias. JAMA surgery 148: 1043-1048. 10.1001/jamasurg.2013.3587 (3)
11. Cobb, WS, Kercher KW, Matthews BD, Burns JM, Tinkham NH, Sing RF, Heniford BT (2006) Laparoscopic ventral hernia repair: a single center experience. Hernia 10: 236-242. 10.1007/s10029-006-0072-8(4)
12. Guérin, G., and F. Turquier (2013) Impact of the defect size, the mesh overlap and the fixation depth on ventral hernia repairs: a combined experimental and numerical approach. Hernia 17: 647-655.10.1007/s10029-013-1050-6 (5)
13. Orenstein SB, Dumeer JL, Montegudo J, Poi MJ, Novitsky YW (2011) Outcome of laparoscopic ventral hernia repair with routine defect closure using shoelacing technique. Surg Endosc 25:1452-1457. 10.1007/s00464-010-1413-3 (4)
14. Mitura K, Skolimowska-Rzewuska M, Garnysz K (2017) Outcomes of bridging versus mesh augmentation in laparoscopic repair of small and medium midline ventral hernias. Surg Endosc 31: 382-388. 10.1007/s00464-016-4984-9 (3)
15. Agarwal, Brij B, Gupta MK, Mishra A, Mahajan KC (2008) Laparoscopic ventral hernia meshplasty with double-breasted fascial closure of hernial defect: a new technique. J Laparoendosc Adv Surg Tech A 18: 222-229. 10.1089/lap.2007.0112 (4)
16. Chelala E, Baraké H, Estievenart J, Dessily M, Charara F, Allé JL (2016) Long-term outcomes of 1326 laparoscopic incisional and ventral hernia repair with the routine suturing concept: a single institution experience. Hernia 20: 101-110. 10.1007/s10029-015-1397-y (4)
17. Bennett, David H (2013) Principles in hernia surgery. Management of Abdominal Hernias. Springer London 91-101. (5)
18. Kurmann A, Visth E, Candinas D, Beldi G (2011) Long-term follow-up of open and laparoscopic repair of large incisional hernias. World journal of surgery 35: 297-301. 10.1007/s00268-010-0874-9 (2B)
19. Ghali S, Turza KC, Baumann DP, Butler CE (2010) Minimally invasive component separation results in fewer wound-healing complications than open component separation for large ventral hernia repairs. Journal of the American College of Surgeons 214: 981-989. 10.1016/j.jamcollsurg.2012.02.017. (3)

## Chapter 23. Chronic Pain—Risk Factors, Prevention, and Treatment

### J. Bingener, W. Reinpold

**Key questions:**

1. Is chronic pain related to the type of fixation?
2. Is chronic pain related to the number of fixation tacks?
3. Is chronic pain related to the size of the defect?
4. Is chronic pain related to the type or the size of the mesh?
5. Which is the best treatment option in patients suffering from chronic pain?

Statements and recommendations of the original guidelines (see Surg Endosc (2014) 28: page 362-364) are still valid, and therefore they are not mentioned again, except the statements/recommendations mentioned as follows:

The search terms ‘laparoscopic ventral hernia’ AND ‘pain’ were used in Pubmed and resulted in 198 citations from August 2011 to September 2018. Of those, 6 new studies addressed the topic: 1 meta-analysis, 1 RCT, and 4 retrospective studies. Overall, the studies were of moderate to poor quality. The meta-analysis served to increase the certainty of prior statements and recommendations (improved level of evidence from 4 to 2A on prevalence of chronic pain and role of absorbable tacks in chronic pain prevention) [1]. The frequency of chronic pain was also confirmed by three retrospective studies [2−4]. Additional findings regarding the prevention of chronic pain were the usefulness of intraoperative application of local anesthetic as regional block, such as transversus abdominis plane block compared to placebo, as documented in the RCT by Fields et al. [5] and a clinical quality improvement project by Ramshaw et al. [6].

**Risk factors**

*Statement*

**Table:** 

Level 2A	LVHR results in chronic pain in up to 25% of patients

**Non-procedure-specific risk factor**

**Table:** 

*Statement*	No change

**Prevention**

*Statements*

**Table:** 

Level 2B	Local anesthetic at suture sites and as transversus abdominis plane block during surgery significantly decreases acute early pain
Level 2A	Absorbable fixation tacks were not associated with less chronic pain

*Recommendations*

**Table:** 

Grade B	Surgeons should use intraoperative suture site or regional block injection of local anesthetic

**Treatment**

**Table:** 

*Statements and Recommendations*	No change

**References** (in parentheses the level of evidence)

1. Khan RMA, Bughio M, Ali B, Hajibandeh S, Hajibandeh (2018) Absorbable versus non-absorbable tacks for mesh fixation in laparoscopic ventral hernia repair: A systematic review and meta-analysis. Int J Surg 53:184-192. (2A)
2. Langbach O, Bukholm I, Benth JS, Rokke O (2015) Long term recurrence, pain and patient satisfaction after ventral hernia mesh repair. World J Gastrointest Surg 7(12):384-93. (4)
3. Liot E, Breguet R, Piguet V, Ris F, Volonte F, Morel P (2017) Evaluation of port site hernias, chronic pain and recurrence rates after laparoscopic ventral hernia repair: A monocentric long-term study. Hernia 21(6):917-923. (4)
4. Liang MK, Clapp M, Li LT, Berger RL, Hicks SC, Awad S (2013) Patient satisfaction, chronic pain, and functional status following laparoscopic ventral hernia repair. World J Surg 37(3)530-7. (4)
5. Fields AC, Gonzalez DO, Chin EH, Nguyen SQ, Zhang LP, Divino CM (2015) Laparoscopic-assisted transversus abdominis plane block for postoperative pain control in laparoscopic ventral hernia repair: A randomized controlled trial. J Am Coll Surg 221(2):462-469. (2B)
6. Ramshaw B, Forman B, Heidel E, Dean J, Gamenthaler A, Fabian M (2016) A clinical quality improvement (CQI) project to improve pain after laparoscopic ventral hernia repair. Surg Technol Int 29:125-130. (3B)

## Chapter 24. Recurrence after laparoscopic ventral/incisional hernia repair—risk factors, mechanism, and prevention

### P. Chowbey, D. Chen, R. Khullar

**Key questions:**

1. Is the occurrence of a recurrence dependent on the size of the defect?
2. Is the occurrence of a recurrence dependent on the size or the type of the mesh?
3. Is the occurrence of a recurrence dependent on the type of fixation?
4. Is the occurrence of a recurrence dependent on the extent of the overlapping?
5. Is the occurrence of a recurrence dependent on risk factors like smoking, immunosuppressive drugs, chronic pulmonary diseases, liver cirrhosis, obesity?

**Search terms**

The following search terms were used: incisional hernia and “recurrence,” “recurrence” and risk factors” and “incisional hernia,” “incisional hernia” and “prevention of recurrence,” “incisional hernia” and mechanism of recurrence.”

Search machines

PubMed, Medline, the Cochrane Library, EMBASE, the British Journal of Surgery database, Google scholar, Scirus, Ovid

**New Publications**

A total of 5 new studies were identified since the publication of original guidelines. Statements and guidelines were modified accordingly. For the study of the original guidelines, read the publication in “Surg Endosc (2014) 28: page 364-365.”

**RISK FACTORS FOR RECURRENCE**

**New statements**

**Table:** 

Level 2c	Pregnancy after ventral hernia repair is independently associated with ventral hernia recurrence
Level 4	Previous interventions, postoperative complications, and Clavien–Dindo score > 2 are independent prognostic factors for recurrence

**New recommendation**

**Table:** 

Grade B	Female patients of reproductive age who wish to have ventral hernia repair should be advised on the increased risk of recurrence associated with subsequent pregnancy

**PREVENTION OF RECURRENCE**

**New statements**

**Table:** 

Level 1	Mesh reinforcement is recommended for ventral hernia repairs in a clean case
Level 3	Sublay mesh location may result in fewer recurrences
Level 1	Risk of hernia recurrence decreases with increasing area of mesh overlap in laparoscopic procedures for ventral hernia repair
Level 4	Mesh-to-defect area ratio is an independent predictive factor for recurrence

**New recommendation**

**Table:** 

Grade B	Larger hernia defect sizes require greater mesh overlap to ensure that optimal mesh-to-defect area ratio is achieved

**COMMENTS**

In a nationwide cohort study including 3578 female patients of reproductive age registered in the Danish Ventral Hernia Database with ventral hernia repair between 2007 and 2013, 267 (7.5%) of them subsequently became pregnant during follow-up [1]. Median follow-up was 3.1 years (range 0–8.4 years). Pregnancy was independently associated with recurrence (hazard ratio 1.56, 95% confidence interval 1.09–2.25, *p* = 0.016).

In a prospective review of 417 patients from 2005 to 2014 who underwent laparoscopic ventral hernia repair, previous interventions (OR 1.44; CI 1.15–1.79; *p* = 0.01), postoperative complications (OR 2.57; CI 1.09–6.03; *p* = 0.03), and Clavien–Dindo score > 2 (OR 1.43; CI 1.031–1.876; *p* = 0.02) appeared as independent prognostic factors of recurrence [2].

In a meta-analysis of randomized controlled trials including 23 studies, evidence indicates that mesh reinforcement in clean cases can decrease hernia recurrence. Also placing mesh in sublay position (as opposed to the onlay and underlay position) may decrease the risk of hernia recurrence [3].

A meta-analysis including a total of 95 articles, with 111 study populations [4], showed that for open procedures results showed no correlation between the pooled estimation of risk for recurrence of ventral hernia and area of mesh overlap used for hernia repair (< 3 cm, incidence rate 0.065; 3–5 cm, incidence rate 0.070; > 5 cm, incidence rate 0.060). However, in laparoscopic procedures, the pooled estimation of risk for recurrence of hernia decreased with increasing area of mesh overlap (< 3 cm, incidence rate 0.086; 3–5 cm, incidence rate 0.046; > 5 cm, incidence rate 0.014).

In a study consisting of 213 consecutive patients operated by laparoscopy for primary ventral (*n* = 158) or incisional hernia (*n* = 55) between 2001 and 2014, it was revealed that mesh-to-defect area ratio was the only independent predictive factor for recurrence [5, see chapter 14]. With mesh-to-defect area ratio </=8, between 9 and 12, between 13 and 16, and >/=17, recurrence rate was, respectively, 70, 35, 9, and 0% (*p* < 0.001).

**References** (in parentheses the level of evidence)

1. Oma E, Jensen KK, Jorgensen LN (2017) Increased risk of ventral hernia recurrence after pregnancy: A nationwide register-based study. The American Journal of Surgery 214(3):474–8. [2C]
2. Mercoli H, Tzedakis S, D’Urso A, Nedelcu M, Memeo R, Meyer N, et al. (2016) Postoperative complications as an independent risk factor for recurrence after laparoscopic ventral hernia repair: a prospective study of 417 patients with long-term follow-up. Surgical Endosc 31(3):1469–77. [4]
3. Holihan JL, Hannon C, Goodenough C, Flores-Gonzalez JR, Itani KM, Olavarria O, et al. (2017) Ventral Hernia Repair: A Meta-Analysis of Randomized Controlled Trials. Surg Infect 18(6):647–58. [1A]
4. LeBlanc K (2016) Proper mesh overlap is a key determinant in hernia recurrence following laparoscopic ventral and incisional hernia repair. Hernia 20(1):85–99. 10.1007/s10029-015-1399-9. Epub 2015 Jul 5 [1A]
5. Hauters P, Desmet J, Gherardi D, Dewaele S, Poilvache H, Malvaux P (2017) Assessment of predictive factors for recurrence in laparoscopic ventral hernia repair using a bridging technique. Surg Endosc 31 (9):3656–3663. 10.1007/s00464-016-5401-0 (3)

## Chapter 25. Comparison of open vs. laparoscopic hernia repair: Operation room time, bowel lesion, seroma, and wound infection

### Jianwen Li, Fei Yue, Zirui He

**Key question:**

1. Are there differences between open and laparoscopic repairs by operating room time, bowel injury, seroma, and wound infection?

**Search terms**

The following search terms were used: “open”; “laparoscopic”; “incisional”; “ventral”; “hernia.”

**Search machines**

PubMed, Medline, and the Cochran Library as well as the reference lists of the included studies were searched for relevant studies.

**New publications**

A total of 42 new studies were identified since the publication of the original guidelines. Statements and recommendations were modified accordingly. For the study of the original guidelines, read the publication in “Surg Endosc (2014) 28: page 366–369.”

**New statements**

**Table:** 

Level 1B	Learning curve in terms of time of operation for LVHR is around 50 Cases (new statement)
Level 2A	The laparoscopic approach has a significantly lower risk for wound infections in incarcerated/strangulated hernias (new statement)
Level 3	Missed bowel injuries are more common in laparoscopic surgery, and may lead to major complications (new statement)

**New recommendations**

**Table:** 

Identical to previous	

**Comments**

We identified 25 new publications concerning operating room (OR) time [1–25]. Four level 1a studies showed inconclusive results [1–4]. Al Chalabi’s study reached a borderline statistical difference in OR time with *p* = 0.05[1]. Zhang’s meta-analysis could not perform pooled analyses due to the heterogeneity [3]. This included 4 RCTs that favored open repair and 2 RCTs that favored laparoscopic repair. Moreau stated that laparoscopy does not increase OR time compared with open approach [4]. Awaiz found that OR time was comparable between different groups [2]. Two level 1b studies demonstrated that laparoscopic repair resulted in longer OR time, especially in the first 50 operations [7], while the other one indicated no difference [6]. The results of level 2 and 3 studies are inconclusive. Hence, a new statement that the first 50 laparoscopic repairs result in longer OR time was added, which reflects the learning curve.

Among 14 new articles which were relevant to bowel injury [2–5, 7, 13, 15, 17, 19, 21, 25–28], the majority of level 1 studies indicated less bowel injuries in the open group. Only one level 2a study favored laparoscopy [26]. The remaining articles reached no significant difference. The new statement added is substantially in accordance with previous level 1a statement. Bowel injuries usually happened during the process of adhesiolysis. If the injury was detected and sutured intraoperatively, there will be no complications. Missed enterotomies result in severe complications, including reoperations and death [25].

Most of the new publications (20/24) related to the incidence of seroma showed no significant difference between the two approaches [2, 3, 5, 9, 12–14, 17–20, 23, 25, 27, 29–34]. Notably, there were three studies that favored the laparoscopic approach, which were supported with level 1b [7], level 2a [26], and level 3 [24] evidence. Meanwhile, only one level 3 study favored the open procedure [28]. There is a correlation between laparoscopic approach and lower seroma rate, but it is not a clear causal link. Further studies might provide additional solid lines of evidence in the future.

Of the 35 new published articles involved with wound infection, 24 studies reported less wound infection with laparoscopy [1, 3, 6–9, 13, 19, 21, 24, 26, 27, 29–40], and 11 more reported no significant difference between two groups [2, 4, 5, 12, 14, 20, 23, 25, 28, 41, 42]. No study supported open approach. The emerging data with high-level evidence confirmed lower incidence of wound infection with the laparoscopic procedure, compared with open repair. Kaoutzanis and his colleagues performed a study based on the data from ACS-NSQIP [8]. A total of 5943 incarcerated/strangulated ventral or incisional hernias were included, among which 1420 were repaired with laparoscopic procedures. Both superficial and deep surgical site infection rates were significantly lower in the laparoscopic group, and wound disruption as well. Thus, we add the new statement especially for the laparoscopic management of incarcerated/strangulated cases. Nowadays, laparoscopic repair is also an option for the hernia specialists when dealing with incarcerated/strangulated ventral or incisional hernias.

**References** (in parentheses the level of evidence)

1. Al Chalabi H, Larkin J, Mehigan B, McCormick P (2015) A systematic review of laparoscopic versus open abdominal incisional hernia repair, with meta-analysis of randomized controlled trials. Int J Surg 20:65–74. 10.1016/j.ijsu.2015.05.050 (1A)
2. Awaiz A, Rahman F, Hossain MB, Yunus RM, Khan S, Memon B, Memon MA (2015) Meta-analysis and systematic review of laparoscopic versus open mesh repair for elective incisional hernia. Hernia 19(3):449–463. 10.1007/s10029-015-1351-z (1A)
3. Zhang Y, Zhou H, Chai Y, Cao C, Jin K, Hu Z (2014) Laparoscopic versus open incisional and ventral hernia repair: a systematic review and meta-analysis. World J Surg 38(9):2233–2240. 10.1007/s00268-014-2578-z (1A)
4. Moreau PE, Helmy N, Vons C (2012) Laparoscopic treatment of incisional hernia. State of the art in 2012. J Visc Surg 149(5 Suppl):e40–48. 10.1016/j.jviscsurg.2012.09.001 (1A)
5. Eker HH, Hansson BM, Buunen M, Janssen IM, Pierik RE, Hop WC, Bonjer HJ, Jeekel J, Lange JF (2013) Laparoscopic vs. open incisional hernia repair: a randomized clinical trial. JAMA Surg 148(3):259–263. 10.1001/jamasurg.2013.1466 (1B)
6. Rogmark P, Petersson U, Bringman S, Eklund A, Ezra E, Sevonius D, Smedberg S, Osterberg J, Montgomery A (2013) Short-term outcomes for open and laparoscopic midline incisional hernia repair: a randomized multicenter controlled trial: the ProLOVE (prospective randomized trial on open versus laparoscopic operation of ventral eventrations) trial. Ann Surg 258(1):37–45. 10.1097/sla.0b013e31828fe1b2 (1B)
7. Malik AM (2015) Laparoscopic versus open repair of para-umbilical hernia. Is it a good alternative? J Pak Med Assoc 65(8):865–868. (1B)
8. Kaoutzanis C, Leichtle SW, Mouawad NJ, Welch KB, Lampman RM, Cleary RK (2013) Postoperative surgical site infections after ventral/incisional hernia repair: a comparison of open and laparoscopic outcomes. Surg Endosc 27(6):2221–2230. 10.1007/s00464-012-2743-0 (2A)
9. Salvilla SA, Thusu S, Panesar SS (2012) Analyzing the benefits of laparoscopic hernia repair compared to open repair: A meta-analysis of observational studies. J Minim Access Surg 8(4):111–117. 10.4103/0972-9941.103107 (1A)
10. Coratti F, Coratti A, Malatesti R, Varrone F, Testi W, Savelli V, Borgogni V. (2014) Treatment of median incisional hernia. Laparoscopic vs. open surgery: meta-analysis. Ann Ital Chir 85(4):358–364. (1A)
11. Anadol AZ, Akin M, Kurukahvecioglu O, Tezel E, Ersoy E (2011) Comparison of laparoscopic primary and open primary repair of ventral hernias. Surg Laparosc Endosc Percutan Tech 21(5):301–305. 10.1097/sle.0b013e3182245d61 (2B)
12. Asti E, Sironi A, Lovece A, Bonitta G, Bonavina L (2016) Open Versus Laparoscopic Management of Incisional Abdominal Hernia: Cohort Study Comparing Quality of Life Outcomes. J Laparoendosc Adv Surg Tech A 26(4):249–255. 10.1089/lap.2016.0060 (2B)
13. Colavita PD, Tsirline VB, Belyansky I, Walters AL, Lincourt AE, Sing RF, Heniford BT (2012) Prospective, long-term comparison of quality of life in laparoscopic versus open ventral hernia repair. Ann Surg 256(5):714–722; discussion 722–713. 10.1097/sla.0b013e3182734130 (2B)
14. Moreno-Egea A, Alcaraz AC, Cuervo MC (2013) Surgical options in lumbar hernia: laparoscopic versus open repair. A long-term prospective study. Surg Innov 20(4):331–344. 10.1177/1553350612458726 (2B)
15. Soliani G, De Troia A, Portinari M, Targa S, Carcoforo P, Vasquez G, Fisichella PM, Feo CV (2017) Laparoscopic versus open incisional hernia repair: a retrospective cohort study with costs analysis on 269 patients. Hernia 21(4):609–618. 10.1007/s10029-017-1601-3 (3)
16. Khan JS, Qureshi U, Farooq U, Hassa ZF, Hassan H (2012) The comparison of open and laparoscopic ventral hernia repairs. J Postgrad Med Inst 26(4):397–401. (2B)
17. Moreno-Egea A, Sanchez-Elduayen M, De Andres EP, Carrillo-Alcaraz A. (2012) Is muscular atrophy a contraindication in laparoscopic abdominal wall defect repair? A prospective study. Am Surg 78(2):178–184. (2B)
18. Schroeder AD, Debus ES, Schroeder M, Reinpold WM. (2013) Laparoscopic transperitoneal sublay mesh repair: a new technique for the cure of ventral and incisional hernias. Surg Endosc 27(2):648–654. 10.1007/s00464-012-2508-9 (3)
19. Ahonen-Siirtola M, Rautio T, Ward J, Kössi J, Ohtonen P, Mäkelä J (2015) Complications in Laparoscopic Versus Open Incisional Ventral Hernia Repair. A Retrospective Comparative Study. World J Surg 39(12):2872–2877. 10.1007/s00268-015-3210-6 (3)
20. Azoury SC, Dhanasopon AP, Hui X, Tuffaha SH, De La Cruz C, Liao C, Lovins M, Nguyen HT (2014) Endoscopic component separation for laparoscopic and open ventral hernia repair: a single institutional comparison of outcomes and review of the technique. Hernia 18(5):637–645. 10.1007/s10029-014-1274-0 (3)
21. Froylich D, Segal M, Weinstein A, Hatib K, Shiloni E, Hazzan D (2016) Laparoscopic versus open ventral hernia repair in obese patients: a long-term follow-up. Surg Endosc 30(2):670–675. 10.1007/s00464-015-4258-y (3)
22. Stipa F, Giaccaglia V, Burza A, Santini E, Bascone B, Picchio M. (2013) Incisional hernia: laparoscopic or open repair? Surg Laparosc Endosc Percutan Tech 23(4):419–422. 10.1097/sle.0b013e31828e3c33 (3)
23. Tsuruta A, Hirai T, Nakamura M (2014) Retrospective comparison of open versus laparoscopic ventral and incisional hernia repair. Asian J Endosc Surg 7(3):246–250. 10.1111/ases.12108 (3)
24. Langbach O, Bukholm I, Benth JS, Røkke O (2015) Long term recurrence, pain and patient satisfaction after ventral hernia mesh repair. World J Gastrointest Surg 7(12):384–393. 10.4240/wjgs.v7.i12.384 (3)
25. Ahonen-Siirtola M, Rautio T, Biancari F, Ohtonen P, Mäkelä J (2017) Laparoscopic versus Hybrid Approach for Treatment of Incisional Ventral Hernia. Dig Surg 34(6):502–506. 10.1159/000458713 (3)
26. Ecker BL, Kuo LE, Simmons KD, Fischer JP, Morris JB, Kelz RR (2016) Laparoscopic versus open ventral hernia repair: longitudinal outcomes and cost analysis using statewide claims data. Surg Endosc 30(3):906–915. 10.1007/s00464-015-4310-y (2C)
27. Kokotovic D, Bisgaard T, Helgstrand F (2016) Long-term Recurrence and Complications Associated With Elective Incisional Hernia Repair. JAMA 316(15):1575–1582. 10.1001/jama.2016.15217 (2A)
28. Singhal V, Szeto P, VanderMeer TJ, Cagir B (2012) Ventral hernia repair: outcomes change with long-term follow-up. JSLS 16(3):373–379. 10.4293/108680812x13427982377067 (3)
29. Jensen KK, Henriksen NA, Jorgensen LN (2014) Endoscopic component separation for ventral hernia causes fewer wound complications compared to open components separation: a systematic review and meta-analysis. Surg Endosc 28(11):3046–3052. 10.1007/s00464-014-3599-2 (1A)
30. Fernandez Lobato R, Ruiz de Adana Belbel JC, Angulo Morales F, García Septiem J, Marín Lucas FJ, Limones Esteban M (2014) Cost–benefit analysis comparing laparoscopic and open ventral hernia repair. Cir Esp 92(8):553–560. 10.1016/j.ciresp.2013.04.012 (2B)
31. Dietz UA, Fleischhacker A, Menzel S, Klinge U, Jurowich C, Haas K, Heuschmann P, Germer CT, Wiegering A (2017) Risk-adjusted procedure tailoring leads to uniformly low complication rates in ventral and incisional hernia repair: a propensity score analysis and internal validation of classification criteria. Hernia 21(4):569–582. 10.1007/s10029-017-1622-y (2B)
32. Helgstrand F, Rosenberg J, Kehlet H, Jorgensen LN, Bisgaard T (2013) Nationwide prospective study of outcomes after elective incisional hernia repair. J Am Coll Surg 216(2):217–228. 10.1016/j.jamcollsurg.2012.10.013 (2C)
33. Davies SW, Turza KC, Sawyer RG, Schirmer BD, Hallowell PT (2012) A comparative analysis between laparoscopic and open ventral hernia repair at a tertiary care center. Am Surg 78(8):888–892. (3)
34. Liang MK, Berger RL, Li LT, Davila JA, Hicks SC, Kao LS (2013) Outcomes of laparoscopic vs open repair of primary ventral hernias. JAMA Surg 148(11):1043–1048. 10.1001/jamasurg.2013.3587 (3)
35. Juo YY, Skancke M, Holzmacher J, Amdur RL, Lin PP, Vaziri K (2017) Laparoscopic versus open ventral hernia repair in patients with chronic liver disease. Surg Endosc 31(2):769–777. 10.1007/s00464-016-5031-6 (2A)
36. Savitch SL, Shah PC (2016) Closing the gap between the laparoscopic and open approaches to abdominal wall hernia repair: a trend and outcomes analysis of the ACS-NSQIP database. Surg Endosc 30(8):3267–3278. 10.1007/s00464-015-4650-7 (2C)
37. Lee J, Mabardy A, Kermani R, Lopez M, Pecquex N, McCluney A (2013) Laparoscopic vs open ventral hernia repair in the era of obesity. JAMA Surg 148(8):723–726. 10.1001/jamasurg.2013.1395 (2B)
38. Mason RJ, Moazzez A, Sohn HJ, Berne TV, Katkhouda N (2011) Laparoscopic versus open anterior abdominal wall hernia repair: 30-day morbidity and mortality using the ACS-NSQIP database. Ann Surg 254(4):641–652. 10.1097/sla.0b013e31823009e6 (2C)
39. Ng N, Wampler M, Palladino H, Agullo F, Davis BR (2015) Outcomes of Laparoscopic versus Open Fascial Component Separation for Complex Ventral Hernia Repair. Am Surg 81(7):714–719. (2C)
40. Aher CV, Kubasiak JC, Daly SC, Janssen I, Deziel DJ, Millikan KW, Myers JA, Luu MB (2015) The utilization of laparoscopy in ventral hernia repair: an update of outcomes analysis using ACS-NSQIP data. Surg Endosc 29(5):1099–1104. 10.1007/s00464-014-3798-x (2C)
41. Cornette B, De Bacquer D, Berrevoet F (2017) Component separation technique for giant incisional hernia: A systematic review. Am J Surg. 10.1016/j.amjsurg.2017.07.032 (1A)
42. Fox M, Cannon RM, Egger M, Spate K, Kehdy FJ (2013) Laparoscopic component separation reduces postoperative wound complications but does not alter recurrence rates in complex hernia repairs. Am J Surg 206(6):869–874; discussion 874–865. 10.1016/j.amjsurg.2013.08.005 (3)

## Chapter 26. Comparison of hospital stay, return to activity, cost, quality of life, pain, and recurrence after laparoscopic and open ventral and incisional hernia repair

### Virinder Kumar Bansal, Aditya Baksi, Washim F Khan, A Krishna, MC Misra, R Fortelny

**Key questions:**

1. How comparable is the length of hospital stay between laparoscopic IPOM and open sublay repair?
2. How comparable is the time for return to full activities between laparoscopic IPOM and open sublay repair?
3. How comparable are the costs between laparoscopic IPOM and open sublay repair?
4. How comparable is the quality of life between laparoscopic IPOM and open sublay repair?
5. How comparable is the frequency of chronic pain between laparoscopic IPOM and open sublay repair?
6. How comparable is the frequency of recurrences between laparoscopic IPOM and open sublay repair?

**Hospital stay**

**Search terms**

“laparoscopic” AND “open” AND “incisional hernia” OR “ventral hernia” AND “hospital stay”

**Search machines**

Pubmed, Cochrane database, Embase

A systematic search of the available literature was done from August 2012 to September 2017. The search detected 146 articles of which 15 were considered relevant (level 1, 2, or 3). A manual search revealed 7 more articles. Thus, a total of 22 articles have been used in this review. Statements and recommendations were modified accordingly. For the study of the original guidelines, read the publication in “Surg Endosc (2014) 28: page 369–378.”

**Table:** 

The original statements are still valid	

**New statements**

**Table:** 

Level 2C	In patients with chronic liver disease or obesity, length of hospital stay is shorter in LIVHR compared to open repair
Level 3	Length of hospital stay is shorter in both reducible and irreducible ventral and incisional hernias in laparoscopic repair compared to open repair
	The original recommendation is still valid

**New recommendations**

**Table:** 

Grade C	Laparoscopic repair may be preferred in chronic lung disease (CLD), obesity, and for both reducible and irreducible hernias

**Comments**

There are 3 new meta-analyses [1–3], 3 RCTs [4–6], 5 studies based on nationwide database [7, 8–11] and 11 retrospective studies [12–22] comparing hospital stay in laparoscopic and open ventral/incisional hernia repair. In a meta-analysis of 11 RCTs comprising of 1002 patients, Zhang et al. [1] found significantly shorter hospital stay in patients undergoing laparoscopic repair. Although the RCTs by Eker [4] and Rogmark [5] showed no difference in hospital stay, another RCT by Malik et al. [6] showed a considerable difference in length of stay in the two groups (3 days in uncomplicated surgery and 7 days when there was a complication). Colavita et al. [7] have done a nationwide data analysis of more than 18000 patients and found significantly shorter hospital stay in laparoscopic repair (3.5 days) compared to open repair (5.2 days). Savitch et al. [8] have analyzed the data of more than 112 000 patients and found significantly shorter hospital stay in patients repaired laparoscopically. Juo et al. [9] showed that in chronic liver disease patients, LOS was 3.7 days in laparoscopic repair compared to 5 days in open repair. Fekkes et al. [10] found significantly less hospital stay in patients with BMI > 30 undergoing laparoscopic repair. There were 11 retrospective studies comparing more than 42,000 patients undergoing laparoscopic or open hernia repair. Ten of them showed significantly shorter hospital stay in laparoscopic repair.

**Return to activity**

**Search terms**

“laparoscopic” AND “open” AND “incisional hernia” OR “ventral hernia” AND “return to activity”

**Search machines**

Pubmed, Cochrane database, Embase

A systematic search of the available literature was done from August 2012 to September 2017. The search detected 8 articles of which none was considered relevant. A manual search revealed 1 relevant article, which has been used for this review.

**Table:** 

The original statements and recommendations are still valid	

**Comments**

In a meta-analysis by Awaiz et al. [2], only 2 out of 6 RCTs have compared return to work between patients undergoing laparoscopic and open repair of incisional hernia. Although these two RCTs by Olmi et al. [23] and Itani et al. [24] showed earlier return to work after laparoscopic repair, no significant difference was found. As the number of patients analyzed was small, the authors could not reach a meaningful conclusion and hence, further studies are required on this variable.

**Costs**

**Search terms**

“laparoscopic” AND “open” AND “incisional hernia” OR “ventral hernia” AND “cost”

**Search machines**

Pubmed, Cochrane database, Embase

A systematic search of the available literature was done from August 2012 to September 2017. The search detected 78 articles of which 4 were considered relevant (level 1, 2, or 3).

**Table:** 

The original statements are still valid.	

**New statements**

**Table:** 

Level 2C	Laparoscopic ventral hernia repair is more cost effective than open repair
Level 3	Laparoscopic repair of ventral hernias in obese patients is more cost effective than open repair

**Table:** 

No new Recommendations	

**Comments**

Colavita et al. [7] in a study comprising 18,223 patients have found laparoscopic repair of ventral hernia to be more cost effective, which could be attributed to fewer complications (3.97% versus 8.24% in the open group) and shorter hospital stay (3.5 days versus 5.2 days in the open group). Soliani et al. [14] in a retrospective study have found laparoscopic repair of incisional hernias to be more cost effective. Although the costs of operation were higher in the laparoscopic group, the total cost was lower due to reduced cost of hospitalization in laparoscopic surgery. Ecker et al. [25], in a large registry-based study comprising 13,567 patients, found lower cost in LVHR compared to open repair, as a result of lower incidence of reoperation and wound complications in the former. Lee et al. [26] have also found lower hospital charges in laparoscopic repair of ventral hernias in obese patients compared to open repair.

**Quality of Life (QOL)**

**Search terms**

“Laparoscopic” AND “open” AND “incisional hernia” OR “ventral hernia” AND “quality of life”

**Search machines**

Pubmed, Cochrane database, Embase

A systematic search of the available literature was done from August 2012 to September 2017. The search detected 53 articles of which 6 were considered relevant. Manual search detected 1 more relevant article. Thus, a total of 7 articles have been used in this review.

New statements—identical to previous statements, except the following:

**Table:** 

Level 1A	Long-term QOL does not differ between laparoscopic and open incisional/ventral hernia repairs
Level 1 3	A Laparoscopic repair improves overall health-related Quality of Life (HRQoL)
Level 1B	Short-term QOL is better after laparoscopic repair compared to open repair

**New Recommendation**

**Table:** 

Grade B	Laparoscopic repair is recommended compared with open repair when considering HRQoL

**Comments:**

Sosin et al. [27] have done a meta-analysis of 7 RCTs, 6 non-randomized studies, and 1 retrospective study comprising 1202 patients undergoing laparoscopic ventral hernia repair (LVHR). The analysis included health-related QOL (HRQoL) measures like quality of life, function, satisfaction, pain, mental, and emotional well-being. Laparoscopic repair improved overall HRQoL in 6 of 8 studies. Thirteen studies assessed pain and found improved pain scores compared to preoperative levels. The authors did not find any association of long-term pain with LVHR. Functionality improved in 12 studies. Assessment of functionality included time to return to activity. Patient satisfaction was assessed in 4 studies and was found to have favorable results. Satisfaction scores increased progressively over time from surgery. Laparoscopic repair improved mental and emotional well-being in 6 of 7 studies. However, LVHR was not found to have a superior impact on QOL than open ventral hernia repair.

Rogmark et al. [28], in a RCT comprising 133 patients, have found a superior QOL in the short term (< 8 weeks) after laparoscopic midline incisional hernia repair compared to open repair. Pain, movement restriction, and postoperative fatigue were not different but physical function, mental health, and physical composite score favored laparoscopic repair. Seventeen patients in the open group had wound infection compared to 1 in the laparoscopic arm. However, on further follow-up, QOL at 1 year was comparable between the two groups [5]. Event-free recovery was more common after laparoscopic repair (85% vs 65% in open repair) (*p* < 0.01) while there was a non-significant increase in recurrence in laparoscopic repair (*p* < 0.112).

In contrast, Colavita et al. [19], in a retrospective study of 710 patients, found an inferior QOL 1 month after laparoscopic ventral hernia repair compared to open repair. Pain and movement restriction were more in laparoscopic repair (*p* < 0.001). However, QOL (SF-36 scores) at 6 months and 1 year was comparable. Length of hospital stay and wound infection were less in laparoscopic repair but overall complications and recurrence were comparable.

Asti et al. [17], in a cohort study involving 26 open and 28 laparoscopic incisional hernia repairs have found comparable HRQoL in both groups at 1 year. Langbach et al. [29] have conducted a long-term study with a median follow-up of 4 years and found comparable long-term QOL after open and laparoscopic repair.

**Pain**

**Search terms**:

“laparoscopic” AND “open” AND “incisional hernia” OR “ventral hernia” AND “pain”

**Search machines:**

Pubmed/Medline, Embase, Cochrane library

**New publications:**

The search yielded 113 publications, 9 of which were relevant to the search question, and a manual search yielded another 3 papers, resulting in a total of 12 publications used for the review.

**Table:** 

The old statements are still valid	

New Recommendation

**Table:** 

Grade A	Regarding the risk of postoperative pain both techniques—open or laparoscopic—can be recommended equally

**Comments**

**Acute pain**

There are 2 new RCTs describing acute pain following laparoscopic incisional hernia repair. Eker et al. [4] compared 99 laparoscopy patients with 107 open and found no significant difference in pain at 4 weeks follow-up in terms of analgesic use. Rogmark et al. [28] found similar results at 3 weeks.

**Chronic pain**

Schroeder et al. [30] compared a cohort of 40 patients who underwent laparoscopic mesh repair with 46 patients of open repair, and found no difference in chronic (6 month) pain score between the two groups (*p* = 1.0).

**Recurrence**

**Search terms**

“Laparoscopic” AND “open” AND “incisional hernia” OR “ventral hernia” AND “recurrence”

**Search machines**

Pubmed, Cochrane database, Embase

A systematic search of the available literature was done from August 2012 to September 2017. The search detected 332 articles of which 7 were considered relevant (level 1, 2 or 3). A manual search revealed 6 more relevant articles. Thus, a total of 13 articles were reviewed.

**New statement**

**Table:** 

Level 1A	No significant difference in recurrence is found between open and laparoscopic incisional/ventral hernia repairs (stronger evidence)

**New Recommendation**

**Table:** 

Grade A	Regarding the recurrence rate both laparoscopic and open techniques can be recommended equally

**Comments**

There are 3 new meta-analyses [1–3], 2 RCTs [4, 5], and 8 retrospective studies [7, 12–16, 21, 29] comparing recurrence in laparoscopic and open ventral/incisional hernia repair. The largest meta-analysis by Zhang et al. [1] comprising 11 RCTs involving 1002 patients found no significant difference in recurrence rates between laparoscopic (6.99%) and open (4.82%) repairs after a follow-up of 2-35 months. Surgical site infection was significantly lower in the laparoscopic group (2.8% vs 16.2% in the open group), but bowel injury was significantly higher in the laparoscopic group (4.3%) compared to open group (0.81%). Postoperative seroma formation was also comparable. The other two meta-analyses have also found comparable recurrence rates in the two groups. Eker et al. [4] in a randomized trial involving 194 patients found recurrence rates of 18% and 14% in laparoscopic and open repair, respectively, which was not significant. Rogmark et al. [5] did a randomized trial comprising 133 patients and found higher recurrence rates after laparoscopic repair (8.2% vs 1.6% in open repair) but the difference was not statistically significant. Eight retrospective studies [7, 12–16, 21, 29] comprising a total of 1599 patients also found no difference in recurrence rates. Colavita et al. [19] did a nationwide population study on 710 patients and found comparable rates of recurrence in laparoscopic and open repair.

**References** (in parentheses the level of evidence)

1. Zhang Y, Zhou H, Chai Y, Cao C, Jin K, Hu Z (2014) Laparoscopic versus open incisional and ventral hernia repair: a systematic review and meta-analysis. World J Surg 38(9):2233–40. (1A)
2. Awaiz A, Rahman F, Hossain MB, Yunus RM, Khan S, Memon B, et al. (2015) Meta-analysis and systematic review of laparoscopic versus open mesh repair for elective incisional hernia. Hernia 19(3):449–63. (1A)
3. Al Chalabi H, Larkin J, Mehigan B, McCormick P (2015) A systematic review of laparoscopic versus open abdominal incisional hernia repair, with meta-analysis of randomized controlled trials. Int J Surg 20:65–74. (1A)
4. Eker HH, Hansson BME, Buunen M, Janssen IMC, Pierik REGJM, Hop WC, et al. (2013) Laparoscopic vs. open incisional hernia repair: a randomized clinical trial. JAMA Surg 148(3):259–63. (1B)
5. Rogmark P, Petersson U, Bringman S, Ezra E, Osterberg J, Montgomery A (2016) Quality of Life and Surgical Outcome 1 Year After Open and Laparoscopic Incisional Hernia Repair: PROLOVE: A Randomized Controlled Trial. Ann Surg 263(2):244–50. (1 B)
6. Malik AM (2015) Laparoscopic versus open repair of para-umbilical hernia. Is it a good alternative? JPMA J Pak Med Assoc 65(8):865–8 (2B)
7. Colavita PD, Tsirline VB, Walters AL, Lincourt AE, Belyansky I, Heniford BT (2013) Laparoscopic versus open hernia repair: outcomes and sociodemographic utilization results from the nationwide inpatient sample. Surg Endosc 27 (1):109–17 (2C)
8. Savitch SL, Shah PC (2016) Closing the gap between the laparoscopic and open approaches to abdominal wall hernia repair: a trend and outcomes analysis of the ACS-NSQIP database. Surg Endosc 30(8):3267–78. (2C)
9. Juo Y–Y, Skancke M, Holzmacher J, Amdur RL, Lin PP, Vaziri K (2017) Laparoscopic versus open ventral hernia repair in patients with chronic liver disease. Surg Endosc 31(2):769–77. (2B)
10. Fekkes JF, Velanovich V (2015) Amelioration of the effects of obesity on short-term postoperative complications of laparoscopic and open ventral hernia repair. Surg Laparosc Endosc Percutan Tech 25(2):151–7. (2C)
11. Bisgaard T, Kehlet H, Bay-Nielsen M, Iversen MG, Rosenberg J, Jorgensen LN (2011) A nationwide study on readmission, morbidity, and mortality after umbilical and epigastric hernia repair. Hernia 15(5):541–6. (2C)
12. Froylich D, Segal M, Weinstein A, Hatib K, Shiloni E, Hazzan D (2016) Laparoscopic versus open ventral hernia repair in obese patients: a long-term follow-up. Surg Endosc 30(2):670–5. (3B)
13. Sadava EE, Schlottmann F, Bun ME, Rotholtz NA (2016) Laparoscopic incisional hernia repair after colorectal surgery. Is it possible to maintain a mini-invasive approach? Surg Endosc 30(12):5290–4. (3B)
14. Soliani G, De Troia A, Portinari M, Targa S, Carcoforo P, Vasquez G, et al. (2017) Laparoscopic versus open incisional hernia repair: a retrospective cohort study with costs analysis on 269 patients. Hernia 21(4):609–18. (3B)
15. Stipa F, Giaccaglia V, Burza A, Santini E, Bascone B, Picchio M (2013) Incisional hernia: laparoscopic or open repair? Surg Laparosc Endosc Percutan Tech 23(4):419–22. (3B)
16. Tsuruta A, Hirai T, Nakamura M (2014) Retrospective comparison of open versus laparoscopic ventral and incisional hernia repair. Asian J Endosc Surg 7(3):246–50. (3B)
17. Asti E, Sironi A, Lovece A, Bonitta G, Bonavina L (2016) Open Versus Laparoscopic Management of Incisional Abdominal Hernia: Cohort Study Comparing Quality of Life Outcomes. J Laparoendosc Adv Surg Tech A 26(4):249–55. (3B)
18. Kaoutzanis C, Leichtle SW, Mouawad NJ, Welch KB, Lampman RM, Cleary RK (2013) Postoperative surgical site infections after ventral/incisional hernia repair: a comparison of open and laparoscopic outcomes. Surg Endosc 27(6):2221–30. (3B)
19. Colavita PD, Tsirline VB, Belyansky I, Walters AL, Lincourt AE, Sing RF, Heniford BT. (2012) Prospective, long-term comparison of quality of life in laparoscopic versus open ventral hernia repair. Ann Surg 256(5):714–723. (3B)
20. Cassie S, Okrainec A, Saleh F, Quereshy FS, Jackson TD (2014) Laparoscopic versus open elective repair of primary umbilical hernias: short-term outcomes from the American College of Surgeons National Surgery Quality Improvement Program. Surg Endosc 28(3):741–6. (2C)
21. Liang MK, Berger RL, Li LT, Davila JA, Hicks SC, Kao LS (2013) Outcomes of laparoscopic vs open repair of primary ventral hernias. JAMA Surg 148(11):1043–8. (3B)
22. Ahonen-Siirtola M, Rautio T, Ward J, Kössi J, Ohtonen P, Mäkelä J (2015) Complications in Laparoscopic Versus Open Incisional Ventral Hernia Repair. A Retrospective Comparative Study. World J Surg 39(12):2872–7. (3B)
23. Olmi S, Scaini A, Cesana GC, Erba L, Croce E (2007) Laparoscopic versus open incisional hernia repair: an open randomized controlled study. Surg Endosc 21(4):555–9. (1B)
24. Itani KMF, Neumayer L, Reda D, Kim L, Anthony T (2004) Repair of ventral incisional hernia: the design of a randomized trial to compare open and laparoscopic surgical techniques. Am J Surg 188(6A Suppl):22S–29S. (1B)
25. Ecker BL, Kuo LEY, Simmons KD, Fischer JP, Morris JB, Kelz RR (2016) Laparoscopic versus open ventral hernia repair: longitudinal outcomes and cost analysis using statewide claims data. Surg Endosc 30(3):906–15. (3B)
26. Lee J, Mabardy A, Kermani R, Lopez M, Pecquex N, McCluney A (2013) Laparoscopic vs open ventral hernia repair in the era of obesity. JAMA Surg 148(8):723–6. (2C)
27. Sosin M, Patel KM, Nahabedian MY, Bhanot P (2014) Patient-centered outcomes following laparoscopic ventral hernia repair: a systematic review of the current literature. Am J Surg 208(4):677–84. (2A)
28. Rogmark P, Petersson U, Bringman S, Eklund A, Ezra E, Sevonius D, et al. (2013) Short-term outcomes for open and laparoscopic midline incisional hernia repair: a randomized multicenter controlled trial: the ProLOVE (prospective randomized trial on open versus laparoscopic operation of ventral eventrations) trial. Ann Surg 258(1):37–45. (1B)
29. Langbach O, Bukholm I, Benth JŠ, Røkke O (2015) Long term recurrence, pain and patient satisfaction after ventral hernia mesh repair. World J Gastrointest Surg 7(12):384–93. (3B)
30. Schroeder AD, Debus ES, Schroeder M, Reinpold W (2013) Laparoscopic transperitoneal sublay mesh repair: a new technique for the cure of ventral and incisional hernias. Surg Endosc 27(2):648–54. (3B)

## Chapter 27. Do we have an ideal mesh in terms of prevention of adhesions? Are coated meshes really necessary? Are there data to support the manufacturers’ claims of superiority? Is permanent or absorbable barrier preferred?

### F. Köckerling, D. Weyhe, M.C. Misra, J. Kukleta

**Key questions:**

1. Are coated meshes superior to non-coated meshes in terms of formation of adhesions?
2. Are coated meshes superior to non-coated meshes in terms late postoperative occurrence of bowel obstruction?
3. Are there differences between coated polypropylene, ePTFE, coated polyester, or titanium-coated meshes in terms of complications or recurrence rates?

**Search terms**

The following search terms were used: “incisional hernia”; “ventral hernia”; “meshes”; “mesh hernia”; “laparoscopic incisional hernia repair”; “laparoscopic ventral hernia repair”; “hernia repair and meshes.”

**Search machines**

PubMed, Medline, and the Cochrane Library as well as the references lists of the included studies were searched for relevant studies. For the study of the original guidelines, read the publication in “Surg Endosc (2014) 28: page 380–384.”

**New publications**

A total of 7 new articles were identified for inclusion.

New statements—identical to previous statements, except the following:

Statements

**Table:** 

Level 2C	In laparoscopic incisional hernia repair, composite meshes consisting of polypropylene sandwiched between two tissue-separating layers of a bioabsorbable coating have a significantly higher risk for recurrence and chronic pain compared to the other recommended meshes (new statement)
Level 2B	A lightweight monofilament polypropylene mesh with an absorbable hydrogel barrier has in laparoscopic ventral/incisional hernia repair a low complication and recurrence rate (new statement)
Level 2C	The mesh-related complication rate following laparoscopic incisional hernia repair is not higher as following open mesh repair (new statement)
Level 4	A hybrid synthetic/biologic mesh can be used in laparoscopic ventral/incisional hernia repair (new statement).

New recommendations—identical to previous statements, except the following recommendation:

**Recommendation**

**Table:** 

Grade B	For laparoscopic incisional and ventral hernia repair, only meshes approved for implantation in the abdominal cavity should be used (stronger recommendation)

**Comments**

In a randomized controlled trial, two different mesh fixation system concepts were compared in laparoscopic ventral hernia repair: Ventralight ST/SorbaFix vs Physiomesh/Securestrap (1). During the interim analysis, the study was stopped due to safety reasons. They observed five (20%) recurrence in the Physiomesh group in the first 6 months and none in the Ventralight ST group. Additionally, the pain rate was significantly higher in the Physiomesh group after 3 months (*p* < 0.0001) (1).

In a registry-based comparison of meshes in laparoscopic incisional hernia repair (*n* = 5214), a significantly lower recurrence rate was identified for all other meshes (Parietene Composite, Parietex Composite, Symbotex Composite, DynaMesh-IPOM, TiMesh, Ventralight ST) recommended in the guidelines compared with Physiomesh (5.0% vs 12.0%; *p* < 0.001) (2). In the multivariable analysis, the recurrence rate was highly significantly influenced by the Physiomesh (OR = 2.570; 95% CI [2.057–3.210]; *p* < 0.001). Physiomesh also had a negative influence on chronic pain rates (2). In comparison to the other composite meshes on the market, Physiomesh is characterized as the only mesh with a polypropylene layer sandwiched between two tissue-separating layers of a bioabsorbable coating (poliglecaprone 25). Polydioxanone is used as glue to keep all layers together (2).

In a randomized controlled trial comparing open versus laparoscopic incisional hernia repair, the Proceed Composite Mesh used in the laparoscopic arm resulted in a 1-year recurrence rate of 4.9% and patient satisfaction of 93% (3).

In a registry-based comparative study of laparoscopic ventral hernia repair with Ventralight ST versus other meshes, no differences in the recurrence, pain, or quality of life were seen (4). The authors concluded that Ventralight ST is effective and well tolerated in laparoscopic ventral hernia repair (4).

In a prospective multicenter trial (*n* = 63) with the use of a hybrid synthetic/biologic mesh (Zenapro) in laparoscopic ventral and incisional hernia repair, the recurrence rate after 1-year follow-up was 6.8% and the seroma rate 23.7% (5).

In a retrospective cohort study (*n* = 88) of laparoscopic ventral and incisional hernia repair with Parietex Composite vs DynaMesh-IPOM, the recurrence rate was 12.9% vs 3.8% (*p* = 0.20), the obstruction rate secondary to adhesions 0% vs 11.5% (*p* = 0.006), and the seroma/hematoma rate 6.4% vs 0% (*p* = 0.185). The authors concluded that Parietex Composite has a higher recurrence and seroma/hematoma rate, and DynaMesh-IPOM a higher obstruction rate secondary to adhesions (6).

In a registry-based nationwide cohort study including 3242 patients with elective incisional hernia repair, the cumulative incidence of mesh-related complications at five years follow-up was 3.7% following laparoscopic and 5.6% following open mesh repair (7).

**References** (in parentheses the level of evidence)

1. Pawlak M, Hilgers RD, Bury K, Lehmann A, Owczuk R, Smietanski M (2016) Comparison of two different concepts of mesh and fixation technique in laparoscopic ventral hernia repair: a randomized controlled trial Surg Endosc 30:1188–1197. 10.1007/s00464-015-4329-0 (2B)
2. Köckerling F, Simon T, Hukauf M, Hellinger A, Fortelny R, Reinpold W, Bittner R (2018) The Importance of Registries in the Postmarketing Surveillance of Surgical Meshes. Ann Surg 268(6):1097–1104. 10.1097/sla.0000000000002326 (2C)
3. Rogmark P, Petersson U, Brigman S, Ezra E, Österberg J, Montgomery A (2016) Quality of Life and Surgical Outcome 1 Year After Open and Laparoscopic Incisional Hernia Repair Ann Surg 263:244–250. 10.1097/sla.0000000000001305 (1B)
4. Gillion JF, Fromont G, Lepère M, Letoux N, Dabrowski A, Zaranis C, Barrat C, The Hernia-Club Members (2016) Laparoscopic ventral hernia repair using a novel intraperitoneal Lightweight mesh coated with hyaluronic acid: 1-year follow-up from a case–control study using the Hernia-Club registry. Hernia 20:711–722. 10.1007/s10029-016-1501-y (3)
5. Bittner JG, El-Hayek K, Strong AT, Phillips LaPinska M, Yoo JS, Pauli EM, Kroh M (2018) First human use of hybrid synthetic/biologic mesh in ventral hernia repair: a multicenter trial. Surg Endosc 32(3):1123–1130. 10.1007/s00464-017-5715-6. Epub 2017 Jul 19 (3)
6. Tandon A, Shahzad K, Pathak S, Oommen CM, Nunes QM, Smart N (2016) Parietex Composite mesh versus DynaMesh-IPOM for laparoscopic incisional and ventral hernia repair: a retrospective cohort study. Ann R Coll Surg Engl 98(8):568–573. Epub 2016 Sep 23. (3)
7. Kokotovic D, Bisgaard T, Helstrand F (2016) Long-term Recurrence and Complications Associated With Elective Incisional Hernia Repair JAMA. 316(15):1575–1582. 10.1001/jama.2016.15217 (2C)

## Chapter 28. Role of biological/biosynthetic meshes in laparoscopic incisional and ventral hernia repair? Are they advantageous in infected abdominal wall?

### B. Stechemesser, D. Weyhe, B. Ramshaw, F. Köckerling, G. S. Ferzli

**Question:**

1. Is there evidence in using biological meshes or biosynthetic meshes for the repair of ventral hernia in infected area?

**Search terms**

An updated systematic search of the available literature was performed in September 2017. The following search terms were used: “Incisional Hernia,” “Ventral Hernia,” “Laparoscopic Incisional Hernia Repair,” “Laparoscopic Ventral Hernia Repair,” “Biological Meshes,” “Meshes and Hernia Repair,” “Biological Meshes and Hernia Repair,” “Biosynthetic Meshes and Hernia repair.”

**Search machines**

PubMed, Medline, and the Cochran Library as well as the reference lists of the included studies were searched for relevant studies. In addition, the databases were specifically searched for RCTs and clinical reviews on biological implants.

**New publications**

Fifteen new articles met the criteria in this updated review. Data extrapolated from the 15 new pieces of literature support the new statement dealing with biologic mesh and its recurrence rate.

**Statements**

**Table:** 

Level 2b	Regarding short-term results (up to 24 months), open ventral hernia repair can safely be performed with biosynthetic absorbable mesh reinforcement
	Despite contaminated operating field, implantation of a biosynthetic mesh may be safe; however, the long-term durability seems less favorable than previously reported
Level 3	Biological mesh and biosynthetic meshes have similar recurrence rates as synthetic meshes in contaminated ventral hernia repairs and may not be superior as previously thought
Level 4	There is no evidence supporting the use of biologic or biosynthetic meshes in laparoscopic ventral hernia repair

**Recommendation**

**Table:** 

Grade A	In the absence of higher-level evidence, surgeons should carefully balance risk, cost, and benefits in managing contaminated ventral hernia repair
Grade B	The laparoscopic use of biologic or biosynthetic mesh implantation is only recommended in controlled trials

**Comments**

The vast majority of the new literature with respect of implantation of biologic or biosynthetic meshes in contaminated fields is not specific to laparoscopic repair and requires extrapolation of evidence from retrospective, prospective, and systematic reviews which are based nearly exclusively on open repair. In a recently published study regarding this topic, it is concluded that despite the high rate of wound morbidity associated with single-stage reconstruction of contaminated fields, it can safely be performed with biosynthetic absorbable mesh reinforcement (GORE BIO-A Tissue Reinforcement; Flagstaff, Arizona). Though implantation of a biosynthetic mesh in these situations may be safe, the long-term durability seems less favorable than previously reported. Anyway, the use of a biosynthetic alternative to biologic mesh provides a clear opportunity for reducing costs in caring for these complex patients [1].

On the other hand, there has been recent literature by Ditzel et al. [2] and Mulder et al. [3] using biological meshes in experimental studies in rats. These studies allowed the conclusion that although biologic meshes were not superior to synthetic meshes in ventral hernia repair, but they may be of potential use in contaminated environments. Several recent prospective, multicenter, randomized controlled trials (RCT) have examined the use of biological mesh in contaminated fields during open ventral hernia repair. Itani et al. (RICH-Study [4]) examined the clinical outcomes of ventral hernia repair with Strattice mesh, a non-cross-linked, porcine, acellular dermal matrix, in contaminated defects. A total of 80 patients underwent open ventral incisional hernia (VIH) repair and were followed for 2 years. There were 28 infection-related events in 24 patients (30%); however, no tissue matrix required explantation. There were 22 hernia (28%) recurrences. The study concluded that the use of Strattice mesh in the repair of contaminated VIH allowed for successful, single-stage reconstruction in > 70% of patients, but the follow-up time was 24 months only. The Complex Open Bioabsorbable Reconstruction of the Abdominal Wall (COBRA) study [5] examined the recurrence, surgical site infection, and quality of life after repair of contaminated ventral hernias using biosynthetic absorbable mesh. The overall hernia recurrence rate was 17% (*n* = 16), which was almost 11% less than seen in the RICH trial. Postoperative wound infections occurred in 21% of patients, and no patients required explanation of mesh. The authors concluded that biosynthetic absorbable mesh is efficacious and offers an alternative to biological and permanent synthetic meshes in complex situations. Long-term resorbable meshes may be a good alternative technology for complex ventral and incisional hernia repair (VIHR). However, in only 10 patients a laparoscopic repair had been done, and in these patients, the recurrence rate was 40%. But also the COBRA Study had a follow-up of 24 months only. Experimental studies in animals indicated that poly-4-hydroxybutyrate (P4HB) resorbable mesh may support strength restoration of the abdominal wall. Recently, these results could be confirmed by a prospective, multi-institutional study of subjects undergoing retrorectus or onlay VIHR. The authors reported in high-risk VIHR with P4HB mesh (Phasix™ mesh) a positive outcome and low incidence of hernia recurrence, but also in this study the follow-up time was 18 months only [6].

The SIMBIOSE trial [7] is an ongoing phase III RCT comparing the use of biological mesh versus traditional wound care in patients with VIHs. The hypothesis is that the use of a biological mesh will reduce abdominal morbidity, compared to standard wound care without biological mesh. The primary end point is 6-month infectious and/or wound morbidity. The study will continue to collect 3 years of follow-up, which would be the longest follow-up available in studies of biological meshes in the case the authors are successful. Between 2012 and 2018, there were several systematic literature reviews [8–12] examining the use of biologic mesh in contaminated ventral/incisional hernia repairs. Primus and Harris [9] concluded that the cumulative data regarding biologic mesh use in VHRs under contaminated conditions do not support the claim that it is better than synthetic mesh used under the same conditions. Similar to Primus and Harris, Lee et al. [10] stated that the available evidence is limited, but does not support the superiority of biologic over synthetic non-absorbable prosthetics in contaminated fields. Atema et al. [11] found that biologic mesh repair of contaminated defects showed considerable higher rates of surgical site complications and a hernia recurrence rate of 30%. The authors conclude that this review highlights the need for consensus on the role of biologic mesh in abdominal wall. Reports on repairs in clean and clean-contaminated hernias have muddled this debate, as an indication for biologic material in clean hernias is lacking. Although randomized trials are perhaps difficult to conduct, prospective studies or large registries are needed, using uniform definitions and criteria to describe patients, hernia, and surgical characteristics [11].

Regarding the laparoscopic techniques, there are even no data available for any valuable recommendation. All the reviews concluded that while biological mesh may be used in contaminated fields, however, there are not enough data to demonstrate their superiority over synthetic mesh. Especially, regarding a bridging use they are not recommended [12]. In 2015, the Executive Board of the Italian Society for Endoscopic Surgery (SICE) came together to update the guidelines from the European Association of Endoscopic Surgery (EAES) and European Hernia Society (EHS)-endorsed evidence-based Italian Consensus Conference Guidelines from 2010 [13]. The scientific committee selected several topics to be addressed regarding laparoscopic ventral/incisional hernia repair. They based their recommendations after careful and complete literature review with autonomous judgment by the entire panel. The process was supervised by experts in methodology and epidemiology, and two external reviewers to guarantee the most objective, transparent, and reliable work. In regard to biologic mesh used during laparoscopic hernia repair, they came to the weak recommendation that even if their laparoscopic implant is feasible, the use of biologic prosthesis should be restricted to contaminated field in open surgery. Their laparoscopic use is recommended in controlled trials only. Very recently, after analyzing their comparative study over 126 patients, Majumder et al. concluded that the choice of mesh for clean-contaminated/contaminated ventral hernia repair remains debatable. Surprisingly, according to their experience when using synthetic sublay they found a significantly lower wound morbidity and more durable outcomes versus a similar cohort of biologic repairs. The authors discuss that this may be likely secondary to improved bacterial clearance and faster integration of macroporous synthetics used by them. Overall, their findings not only support suitability of synthetic mesh in contaminated settings but also challenge the purported advantage of biologics in clean-contaminated/contaminated ventral hernia repairs [14].

Interval estimates favored biologic matrix repair in contaminated VHR; however, these results were not statistically significant. In the absence of higher-level evidence, surgeons should carefully balance risk, cost, and benefits in managing contaminated ventral hernia repair [16].

**References**

1. Rosen MJ, Krpata DM, Ermlich B, Blatnik JA (2013) A 5-year clinical experience with single-staged repairs of infected and contaminated abdominal wall defects utilizing biologic mesh. Ann Surg 257:991–996 (IIb)
2. Ditzel M, Deerenberg EB, Grotenhuis N, Harlaar JJ, Monkhorst K, Bastiaansen-Jenniskens YM, Jeekel J, Lange JF (2013) Biologic meshes are not superior to synthetic meshes in ventral hernia repair: an experimental study with long-term follow-up evaluation. Surg Endosc 27:3654–3662 (V)
3. Mulder IM, Deerenberg EB, Bemelman WA, Jeekel J, Lange JF (2015) Infection susceptibility of crosslinked and non-crosslinked biological meshes in an experimental contaminated environment. Am J Surg 210:159–166 (V)
4. Itani KM, Rosen M, Vargo D, Awad SS, Denoto G, 3rd, Butler CE (2012) Prospective study of single-stage repair of contaminated hernias using a biologic porcine tissue matrix: the RICH Study. Surgery 152:498–505 (IIb)
5. Rosen MJ, Bauer JJ, Harmaty M, Carbonell AM, Cobb WS, Matthews B, Goldblatt MI, Selzer DJ, Poulose BK, Hansson BM, Rosman C, Chao JJ, Jacobsen GR (2017) Multicenter, Prospective, Longitudinal Study of the Recurrence, Surgical Site Infection, and Quality of Life After Contaminated Ventral Hernia Repair Using Biosynthetic Absorbable Mesh: The COBRA Study. Ann Surg 265:205–211 (IIb)
6. Roth JS, Anthone GJ, Selzer DJ, Poulose BK, Bittner JG, Hope WW, Dunn RM, Martindale RG, Goldblatt MI, Earle DB, Romanelli JR, Mancini GJ, Greenberg JA, Linn JG, Parra-Davila E, Sandler BJ, Deeken CR, Voeller GR (2018) Prospective evaluation of poly-4-hydroxybutyrate mesh in CDC class I/high-risk ventral and incisional hernia repair: 18-month follow-up. Surg Endosc 32:1929–1936 (IIb)
7. Mariette C, Briez N, Denies F, Dervaux B, Duhamel A, Guilbert M, Bruyere E, Robb WB, Piessen G (2013) Use of biological mesh versus standard wound care in infected incisional ventral hernias, the SIMBIOSE study: a study protocol for a randomized multicenter controlled trial. Trials 14:131 (Ib)
8. Cevasco M, Itani KM (2012) Ventral hernia repair with synthetic, composite, and biologic mesh: characteristics, indications, and infection profile. Surg Infect 13:209–215 (III)
9. Primus FE, Harris HW (2013) A critical review of biologic mesh use in ventral hernia repairs under contaminated conditions. Hernia 17:21–30 (IIa)
10. Lee L, Mata J, Landry T, Khwaja KA, Vassiliou MC, Fried GM, Feldman LS (2014) A systematic review of synthetic and biologic materials for abdominal wall reinforcement in contaminated fields. Surg Endosc 28:2531-2546(IIb)
11. Atema JJ, de Vries FE, Boermeester MA (2016) Systematic review and meta-analysis of the repair of potentially contaminated and contaminated abdominal wall defects. Am J Surg 212:982-995.e981(Ia)
12. Köckerling F, Alam NN, Antoniou SA, Daniels IR, Famiglietti F, Fortelny RH, Heiss MM, Kallinowski F, Kyle-Leinhase I, Mayer F, Miserez M, Montgomery A, Morales-Conde S, Muysoms F, Narang SK, Petter-Puchner A, Reinpold W, Scheuerlein H, Smietanski M, Stechemesser B, Strey C, Woeste G, Smart NJ (2018) What is the evidence for the use of biologic or biosynthetic meshes in abdominal wall reconstruction? Hernia 22:249–269 (IIb)
13. Silecchia G, Campanile FC, Sanchez L, Ceccarelli G, Antinori A, Ansaloni L, Olmi S, Ferrari GC, Cuccurullo D, Baccari P, Agresta F, Vettoretto N, Piccoli M (2015) Laparoscopic ventral/incisional hernia repair: updated Consensus Development Conference based guidelines [corrected]. Surg Endosc 29:2463–2484 (IIIa)
14. Majumder A, Winder JS, Wen Y, Pauli EM, Belyansky I, Novitsky YW (2016) Comparative analysis of biologic versus synthetic mesh outcomes in contaminated hernia repairs. Surgery 160:828–838 (III)
15. Kim M, Oommen B, Ross SW, Lincourt AE, Matthews BD, Heniford BT, Augenstein VA (2014) The current status of biosynthetic mesh for ventral hernia repair. Surg Technol Int 25:114–121 (IIIa)
16. Bondre IL, Holihan JL, Askenasy EP, Greenberg JA, Keith JN, Martindale RG, Roth JS, Liang MK (2016) Suture, synthetic, or biologic in contaminated ventral hernia repair. Ventral Hernia Outcomes Collaborative. J Surg Res 200(2):488–94. 10.1016/j.jss.2015.09.007. Epub 2015 Sep 9. (IIA)

## Chapter 29. What happens to synthetic mesh after it is inserted into the body?

### Bruce Ramshaw MD, Michael Fabian MD, Dirk Weyhe MD

**Key questions:**

1. What is the frequency of bowel obstruction in the long-term follow-up compared to open repair?
2. What is the frequency of “meshoma” formation and penetration of the mesh into intra-abdominal organs in the long-term follow-up?

**Acknowledgements** Uwe Klinge added comments and critique.

**Search terms** (publications identified as pertinent to this topic/total publications returned by search):

Mesh explant (0/25), materials characterization of hernia mesh (2/6), hernia mesh explant (0/9), hernia mesh interaction (0/13), hernia mesh analysis (0/39)

The search was performed in October 2011 and a total of two unique publications were returned from this search. Both were clinical studies. A secondary search revealed additional 10 publications pertinent to this topic. Additional information on this topic was searched for on UpToDate.

For the study of the original guidelines, read the publication in “Surg Endosc (2014) 28: page 386–388.”

**Update:**

For this update, additional search terms included the following: abdominal wall hernia, incisional hernia, ventral hernia, synthetic mesh, ingrowth, tissue, shrinkage, explant, and interaction. There were no significant published manuscripts that led to a change in the statements and recommendations. However, there was a systematic review published that encouraged an integrated approach to addressing this issue including surgeons working with biomedical engineers to better understand the complex interaction between the mesh and the human body [1].

Statements

**Table:** 

Level 4	It appears that permanent synthetic (plastic) mesh used for hernia repair is not inert when placed in the patient’s body
Level 4	This biologic interaction is complex, and the effects can be quite variable
Level 4	Mesh alone does not cause chronic pain but may be a contributing factor in addition to other factors that result in chronic pain after inguinal hernia repair

**Recommendations:**

**Table:** 

Grade D	Because currently there is no way to predict the biologic interaction of each patient to each available hernia mesh, the patient should be informed of potential interactions and complications. The complexity and variability of the biologic interaction also would argue against the restricted availability of mesh choices within a hospital or outpatient surgery center, allowing surgeons and patients to have options between a variety of mesh choices
Grade D	Mesh removal may be an appropriate measure in addition to other procedures such as neurolysis and/or neurectomy in an attempt to relieve pain in a patient with chronic groin pain after inguinal hernia repair

**Introduction:**

Hernia repair is one of the most common surgical procedures currently performed. There are over one million hernias repaired in the United States alone each year, and of these, over 150,000 are for incisional hernias. The vast majority of hernias are repaired with a permanent synthetic (plastic) mesh material. We are now only beginning to realize the changes that occur to the mesh and the body after placing mesh into a dynamic biologic organism [1, 2]. The potential advantages of synthetic mesh are that mesh is accessible (easy to manufacture and maintain), consistent (materials are reproducible), durable, and cost effective (less expensive than biological materials).

The first synthetic mesh was placed by Aquaviva in Marseille, France, in 1944, and then reported widely by Dr. Francis Usher in 1958 [3, 4]. For over four decades, it was assumed that the mesh material remained inert after placement in the body. This analysis of current evidence will challenge that belief. Until recently, heavyweight polypropylene was by far the most commonly utilized mesh material. There are now a variety of polypropylene-based meshes with varying densities and pore sizes as well as many meshes produced from other types of polymers. It should be noted that despite synthetic mesh reactions in the body based on current mesh explant analysis, most patients who have had mesh hernia repair have not developed mesh-related complications.

**Research:**

In the late 1990s, and continuing in the last decade, mesh that had been explanted for a variety of reasons was studied by a number of techniques. Histological analysis, scanning electron microscope analysis, chemical analysis, infrared spectroscopy, differential scanning calorimetry, thermogravimetric analysis, and compliance testing have all been used to test and examine synthetic mesh, mostly from prior abdominal wall hernia repair, but also after pelvic floor reinforcement [5].

The meshes have been found to undergo changes as a result of the body’s defense against foreign objects, as well as complex changes due to chemical attack on the polymer structure [6]. There have also been many complications related to mesh hernia repair and the result of this mesh–body interaction may be a contributing factor to these complications. Complications related to mesh interaction with the body include recurrence due to mesh contraction and/or migration, mesh erosion into viscera and/or through skin, chronic pain, functional issues due to lack of mesh compliance, acute and delayed mesh infection, acute and chronic inflammatory reactions including chronic active seroma, and rare systemic symptoms, such as flu-like symptoms, potentially related to synthetic mesh. The variety of methods used to study mesh after explantation from the body are now presented.

**Histology:**

At the cellular level, the body will attempt to wall-off, digest, or expel the foreign material. Cellular immunity is critical for survival, yet it creates problems in some (but not all) hernia patients. Polypropylene seems to have the greatest inflammatory reaction of the synthetic meshes, but this appears to decrease over time [7].

Neutrophils, lymphocytes, macrophages, and foreign-body giant cells are stimulated upon injury (surgery) and implantation of mesh material. These cells release enzymes and oxidants to degrade the foreign body, in this case the mesh [8]. Study of the mesh has shown oxidative breakdown, in addition to encasement with inflammatory cells. Lymphocytes and foreign-body giant cells are present, and these can bath the mesh in a continuous environment of oxidants, while progressively encasing the mesh in a fibrous scar that can become increasingly rigid. This may be a contributing factor to chronic and in some cases debilitating pain [9].

The foreign body response has been classified as having four distinct phases: acute inflammation, chronic inflammation, foreign body reaction with development of granulation tissue, and fibrosis [8]. Heavyweight polypropylene meshes exhibit more collagen deposition and fibrosis, while lightweight meshes exhibit minimal fibrotic tissue with better neovascularization around the mesh [10].

The oxidants released by lysosomes can create superoxide anions, as well as hydrogen peroxide and hypochlorous acid [11]. Polypropylene has been shown to undergo chain scission, and overall degradation with fissures, microcracks, build-up of hydroxyl and carbonyl groups on the surface of the material, changes in thermal properties (see below), and changes in mechanical properties such as embrittlement and reduced compliance.

There has also been discussion that the meshes generally shrink due to the above changes. But this contraction or shrinkage appears to be a very complex and irregular process. Coda et al. studied multiple types of mesh and discovered that the explanted mesh pore sizes could have expanded up to 58% as well as shrunk by 40% [12].

**Scanning Electron Microscopy:**

Most micrographs have demonstrated changes to the polypropylene mesh that include microcracks in the transverse direction, as well as peeling of the top layer of fibers [10]. Other changes included superficial or deep flaking, and fractures in the threads of varying lengths and depths [5]. Interestingly, polyethylene terephthalate did not appear degraded in two separate studies [5, 13]. These findings are contrary to other reports on degradation of vascular grafts, and much more study of this complex biologic interaction is needed.

**Fourier Transform Infrared Spectroscopy:**

FTIR Spectroscopy is a spectroscopic technique widely used to facilitate determination of chemical functional groups by their absorption frequency. Both Clave and Cozad in separate papers in 2010 examined multiple types of mesh [5, 14]. They discovered that in virtually all types of synthetic mesh, peaks representing hydroxyl and carbonyl groups were present. This has even been noted in ePTFE, one of the meshes thought to be the least affected by alterations.

This indicated a chemical breakdown of the “inert” mesh that has potential implications for the strength of the polymer. Many of the hydrocarbon propylenes depend on Van Der Waals forces, and the alteration of the chemical groups can weaken these bonds. The overall effect may explain the changes in mesh seen in the tests mentioned below.

**Differential Scanning Calorimetry:**

This test measures melting temperature and heat of fusion in materials and has been tested in a variety of explanted meshes. This showed a shift toward lower melting temperature and broader melting peak. The clinical implication is not clear but demonstrates a change in the physical properties of the mesh.

**Thermogravimetric Analysis:**

This measures weight loss of the material versus a pristine piece of mesh. This was lower for all mesh tested. This is now intuitive, as the material has been assaulted by the body, exposed to oxidative forces, and broken down chemically. This would also explain the mechanical failure of some lightweight meshes, which have been designed to lessen the host response with fibrosis and scarring but sacrifice strength to achieve this.

**Compliance Testing:**

This measures the mean value of work to bend the mesh in half using a constant force. Nearly all materials tested, even after removing all organic material, required more work and were less compliant than the pristine control mesh. However, this compliance testing revealed tremendous variability between explant samples [9, 10].

**Clinical Application:**

The most concerning complication that may be related to mesh interaction in the body is chronic groin pain after inguinal hernia repair, or inguinodynia. Chronic pain is a complex problem and does occur in patients who undergo a non-mesh inguinal hernia repair [15]. Chronic pain also occurs after a number of other types of surgical procedures that do not involve implantation of a medical device, such as mastectomy and thoracotomy. However, there is evidence that removal of mesh in patients with chronic pain after hernia repair can contribute to relief of pain [16, 17]. A recent explant study suggested that there is an increase of nerve density in the meshes that were explanted from patients suffering from chronic pain compared with patients without chronic pain who had mesh removed during a procedure for repair of a recurrent inguinal hernia [18].

**Summary:**

Since the early 1990s, a diverse group of individuals, including materials engineers, chemical engineers, pathologists, device company representatives, and surgeons have made early attempts to begin to understand the changes that occur after mesh implantation in human beings. Animal experiments have not been able to show the long-term consequences of foreign body implantation into biologic organisms. The host response is variable, and we have only begun to realize the individualization that will be needed to find the best mesh for a particular cluster of individuals. There will likely be groups of patients that will have a better outcome with certain types of mesh as well as certain groups of patients who will be at risk for increased mesh-related complications with certain types of mesh. To attempt to define these groups, an evolved understanding of clinical research, based on principles of complex systems science, will likely be needed.

**References** (in parentheses the level of evidence)

1. Todros S, Pavan PG, Pachera P, Natali AN (2017) Synthetic surgical meshes used in abdominal wall surgery: Part II- Biomechanical aspects. J Biomed Mater Res B Appl Biomater 105(4):892–903 (1)
2. UpToDate Jun 11, 2010. Reconstructive Materials used in surgery: Classification and host response. (5)
3. Usher FC, Gannon JP (1959) A new plastic mesh for replacing tissue defects, experimental studies. AMA Arch Surg. 78(1):131–7 (5)
4. Usher FC, Ochsner J, Tuttle LLD Jr. (1958) Use of Marlex in incisional hernias Am Surg 24(12):969–74 (5)
5. Clave A, Yahi H, Hammou JC, Montanari S, Gounon P, Clave H (2010) Polypropylene as a reinforcement in pelvic surgery is not inert: comparative analysis of 100 explants. Int Urogynecol J 21:261–270 (4)
6. Klinge U, Klosterhalfen B, Muller M, Schumpelick V (1999) Foreign body reaction to meshes used for the repair of abdominal wall hernias Eur J Surg 165: 665–673 (4)
7. Klosterhalfen B, Junge K, Hermanns B, Klinge U (2002) Influence of implantation interval on the long-term biocompatibility of surgical mesh Br J Surg 89(8): 1043–1048 (4)
8. Offner FA (2004) Pathophysiology and pathology of the foreign-body reaction to mesh implants. In: Schumpelick V, Nyhus LM, eds. Meshes: Benefits and Risks. New York, NY: Springer; (2004): 161–169 (5)
9. Costello CR, Bachman SL, Grant SA, Cleveland DS, Loy TS, Ramshaw BJ (2007) Characterization of heavyweight and lightweight polypropylene prosthetic mesh explants from a single patient Surg Innov 14(3):168–176 (4)
10. Costello CR, Bachman SL, Ramshaw BJ, Grant SA (2007) Materials characterization of explanted polypropylene hernia meshes J of Biomedical Materials Research Part B: Applied Biomaterials 10.1002/jbmb44-49 (4)
11. Ratner B, Hoffman AS, Schoen FJ, Lemons JE (1996) Biomaterials Science. San Diego, CA: Academic Press 243–254 (5)
12. Coda A, Bendavid R, Botto-Micca F, Bossotti M, Bona A (2003) Structural alterations of prosthetic meshes in humans. Hernia 7(1):25–34 (4)
13. Bracco P, Brunella V, Trossarelli L, Coda A, Botto-Micca F (2005) Comparison of polypropylene and polyethylene terephthalate (Dacron) meshes for abdominal wall hernia repair: A chemical and morphological study. Hernia 9: 51–55 (4)
14. Cozad MJ, Grant DA, Bachman SL, Grant DN, Ramshaw BJ, Grant SA (2010) Materials characterization of explanted polypropylene, polyethylene terephthalate, and expanded polytetrafluoroethylene composites: Spectral and thermal analysis. J of Biomedical Materials Research B: Applied Biomaterials 94(2) 455–462 (4)
15. Öberg S, Andresen K, Klausen TW, Rosenberg J. (2018) Chronic pain after mesh versus nonmesh repair of inguinal hernias: A systematic review and a network meta-analysis of randomized controlled trials. Surgery 163(5):1151–1159 (1)
16. Zwaans WA, Vehagen T, Roumen RM, Scheltinga MR. (2015) Factors determining outcome after surgery for chronic pain following Lichtenstein hernia repair. World J Surg 39(11):2652–2662. (4)
17. Ramshaw B, Vetrano V, Jagadish M, Forman B, Heidel E, Mancini M. (2017) Laparoscopic approach for the treatment of chronic groin pain after inguinal hernia repair. Surg Endosc 31(12):5267–5274. (4)
18. Bendavid R, Lou W, Grishkan D, Kock A, Petersen K, Morrison J, Iakovlev V. (2016) A mechanism of mesh-related post-herniorrhaphy neuralgia. Hernia 20:357–365. (4)

## Chapter 30. Open abdominal surgery and stoma surgery: indications for prophylactic mesh implantation and risk reduction strategies

### Qiyuan Yao, D. Weyhe, G. Woeste

**Key questions:**

1. Is routine use of prophylactic mesh for closure of large abdominal incisions beneficial in terms of prevention of incisional hernias in the long term in comparison to carefully done suture closure?
2. Does routine use of prophylactic mesh in stoma creation lead to a lower rate of parastomal hernias compared to traditional stoma creation without mesh?
3. Does prophylactic mesh implantation in stoma creation lead to more frequent problems of bowel movements (due to stenosis) compared to traditional stoma creation without mesh?

**Search terms**

((indication AND prophylactic AND mesh)) OR (“Hernia, Ventral/prevention and control”[Mesh] OR “Hernia/prevention and control”[Mesh] OR incisional hernia AND (prevention OR prophylactic) OR abdominal wall hernia AND (prevention OR prophylactic) OR “Hernia, Abdominal/prevention and control”[mesh]) OR hernia prevention OR hernia prophylaxis OR prophylactic mesh OR mesh implantation OR (mesh AND “risk reduction”[tiab]) AND (randomized controlled trial[pt] OR controlled clinical trial[pt] OR randomized[tiab] OR placebo[tiab] OR clinical trials astopic[mesh] OR randomly[tiab] OR trial[ti] NOT (animals[mh] NOT humans[mh]))

A systematic search of the literature was performed using Medline, PubMed, Cochrane library, and reference lists for the time period of June 2012 to June 2017 to update.

The search produced 304 articles; with 179 randomized controlled trial and 70 systematic review. For the study of the original guidelines, read the publication in “Surg Endosc (2014) 28: page 388–391.”

**New statements:**

The original are still valid.

**Table:** 

Level 1	A significant reduction in incidence of incisional hernia in patients undergoing elective midline laparotomy was achieved with prophylactic mesh reinforcement in onlay position compared with sublay mesh reinforcement and primary suture only
Level 1	Prophylactic mesh application at the time of primary colostomy formation is a promising method for the prevention of parastomal herniation
Level 1	Prophylactic mesh placement reduces the rate of incisional hernia in risk groups with morbid obesity, aortic aneurysm, or colorectal surgery
Level 3	Prophylactic mesh in the closure of emergency midline laparotomies is feasible for the prevention of incisional hernia
Level 2	Extraperitoneal colostomy was observed to lead to a lower rate of parastomal hernia and stoma prolapse versus transperitoneal
Level 2	Use of a resorbable synthetic mesh during emergency ostomy formation showed no significant preventive effect on formation of parastomal hernia after 1 year

**Recommendations**

**Table:** 

Grade B	Prophylactic onlay mesh reinforcement has the potential to become the standard treatment for high-risk patients undergoing midline laparotomy
Grade B	There is no relevant difference between midline and transverse incisions regarding the incidence for incisional hernia formation
Grade C	A prophylactic mesh could be used in the closure of emergency midline laparotomies in high-risk groups
Grade A	A prophylactic mesh should be placed at the primary stoma operation
Grade B	Extraperitoneal colostomy is more effective and safer for end colostomies compared to transperitoneal
Grade B	The resorbable synthetic mesh has no advantage during emergency ostomy formation

Indication for prophylactic mesh implantation for open abdominal surgery

As indicated in the guidelines of 2014, prophylactic mesh placement reduces the rate of incisional hernia in high-risk groups with morbid obesity or aortic aneurysm. Subsequent publications supported this conclusion.

Abo-Ryia MH et al. published an RCT [1] in 2013 and included 64 morbidly obese patients who underwent open bariatric surgery, which showed that the using of prophylactic mesh during wound closure in open bariatric surgery was safe and effective. A systematic review and meta-analysis [2] by Dasari M et al. combined seven studies and showed that prophylactic mesh during open bariatric surgery appears to be beneficial in reducing postoperative incisional hernia without significant increase in surgical site infection, seroma, or wound leakage. In 2013, Muysoms FE et al. reported a multicenter randomized trial[3] including 120 patients who underwent elective abdominal aortic aneurysm repair. Prophylactic mesh was safe and effectively prevents the development of incisional hernia. Prophylactic mesh can also be used in the setting of colorectal surgery. García-Ureña MÁ et al. conducted an RCT [4] in which patients undergoing colorectal procedure (both elective and emergency) showed a decreased incidence of incisional hernia in patients reinforced with prophylactic large-pore polypropylene mesh without additional morbidity. The incidence of incisional hernia was 17 of 54 (31.5%) in the control group and 6 of 53 (11.3%) in the study group (*p* = 0.011). A prospective cohort study[5] conducted by Argudo N et al. involving 235 patients showed statistically significant reduction in incisional hernia incidence in high-risk groups in which the algorithm was correctly applied (10.2 vs 46.3%; *p* = 0.0001; OR 7.58; 95% CI 3.8–15). We conclude that prophylactic mesh placement reduces the rate of incisional hernia in high-risk groups with morbid obesity, aortic aneurysm, or colorectal surgery.

As to mesh position, a controlled, prospective, randomized, and blind study [6] conducted by Caro-Tarrago A et al. included 160 patients and showed that the prophylactic supra-aponeurotic mesh prevents incisional hernia independently of other factors. A multicenter, double-blind RCT [7] at 11 hospitals in Austria, Germany, and the Netherlands included 498 patients; of which after 2 years’ follow-up, 92 patients were identified with an incisional hernia. There are 33 (30%) who were allocated primary suture only, 25 (13%) who were assigned onlay mesh reinforcement, and 34 (18%) who were assigned sublay mesh reinforcement (onlay mesh reinforcement vs primary suture, OR 0.37, 95% CI 0.20–0.69; *p* = 0.0016; sublay mesh reinforcement vs primary suture, 0.55, 0.30–1.00; *p* = 0.05). We conclude that onlay mesh reinforcement may be more effective than sublay mesh in the prevention of incisional hernia in high-risk patients undergoing midline including laparotomy.

As to emergency surgery, the RCT by Garcia-Ureno et al. described that prophylactic mesh could be used in emergency colorectal surgery [4]. In a retrospective study [8] including 266 patients who underwent an emergency midline laparotomy, 36 cases of incisional hernia (24%) were diagnosed: 33 (33%) in the suture group, and only three cases (5.9%) in the mesh group (*p* = 0.0001). There were no differences regarding surgical site infection rates (17.9% Group suture vs 26.3% Group mesh; *p* = 0.13) or postoperative mortality (13.7% Group suture vs 18.3% Group mesh; *p* = 0.346). Based on the evidence collected, we can conclude that the use of a partially absorbable, lightweight large-pore prophylactic mesh in the closure of emergency midline laparotomies is feasible for the prevention of incisional hernia without adding a substantial rate of morbidity to the procedure, even if high contamination or infections are present.

**Indication for prophylactic mesh implantation for stoma formation.**

As indicated in the guidelines of 2014, prophylactic mesh placement in primary stoma formation reduces the rate of parastomal hernia without increasing morbidity, although this is based on small patient populations.

Six RCTs [9–14] have been published since 2014. A systematic review and meta-analysis [15] of prophylactic mesh for parastomal hernia including 7 RCTs (432 patients) showed it is safe and effective in preventing parastomal hernia. Mesh reduced the incidence of clinically detected parastomal hernia (10.8% vs 32.4%; *p* = 0.001). No increase in the incidence of stoma-related complications was observed with the use of prophylactic mesh. A systematic review and meta-analysis [16] of extraperitoneal versus transperitoneal colostomy for preventing parastomal hernia including 10 studies (2 RCTs and 8 retrospective studies) showed the extraperitoneal colostomy led to significantly lower parastomal hernia rates [22 of 347 (6.3%) for extraperitoneal versus 125 of 701 (17.8%) for transperitoneal; risk ratio = 0.36 (95% CI 0.21–0.62); *I*2 = 26%; *p* < 0.001] and significantly lower stoma prolapse rates [2 of 185 (1.1%) for extraperitoneal versus 13 of 179 (7.3%) for transperitoneal; risk ratio = 0.21 (95% CI 0.06–0.73); *I*2 = 0%; *p* = 0.01]. Differences in stoma necrosis were not significant. We conclude that extraperitoneal colostomy is more effective and safe for end colostomies versus transperitoneal. However, the extraperitoneal route of stoma placement warrants further investigation.

As to the emergency surgery, a cohort study [17] including 109 patients undergoing emergency surgery with the formation of ileostomy or colostomy, in whom retromuscular slowly resorbable synthetic mesh was placed for reinforcement, demonstrated that the incidences of parastomal hernia at 1 year for the control and the mesh group were 8 and 7%, respectively (*p* = 0.424). Based on the previous information, we can conclude that the use of a resorbable synthetic mesh during emergency ostomy formation showed no significant preventive effect on the formation of parastomal hernia.

**Suture technique**

As indicated in the guidelines of 2014, fascia closure with a continuous suture technique using slowly resorbable suture material reduces the incidence for incisional hernia after elective median laparotomy significantly. Achieving a suture length-to-wound length ratio of 4 or more significantly reduces the incidence of incisional hernia after midline incision [18]. The STITCH Trial recently confirmed the benefit of the small-bit technique in prevention of incisional hernia, reducing the rate of hernia from 21 to 13% (*p* = 0.02) [19]. Another RCT [20] comparing long-stitch technique and short-stitch technique on the occurrence of incisional hernia after elective abdominal wall closure is ongoing, and, we are looking forward to the result.

**References** (in parentheses the level of evidence)

1. Abo-Ryia MH, El-Khadrawy OH, Abd-Allah HS (2013) Prophylactic preperitoneal mesh placement in open bariatric surgery: a guard against incisional hernia development. Obes Surg 23(10):1571–4. (1B)
2. Dasari M, Wessel CB, Hamad GG (2016) Prophylactic mesh placement for prevention of incisional hernia after open bariatric surgery: a systematic review and meta-analysis. Am J Surg 212(4):615–622. (1A)
3. Muysoms FE, Detry O, Vierendeels T, Huyghe M, Miserez M, Ruppert M, Tollens T, Defraigne JO, Berrevoet F (2016) Prevention of Incisional Hernias by Prophylactic Mesh-augmented Reinforcement of Midline Laparotomies for Abdominal Aortic Aneurysm Treatment: A Randomized Controlled Trial. Ann Surg 263(4):638–45. (1B)
4. García-Ureña MÁ, López-Monclús J, Hernando LA, Montes DM, Valle de Lersundi AR, Pavón CC, Ceinos CJ, Quindós PL (2015) Randomized controlled trial of the use of a large-pore polypropylene mesh to prevent incisional hernia in colorectal surgery. Ann Surg 261(5):876–81. (1B)
5. Argudo N, Iskra MP, Pera M, Sancho JJ, Grande L, López-Cano M, Pereira JA (2017) The use of an algorithm for prophylactic mesh use in high risk patients reduces the incidence of incisional hernia following laparotomy for colorectal cancer resection. Circ Esp 95(4):222–228. (2B)
6. Caro-Tarrago A, Olona Casas C, Jimenez Salido A, Duque Guilera E, Moreno Fernandez F, Vicente Guillen V (2014) Prevention of incisional hernia in midline laparotomy with an onlay mesh: a randomized clinical trial. World J Surg 38(9):2223–30. (1B)
7. Jairam AP, Timmermans L, Eker HH, Pierik REGJM, van Klaveren D, Steyerberg EW, Timman R, van der Ham AC, Dawson I, Charbon JA, Schuhmacher C, Mihaljevic A, Izbicki JR, Fikatas P, Knebel P, Fortelny RH, Kleinrensink GJ, Lange JF, Jeekel HJ; PRIMA Trialist Group (2017) Prevention of incisional hernia with prophylactic onlay and sublay mesh reinforcement versus primary suture only in midline laparotomies (PRIMA): 2-year follow-up of a multicentre, double-blind, randomised controlled trial. Lancet 390(10094):567–576. (1A)
8. Argudo N, Pereira JA, Sancho JJ, Membrilla E, Pons MJ, Grande L (2014) Prophylactic synthetic mesh can be safely used to close emergency laparotomies, even in peritonitis. Surgery 156(5):1238–44. (3B)
9. Vierimaa M, Klintrup K, Biancari F, Victorzon M, Carpelan Holmström M, Kössi J, ellokumpu I, Rauvala E, Ohtonen P, Mäkelä J, Rautio T (2015) Prospective, randomized study on the use of a prosthetic mesh for prevention of parastomal hernia of permanent colostomy. Dis Colon Rectum 58:943–949 (1B)
10. Lambrecht JR, Larsen SG, Reiertsen O, Vaktskjold A, Julsrud L, Flatmark K (2015) Prophylactic mesh at end-colostomy construction reduces parastomal hernia rate: a randomized trial. Colorectal Dis 17(10):O191–7. (1B)
11. López-Cano M, Serra-Aracil X, Mora L, Sánchez-García JL, Jiménez-Gómez LM, Martí M, Vallribera F, Fraccalvieri D, Serracant A, Kreisler E, Biondo S, Espín E, Navarro-Soto S, ArmengolCarrasco M (2016) Preventing parastomal hernia using a modified Sugarbaker technique with composite mesh during laparoscopic abdominoperineal resection: a randomized controlled trial. Ann Surg 264:923–928 (1B)
12. Fleshman JW, Beck DE, Hyman N, Wexner SD, Bauer J, George V, PRISM Study Group (2014) A prospective, multicenter, randomized, controlled study of non-cross-linked porcine acellular dermal matrix fascial sublay for parastomal reinforcement in patients undergoing surgery for permanent abdominal wall ostomies. Dis Colon Rectum 57:623–631 (1B)
13. Brandsma HT, Hansson BM, Aufenacker TJ, van Geldere D, van Lammeren FM, Mahabier C, Steenvoorde P, de Vries Reilingh TS, Wiezer RJ, de Wilt JH, Bleichrodt RP, Rosman C (2016) Prophylactic mesh placement to prevent parastomal hernia, early results of a prospective multicentre randomized trial. Hernia 20:535–541(1B)
14. Figel NA, Rostas JW, Ellis CN (2012) Outcomes using a bioprosthetic mesh at the time of permanent stoma creation in preventing a parastomal hernia: a value analysis. Am J Surg 203:323–326 (IV)
15. Chapman SJ, Wood B, Drake TM, Young N, Jayne DG (2017) Systematic Review and Meta-analysis of Prophylactic Mesh During Primary Stoma Formation to Prevent Parastomal Hernia. Dis Colon Rectum 60(1):107–115. (1A)
16. Kroese LF, de Smet GH, Jeekel J, Kleinrensink GJ, Lange JF (2016) Systematic Review and Meta-Analysis of Extraperitoneal Versus Transperitoneal Colostomy for Preventing Parastomal Hernia. Dis Colon Rectum 59(7):688–95. (1B)
17. Lykke A, Andersen JFB, Jorgensen LN, Mynster T (2017) Prevention of parastomal hernia in the emergency setting. Langenbecks Arch Surg 402(6):949–955. 10.1007/s00423-017-1596-3. Epub Jun 14Jun (2B)
18. Millbourn D, Cengiz Y, Israelsson LA. (2009) Effect of stitch length on wound complications after closure of midline incisions: a randomized controlled trial. Arch Surg 144(11):1056–9 (1B)
19. Deerenberg EB, Harlaar JJ, Steyerberg EW, Lont HE, van Doorn HC, Heisterkamp J, Wijnhoven BP, Schouten WR, Cense HA, Stockmann HB, Berends FJ, Dijkhuizen FPH, Dwarkasing RS, Jairam AP, van Ramshorst GH, Kleinrensink GJ, Jeekel J, Lange JF (2015) Small bites versus large bites for closure of abdominal midline incisions (STITCH): a double-blind, multicentre, randomised controlled trial. Lancet. 26;386(10000):1254–1260 (1B)
20. Fortelny RH, Baumann P, Thasler WE, Albertsmeier M, Riedl S, Steurer W, Kewer JL, Shamiyeh A (2015) Effect of suture technique on the occurrence of incisional hernia after elective midline abdominal wall closure: study protocol for a randomized controlled trial. Trials 15;16:52. (1B)

## Chapter 31. NOTES and Single-Port Surgery: Is there currently any role in Ventral Hernia Repair today?

### Davide Lomanto, R. Fortelny, Hrishikesh P. Salgaonkar

**a. Natural orifice transluminal endoscopic surgery (NOTES) for Ventral/Incisional hernia repair**

**Search terms:**

Animal, Colon, Hernia, Ventral, Prosthesis Implantation, Surgical Mesh, Suture Techniques/instrumentation, Swine, Endoscopy/methods, Endoscopy/trends, Endoscopy Gastrointestinal Methods, Surgical Procedures, Minimally invasive, Swine, Natural Orifice Transluminal Endoscopic Surgery, Surgical Wound Infection/prevention, Gastric Lavage, Laparoscopy, Ventral hernia repair, Incisional Hernia Repair, Umbilical Hernia Repair, Surgical Procedures, Minimally invasive, Laparoscopy Laparoscopic Ventral/Incisional hernia Repair

**Searching machines:**

PubMed, Embase, Medline, and Cochrane Library (2003–2017) were searched for studies for potential inclusion.

For the study of the original guidelines, read the publication in “Surg Endosc (2014) 28: page 396–399.”

**New publications:**

In addition to studies included in the published guidelines, total of 5 new studies were found relevant and included in the update. One Level 2 study, three Level 4 study, and one level 5 study, which are supplementing our knowledge regarding the use of NOTES for ventral and incisional hernia repair were analyzed.

**New Statement:** Identical to previous statement except the following:

**Table:** 

Level 4	NOTES technique for ventral hernia repair is feasible providing improved cosmesis and possible reduction in port-site hernia in experimental groups
Level 2b	Surgically prepped vaginal canal can be a sterile conduit for insertion of polypropylene mesh for transvaginal ventral hernia repair

**Recommendations**: No new recommendations

**Comments:**

NOTES platform is a new and evolving technology. Most literature is based on animal studies with very few studies involving humans, mostly case reports. The prime objectives in developing NOTES platform are better cosmesis with equivalent or improved clinical results. The main concerns of using NOTES in hernia repair are prosthesis delivery, prevention of prosthesis contamination, and recurrence rates. Wood et al. in a case report, performed a transvaginal ventral hernia repair for umbilical port-site hernia following a laparoscopic cholecystectomy [1]. 10% povidone–iodine solution was used to sterilize the vagina. A SILS port was used for access and a specimen retrieval bag to deploy the mesh into the peritoneal cavity. Absorbable tackers were used to fix the mesh to anterior abdominal wall. Operative time was 103 min and no complications were reported at 9-month follow-up. Bruna et al., similarly, reported a case of recurrent epigastric hernia, using a combined hybrid technique, where the 12-mm optical trocar and 5-mm assistant trocar were inserted through the vagina and two additional 5-mm abdominal working ports were used to perform adhesiolysis [2]. They inserted mesh directly through the 12-mm trocar without any protection. No complications reported at 1-month follow-up. They suggested that transvaginal approach may possibly avoid abdominal incisions with better cosmesis, structural benefit, and possibly less pain. In 2013, Panait et al. presented their series of 107 NOTES operations, which included 2 ventral hernia repairs [3]. The mean operative time was 90 min, with no conversions or major complications reported.

With the objective of achieving better and safe mesh deployment, Earle et al. in a study on swine explants studied two methods of transgastric mesh deployment [4]. A modified esophageal stent delivery system to protect the mesh was compared with unprotected delivery of prosthesis through gastrotomy during transgastric deployment technique. They reported that use of the modified stent delivery system nearly eliminates prosthetic contamination when placed via a transgastric approach. Bates et al. demonstrated that a prepped vaginal canal is a sterile conduit for ventral hernia mesh deployment [5]. 10 consecutive patients undergoing laparoscopic surgery for benign gynecological disease were enrolled. In all patients, a polypropylene monofilament mesh was inserted into the vagina before and after surgical preparation with 10% povidone–iodine. As a control, they inserted similar mesh through prepped laparoscopic port site. They reported that prepped vaginal conduits are more sterile than prepped skin incision and unprepped vaginal conduits based on culture reports of the inserted meshes.

It is still early to derive any definitive conclusions on the safety and feasibility on the use of NOTES platform for ventral or incisional hernia repair. Potential risks of mesh infection, risk of visceral injury, along with patient acceptability, longer operative times, and learning curve are genuine concerns. Further randomized control trials with longer follow-up and larger patient group are required before making recommendations for the use of NOTES in hernia repair.

**References** (in parentheses graduation of evidence)

1. Wood SG, Panait L, Bell RL, Duffy AJ, Roberts KE (2013) Pure transvaginal umbilical hernia repair. Surg Endosc 27(8):2966. Epub 2013 Feb 23. PMID: 23426091 (4)
2. Bruna M, Noguera J, Martinez I, Oviedo M (2013) Eventroplastia transvaginal hibrida. Cir Esp 91:539–541. 10.1016/j.ciresp.2013.02.022. (4)
3. Panait L, Wood SG, Bell RL, Duffy AJ, Roberts KE (2013) Transvaginal natural orifice transluminal endoscopic surgery in the morbidly obese. Surg Endosc 27(7):2625–9. Epub 2013 Jan 26. PMID: 23355168 (4)
4. Earle DB, Romanelli JR, McLawhorn T. Omotosho P, Wu P, Rossini C, Swayze H, Desilets DJ (2012) Prosthetic mesh contamination during NOTES (^®^) transgastric hernia repair: a randomized controlled trial with swine explants. Hernia 16: 689. 10.1007/s10029-012-0944-z. (5)
5. Bates AT, Capes T, Krishan R, LaBombardi V, Pipia G, Jacob BP (2014) The prepped vaginal canal may be a sterile conduit for ventral hernia mesh insertion: a prospective comparative study. Surg Endosc 28(3):886–90. PMID: 24232132. (2b)

**b. Single-port surgery:**

**Search terms:**

Hernia, Ventral hernia, Umbilical hernia, Incisional hernia, Surgical Mesh, Suture Techniques, Surgical instrumentation, Ventral hernia repair, Incisional Hernia Repair, Umbilical Hernia Repair, Surgical Procedures, Minimally Invasive Surgery, Laparoscopy/methods, Laparoscopic Ventral/Incisional hernia Repair, SILS, Single Port, Surgical Mesh, SPA, Single Port Access

**Searching machines:**

PubMed, Embase, Medline, and Cochrane Library (2003–2017) were searched for studies for potential inclusion.

**New publications:**

In addition to studies included in the published guidelines, 2 new and relevant studies were found included in the update. Both studies are Level 4, which supplement our knowledge of use of single-port surgery for ventral and incisional hernia repair.

New Statement: No new statements

Recommendations: No new recommendations

**Comments:**

In 2012, Tran et al. published a series of 22 patients who underwent laparoendoscopic single-site (LESS) ventral hernia repair [1]. This included 17 ventral/incisional, 2 parastomal and 1 suprapubic hernia, and 2 multiple recurrent inguinal hernias. Patients had a mean BMI of 31.5 kg/m2. Mean operative time was 125 min for ventral/incisional and 270 min for parastomal hernias. No conversions were reported. Minimum follow-up was 6 months, going up to 18 months in some patients. No recurrence was reported. They concluded that LESS ventral hernia repair is safe and feasible in experienced hands with acceptable morbidity. Downes et al. in 2016, in his series of 3 patients (1 primary and 2 incisional), demonstrated similar findings in a day case setting using single-incision laparoscopic surgery for ventral hernia repair [2].

With the advent of minimal invasive surgery, more surgeons today focus on improving cosmesis, reducing pain, and postoperative complications. In order to achieve these objectives, concept of reduced port surgery and single-port surgery has gained wider acceptability. Though cosmetic advantage makes it an attractive alternative to standard laparoscopy, benefits of the same with regard to clinical outcomes and postoperative complications and morbidity, incidence of port-site hernia are still debated. Better evidence in the form of randomized control studies with longer follow-up and large patient groups is needed before making any new recommendations for the use of single-port surgery for ventral or incisional hernia repair.

**References** (in parentheses graduation of evidence)

1. Tran HM (2012) Laparoendoscopic single site ventral hernia repair: demonstrated safety and efficacy. JSLS 16(2):242–9. PMC 3481233. (4)
2. Downes RO (2016) Single incision laparoscopic primary and incisional ventral hernia repair as the standard of care in the ambulatory setting; Does less equal better outcomes; Case series and literature review. Int J Surg Case Reports 26:73–76. PMCID: PMC4961682 (4)

